# Structural basis for a filamentous morpheein model of human cystathionine beta-synthase

**DOI:** 10.1038/s41467-026-73198-7

**Published:** 2026-06-06

**Authors:** Inayathulla Mohammed, Ela Mijatovic, Thilo Magnus Philipp, Lucia Janickova, Kelly Ascencao, Francisco J. Asturias, Luis Alfonso Martinez-Cruz, Csaba Szabo, Henning Stahlberg, Tomas Majtan

**Affiliations:** 1 Laboratory of Biological Electron Microscopy, Institute of Physics, School of Basic Sciences, Ecole Polytechnique Federale de Lausanne, Lausanne, Switzerland; 2 Laboratory of Biological Electron Microscopy, Department of Fundamental Microbiology, Faculty of Medicine and Biology, University of Lausanne, Lausanne, Switzerland; 3 Dubochet Center for Imaging Lausanne, University of Lausanne, Lausanne, Switzerland; 4 Section of Pharmacology, Department of Oncology, Microbiology and Immunology, University of Fribourg, Fribourg, Switzerland; 5 Department of Biochemistry and Molecular Genetics, University of Colorado Anschutz Medical Campus, Aurora, CO USA; 6 Center for Cooperative Research in Biosciences (CIC bioGUNE), Basque Research and Technology Alliance (BRTA), Derio, Spain

**Keywords:** Cryoelectron microscopy, Hydrolases, Enzyme mechanisms, Metalloproteins

## Abstract

Human cystathionine beta-synthase (CBS) is a vital enzyme that regulates sulfur amino acid metabolism, hydrogen sulfide production, and cellular redox balance. Using a multidisciplinary approach, we demonstrate that CBS functions as a filamentous morpheein, with its stability, turnover, and activity governed by dynamic quaternary structural transitions. Three distinct filamentous assemblies were resolved by cryo-EM and are mediated by the oligomerization loop (residues 516–525): (i) ligand-free *trans*-dimers that form *trans*-basal filaments with basal stability and activity, (ii) adenosylornithine-bound *cis*-dimers that assemble into stabilized *cis*-basal filaments and (iii) S-adenosylmethionine-bound *allo*-dimers, which, together with *cis*-dimers, form highly stable, *allo*-activated stacked filaments. These reversible filamentous assemblies redefine CBS biology by integrating oligomerization and allosteric regulation within a morpheein framework. These findings provide a transformative perspective on CBS function and open avenues for pharmacological targeting of dysregulated CBS in various diseases including homocystinuria, cancer, and Down syndrome.

## Abstract (web summary)

Human cystathionine beta-synthase (CBS) is shown to form distinct morpheein-style filamentous assemblies that regulate enzyme stability and activity in response to allosteric ligand binding, providing insights into CBS biology.

## Introduction

**Fig. 1:**
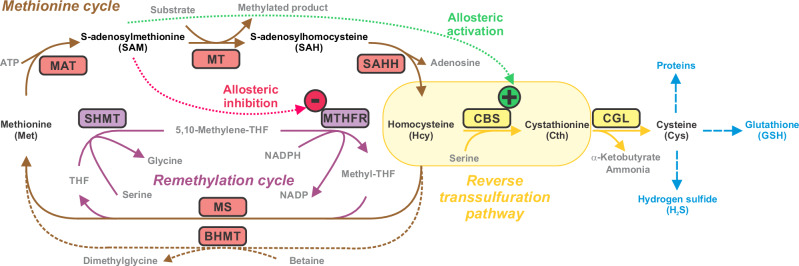
Role of CBS in sulfur amino acid metabolism. As a sole source of organic sulfur, dietary essential amino acid methionine (Met) is converted through a series of enzymatic reactions, where CBS plays a key role in diverting flux of sulfur through the competing methionine cycle (brown arrows) and the reverse transsulfuration pathway (yellow arrows). Met and ATP are condensed by methionine adenosyltransferase (MAT), yielding S-adenosylmethionine (SAM). As a universal methyl donor, SAM is used by multiple methyltransferases (MT), generating a methylated product (e.g., DNA, protein) and S-adenosylhomocysteine (SAH). As a competitive inhibitor of MTs, SAH is quickly hydrolyzed by SAH hydrolase (SAHH), yielding adenosine and homocysteine (Hcy). Hcy lies at the junction of two competing pathways where CBS serves as a gatekeeper and SAM as a master regulator. Specifically, Hcy can be remethylated back to Met through either a liver-specific shunt catalyzed by betaine homocysteine methyltransferase (BHMT) or ubiquitous methionine synthase (MS), which uses methyl tetrahydrofolate (methyl-THF) as a methyl group donor (solid and dashed brown arrows). The MS cofactor methyl-THF is then regenerated from THF via the remethylation cycle (purple arrows) involving serine hydroxymethyltransferase (SHMT) and methylenetetrahydrofolate reductase (MTHFR). Alternatively, Hcy is irreversibly diverted from the methionine cycle by CBS, catalyzing its condensation with serine, yielding cystathionine (Cth; yellow bubble) as the first step of the reverse transsulfuration pathway. Afterwards, cystathionine gamma-lyase (CGL) hydrolyzes Cth into alpha-ketobutyrate, ammonia and cysteine (Cys), which serves in protein synthesis and as a rate-limiting substrate in glutathione (GSH) and hydrogen sulfide (H_2_S) biogenesis (blue dashed arrows). Importantly, SAM regulates the organic sulfur homeostasis by allosteric activation of CBS (green dotted arrow), while allosterically inhibiting MTHFR at the same time (red dotted arrow).

Methionine (Met), an essential sulfur-containing amino acid (SAA), is indispensable for protein synthesis and serves as the primary source of biological sulfur required in numerous biological processes^1,2^. Its metabolism involves four key steps (Fig. 1): (i) conversion to S-adenosylmethionine (SAM), a universal methyl donor for methylation reactions, (ii) transfer of a methyl group to various substrates producing S-adenosylhomocysteine (SAH), (iii) hydrolysis of SAH to form homocysteine (Hcy), a non-proteinogenic SAA intermediate of the methionine cycle, and (iv) remethylation of homocysteine back to methionine^3^. A critical aspect of Met catabolism is the transfer of sulfur from homocysteine to serine, which drives the synthesis of cysteine (a semi-essential SAA), glutathione (the main cellular small molecule redox regulator) and hydrogen sulfide (H_2_S, a gaseous signaling molecule with regulatory roles in the cardiovascular, nervous and immune systems)^4^. This sulfur transfer, catalyzed by cystathionine beta-synthase (CBS), represents the first step of the reverse transsulfuration pathway (Fig. 1).

Human CBS is a pyridoxal-5′-phosphate (PLP)-dependent, heme-containing enzyme that catalyzes the condensation of homocysteine with serine to form cystathionine^5^. Downregulation or loss of CBS activity, primarily due to missense mutations within the CBS gene, results in classical homocystinuria (HCU), the most common inborn error of SAA metabolism characterized by a massive elevation of Hcy in tissues and circulation affecting the ocular, skeletal, vascular and nervous systems^6^. Additionally, low substrate specificity of CBS enables alternative reactions, notably the production of H_2_S through condensation of cysteine and homocysteine^7^, making CBS one of the three enzymatic sources of this key gaseous signaling molecule^4,5^. Upregulation of CBS leading to overproduction of H_2_S supports proliferation and bioenergetics in several types of cancer^8–10^ and contributes to metabolic and neurological deficits in Down syndrome^11,12^. A recent study links CBS upregulation in the development of colon cancer to the proteolytic removal of its autoinhibitory C-terminal regulatory domain^13^.

**Fig. 2:**
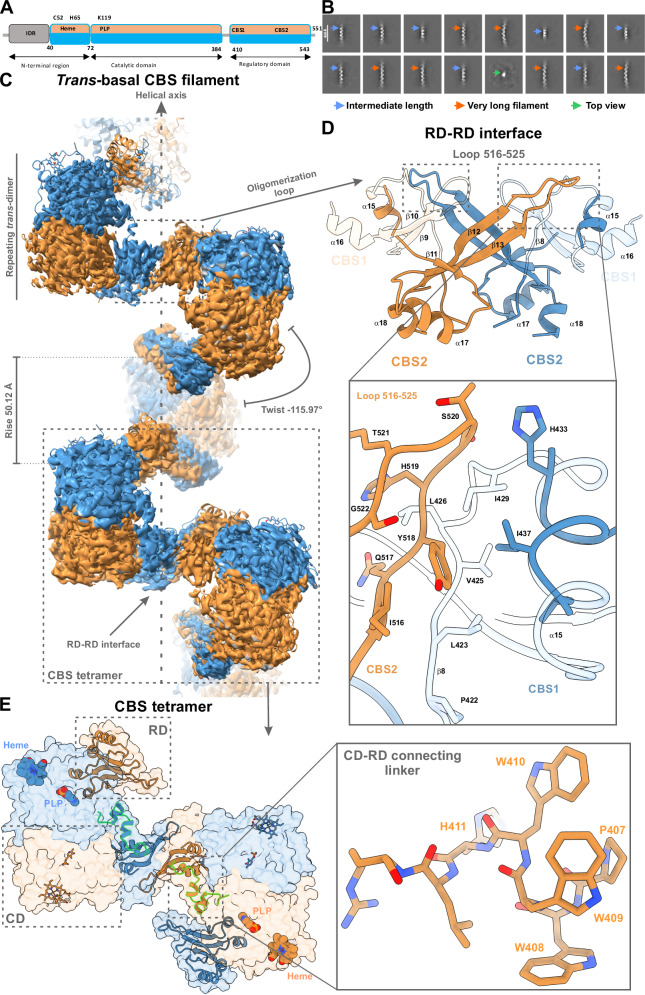
Architecture and inter-domain interfaces of the human CBS *trans*-basal filament. **A** Schematic representation of human CBS domain organization. **B** Representative 2D class averages of human CBS filaments in the absence of allosteric ligands, illustrating the length heterogeneity of the filamentous assemblies (610 Å scale bar). **C** Helical 3D reconstruction of the *trans*-basal CBS filament resolved to a global resolution of 3.0 Å. The inset shows an oblique view of the filament, highlighting its left-handed helical geometry with a twist of −116° and axial rise of 50 Å per asymmetric unit. The repeating unit is a *trans*-dimer composed of two protomers (blue and orange), arranged with D1 symmetry orthogonal to the helical axis. Oligomerization is mediated by interactions between the regulatory domains (RDs) of *trans*-dimers, interconnected through the oligomerization loop. **D** Close-up view of the RD–RD interface between adjacent CBS dimers showing contacts between the CBS2 oligomerization loop (residues 516–525, orange) and the CBS1 motif, including α-helix 15 (blue) of the neighboring protomer. The inset highlights residues I516 and Y518 from the loop inserting into a hydrophobic pocket formed by L423, V425, I429, I437, and H433 on α-helix 15, anchoring the CBS2 motif against CBS1. Additional stabilization is provided by backbone interactions involving β-strand 8 and neighboring residues, including P422, which adopts a reoriented conformation relative to truncated CBSΔ516–525 structures. Together, these inter-dimer contacts, centered on the oligomerization loop and α-helix 15 interface, form a key structural element that drives assembly and stabilization of the *trans*-basal CBS filament under ligand-free conditions. **E** Space-filled cryo-EM density representation and atomic model illustrating how *trans*-dimers organize into tetrameric assemblies (dimers of dimers) via head-to-tail interactions. The inset provides a close-up view of the linker region connecting the catalytic (CD) and regulatory (RD) domains (residues 384–414). A cluster of tryptophan residues, situated between H411 and P407, are positioned to facilitate allosteric signaling between the CD and RD, highlighting their potential involvement in conformational transitions and allosteric activation of CBS.

Human full-length CBS polypeptide has three functional domains: (i) an N-terminal domain (residues 1–70) containing residues C52 and H65 that coordinate the ferric heme-b cofactor, (ii) a central catalytic domain (CD, residues 70–381) with the evolutionary conserved K119 residue binding the PLP cofactor via a Schiff bond and (iii) a C-terminal regulatory domain (RD, residues 414–551), that upon binding SAM activates CBS by 3–9-fold (Fig. 2A)^14–18^. When studied using standard biochemical and molecular assays, CBS polypeptides usually form tetramers and higher-order oligomers, complicating purification and limiting structural insights into CBS biology^19^. An initial structural understanding came from a truncated dimeric variant lacking the RD (CBSΔ414–551 or CBS45), which revealed the arrangement and conformation of a heme-binding pocket and PLP-containing catalytic center of the enzyme^14,15^. Over a decade later, an engineered dimeric construct missing residues 516–525 (CBSΔ516–525) clarified the structure of the RD and revealed how SAM-induced conformational changes drive CBS activation^16–18^. However, the dimeric constructs used in these experiments could not explain the tetramer and higher-order oligomer formation characteristic of the full-length enzyme. Recently, McCorvie et al.^20^ reported that a full-length human CBS WT forms filaments with distinct conformations in the absence and presence of SAM. Despite this progress, the mechanisms governing CBS oligomerization and allosteric regulation remain poorly understood.

It has been hypothesized that human CBS is a morpheein, a homo-oligomeric protein whose function is regulated by reversible transitions between distinct, non-additive quaternary structures^21,22^. Using a multidisciplinary approach that combines cryo-electron microscopy (cryo-EM), biochemistry, molecular biology and confocal imaging, we demonstrate here that human CBS is indeed a morpheein. CBS undergoes significant conformational changes in its tertiary structures, triggered by the allosteric ligands, to form distinct filaments that control its cellular turnover, stability and activity. These findings resolve the long-standing mystery of human CBS oligomerization and activation by SAM.

## Results

### Human CBS is a filamentous enzyme

Recombinantly expressed, purified human full-length CBS in the absence of SAM (i.e., the basal state) formed ordered filaments with variable length (Fig. 2B and Supplementary Fig. 1). The cryo-EM helical analysis yielded a reconstruction at a resolution of 3.0 Å, revealing a left-handed helical architecture with a helical twist of −116° and a rise of 50 Å per subunit (Fig. 2C and Supplementary Fig. 2). The fundamental repeating unit of the filament is a *trans*-CBS dimer, i.e., a dimer, in which the RDs are domain-swapped relative to their respective CDs. This conformation resembles that of a dimeric CBSΔ516–525 variant previously solved by X-ray crystallography in the absence of SAM^16^. A crucial structural feature facilitating filamentous assembly is the oligomerization loop spanning residues 516–525 (Fig. 2D). This loop mediates inter-dimer interactions resulting in the formation of higher-order tetrameric units (Fig. 2E). These tetramers act as modular building blocks that extend to form filaments composed of up to 20 such units (Supplementary Fig. 3). The head-to-tail arrangement of dimers along the central axis, stabilized by the 516–525 loop, defines the oligomeric state referred here to as the *trans*-basal CBS assembly (Fig. 2C). Two key interfaces between RDs contribute to the stabilization of *trans*-basal CBS filament. The first involves interactions between the CBS1 and CBS2 motifs of adjacent subunits, bridged by the 516–525 loop and α-helix 15 (Fig. 2D). Additional hydrogen bonds and main-chain contacts between β-strands 12 and 8 further strengthen this interface. Within this context, residue Y518 is positioned into a hydrophobic pocket formed by residues within α-helix 15. The second interface consists of hydrophobic packing interactions between residues of adjacent CBS2 motifs, specifically involving L419, L492, M529, and F531. These contacts induce a reorientation of A421 and P422 relative to their positions in the crystal structure of CBSΔ516–525^16^. Altogether, these inter-dimer interactions, mediated by the oligomerization loop 516–525, are critical for the assembly and stabilization of the *trans*-basal CBS filament under ligand-free conditions.

### The oligomeric status of CBS determines its cellular turnover

**Fig. 3:**
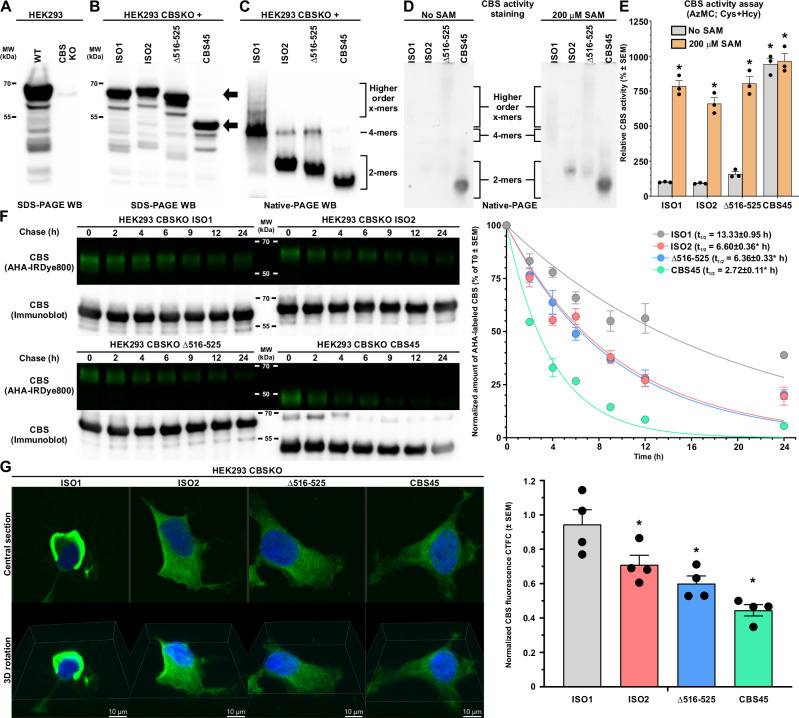
Cellular turnover of CBS is determined by its oligomeric status. **A** SDS-PAGE Western blot confirming the absence of endogenous CBS in HEK293 CBSKO cells (*n* = 3). **B** SDS-PAGE and **C** native PAGE Western blots of cell lysates showing expression and oligomeric status, respectively, of the studied CBS constructs (*n* = 3). **D** In-gel CBS activity staining and **E** AzMC-based fluorescence assay in cell lysates in the absence or presence of 200 µM SAM (biological *n* = 3). Data are presented as mean values ± SEM and were analyzed with one-way ANOVA followed by Tukey’s multiple comparison test (*p < 0.0001 for all compared to ISO1 in the absence of SAM). **F** Determination of cellular turnover of the studied CBS constructs showing representative gels and blots (left) used for signal quantification, normalization, plotting of the CBS decay (right) and calculation of CBS half-life (inset) (biological *n* = 5). Data are presented as mean values ± SEM, and the resulting half-lives were analyzed with one-way ANOVA followed by Tukey’s multiple comparison test (*p < 0.0001 for all compared to ISO1). **G** Confocal imaging of the studied CBS constructs showing their cellular compartmentalization (green = CBS, blue = nucleus; left) and quantification of the normalized CBS signal (right) (biological *n* = 4). Data are presented as mean values ± SEM and were analyzed with one-way ANOVA followed by Tukey’s multiple comparison test (*p value equals 0.037, 0.003, and <0.0001 comparing ISO1 to ISO2, Δ516–525, and CBS45, respectively).

Filamentous organization, a feature common among certain metabolic enzymes, has been shown to provide functional advantages, such as rapid deployment, catalytic enhancement or structural stabilization^23^. Given that both CBS WT and CBSΔ516–525 exhibit comparable catalytic activities in the absence and presence of SAM^16^, we hypothesized that filamentation may contribute to CBS stabilization in a cellular milieu. To test this hypothesis, we utilized a nonradioactive pulse-labeling method to assess cellular turnover of two annotated CBS isoforms along with two engineered constructs^24^. HEK293 cells lacking endogenous CBS, achieved via CRISPR-Cas9-mediated knockout (Fig. 3A), were stably transfected to express full-length CBS WT (isoform 1, ISO1), CBS isoform 2 containing an additional 15 residues replacing Y518 encoded by an alternative exon 15 (ISO2), CBSΔ516–525 and CBS45 (Fig. 3B). Notably, ISO1 predominantly formed tetramers and higher-order oligomers, whereas ISO2, CBSΔ516–525, and CBS45 primarily existed as dimers (Fig. 3C). This observation highlights the critical role of residue Y518, which fits into a hydrophobic pocket within α-helix 15 of the adjacent subunit, in promoting CBS filamentation (Fig. 2D). All constructs retained catalytic activity and responded to SAM except for CBS45, which does not bind SAM due to the lack of the RD and is constitutively activated (Fig. 3D, E). More importantly, full-length oligomeric CBS WT (ISO1) showed a cellular half-life of 13.3 h, which was approximately twice that of ISO2 and CBSΔ516–525 (6.6 and 6.4 h, respectively) and nearly five times longer than the 2.7-h half-life of CBS45 (Fig. 3F). These findings suggest that the tetramer observed on native PAGE corresponds to a functional assembly unit (a dimer of dimers) required for filamentation, while the predominant dimer correlated with impaired filamentation. Confocal microscopy analysis further supported these findings. Tetrameric/filamentous CBS WT (ISO1) exhibited concentrated fluorescence near the nucleus, whereas dimeric forms (ISO2, CBSΔ516–525, CBS45) showed a diffuse cytoplasmic distribution (Fig. 3G). Collectively, these results indicate that CBS tetramerization (and, by extension, filamentation) not only reduces protein turnover but also influences subcellular localization, thereby enhancing the enzyme’s stability and function in the cell.

To further strengthen support for the role of the oligomerization loop in CBS filamentation, we perturbed the length of the loop as well as key residues within the loop by using targeted mutagenesis. First, we increased and decreased the original ten-residue-long 516–525 loop deletion by two residues and prepared CBSΔ515–522 and CBSΔ515–526 with eight- and twelve-residue-long deletions, respectively. Both predominantly formed native dimers (Supplementary Fig. 4A) and retained regulation by SAM (Supplementary Fig. 4B), like CBSΔ516–525 (Fig. 3C, E). Human CBS can experimentally be activated by thermal pre-treatment presumably due to the partial denaturation of the RD and alleviation of the autoinhibitory block on the enzyme^20,25–27^. While filamentous CBS WT showed half-activation at 53.2 °C, all the oligomerization loop deletion variants displayed similar response to heating at significantly lower temperatures from 47.4 to 49.2 °C (Supplementary Fig. 4C). Thermal stability assessment using thermal shift assay essentially showed similar results (Supplementary Fig. 4C). These results indicate that dimeric loop deletion variants are more prone to thermal denaturation and consequently less stable compared to tetrameric/filamentous full-length CBS WT. Importantly, point mutagenesis had a similar effect on CBS oligomeric status as deletions within the oligomeric loop. Specifically, mutagenesis of key Y518 or adjacent Q517 and I516 (Fig. 2D) individually or all together into glycine caused predominant formation of dimers similar to CBSΔ516–525 (Supplementary Fig. 5A) without affecting regulation by SAM (Supplementary Fig. 5B). Furthermore, the oligomerization loop point mutants showed thermal half-activation at significantly lower temperatures (∼47 °C) compared to CBS WT (51.1 °C; Supplementary Fig. 5C). Lastly, the dimeric oligomerization loop point mutants showed significantly faster cellular turnover ∼7.0–10.5 h compared to filamentous CBS WT (15.8 h; Supplementary Fig. 5D). These findings suggest that disruption of CBS filamentation by changing the length of the loop or mutating the key residues results in the formation of CBS dimers with decreased stability compared to CBS WT.

### Substrate binding reveals catalytic rearrangements of the active site

**Fig. 4:**
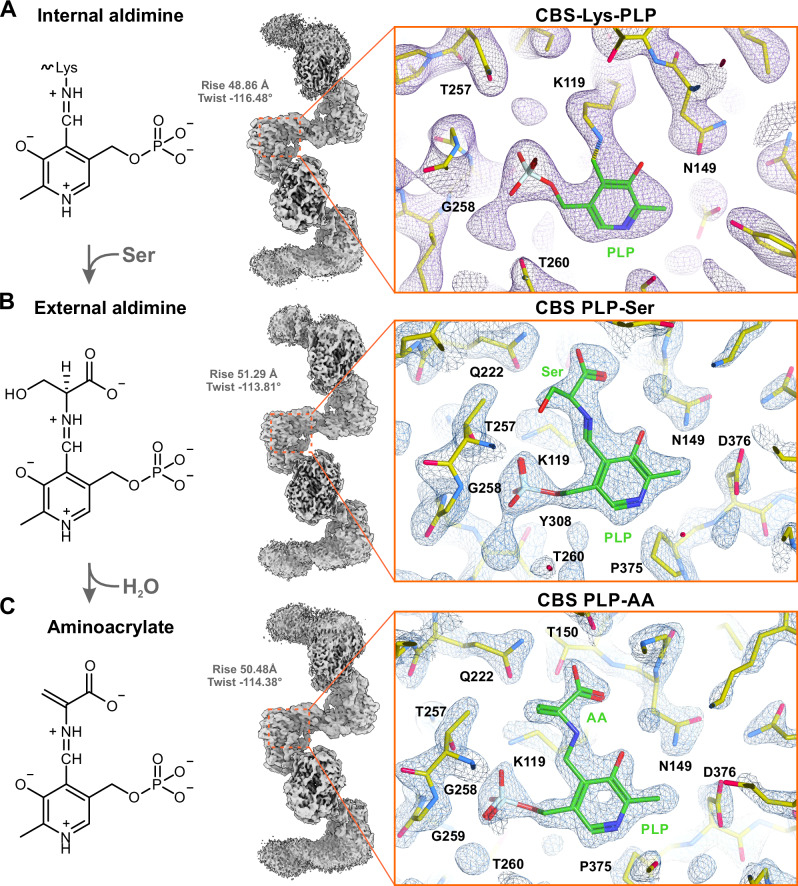
Substrate binding induces active site remodeling and catalytic intermediate formation in CBS. Cryo-EM structures capture sequential PLP-dependent intermediates: **A** internal aldimine, in which PLP is covalently linked to a conserved K119 residue (CBS-Lys-PLP), **B** serine-bound external aldimine (CBS PLP-Ser) captured at high resolution with clear density for the covalent PLP-Ser linkage and **C** dehydration of the external aldimine generates the PLP-aminoacrylate intermediate (CBS PLP-AA). Residues T257, T260, and G256–G259 stabilize PLP, while S147, T150, Q222, and N149 position serine. These rearrangements prime CBS for a nucleophilic attack by the second substrate (homocysteine) in the catalytic cycle.

To gain mechanistic insight into CBS catalysis, we determined cryo-EM structures of the enzyme in both the absence and presence of its substrate, L-serine (Fig. 4 and Supplementary Figs. 6–8). In its absence, the resting internal aldimine (CBS-Lys-PLP; Fig. 4A and Supplementary Fig. 9A) is anchored by the covalent Schiff-base linkage between K119 and PLP. The PLP phosphate is stabilized by a conserved hydrogen-bonding network involving T257 and T260, together with the backbone interactions from the G256–G259 loop (notably G256, G258, and G259). This network electrostatically stabilizes the phosphate group and fixes the PLP orientation, thus providing a stable reference point for subsequent chemistry. Surrounding residues, including N149, I306, S349, or P375/D376 and neighboring positions, such as E304 and G305, define the local electrostatic and steric environment of the pyridine ring and help shape the substrate channel and its entry. Upon addition of L-serine, the enzyme forms the external aldimine (CBS PLP-Ser; Fig. 4B and Supplementary Fig. 9B), in which the serine amino group replaces K119 in the Schiff base. In this state, serine is positioned and stabilized through a reinforced hydrogen-bond network involving S147, T150, and T146, as well as Q222 and N149, which together orient the serine hydroxyl and carboxylate groups for productive elimination. At the same time, phosphate stabilization by T257/T260 and the G256–G259 loop is maintained, indicating that the PLP anchoring network remains largely conserved while the reactive end of PLP undergoes chemical exchange. Subsequent dehydration yields the PLP-aminoacrylate intermediate (CBS PLP-AA; Fig. 4C and Supplementary Fig. 9C). In this state, the elimination of water is accompanied by additional tightening of the local hydrogen-bond network around the aminoacrylate moiety, while maintaining the strong electrostatic anchoring of PLP’s phosphate group. The aminoacrylate intermediate is stabilized in a more compact catalytic geometry, consistent with its role as the electrophilic intermediate that primes CBS for nucleophilic attack. The reaction proceeds through a β-replacement or α,β-elimination mechanism using the canonical substrate homocysteine and alternative H_2_S, respectively^7,28^. Transition from internal aldimine to the PLP-AA intermediate triggered a compaction of the active site, which occurred at the level of the entire catalytic core and was reflected in a global contraction of the catalytic pocket and coordinated repositioning of the residues involved in catalysis. This local active site remodeling coincided with an observable movement along the filamentous assembly of CBS. Specifically, subtle rocking-like motions centered around the CDs, transmitted through the flanking RDs along the filamentous axis, suggest a dynamic “breathing” behavior of the trans-basal CBS filament (Supplementary Movies 1 and 2). This conformational plasticity may represent an intrinsic feature of the enzyme’s catalytic cycle, potentially coupling local chemical events to long-range structural shifts across the filament.

### Non-activating allosteric ligand induces a distinct CBS filamentous state

**Fig. 5:**
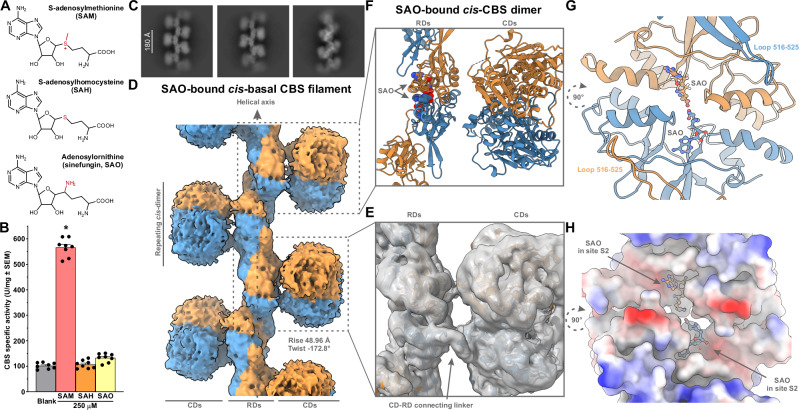
Structural characterization of the *cis*-basal CBS filament induced by the CBS non-activating allosteric ligand adenosylornithine (SAO). **A** Chemical structures of SAM, SAH and SAO, highlighting the key structural differences (in red). **B** CBS specific activity in the absence (blank) or presence of 250 µM allosteric ligands, demonstrating that SAO does not activate CBS (*n* = 8 independent repeats). Data are presented as mean values ± SEM and were analyzed with one-way ANOVA followed by Tukey’s multiple comparison test (*p < 0.0001). **C** Representative 2D class averages of CBS filaments assembled in the presence of SAO, showing a distinct morphology compared to the *trans*-basal filament observed in the absence of allosteric ligands (180 Å scale bar). **D** Helical cryo-EM reconstruction of the *cis*-basal CBS filament at an overall resolution of 4.0 Å. The inset shows an oblique view of the filament, highlighting its right-handed helical geometry with a twist of −173° and axial rise of 49 Å. The repeating unit is a *cis*-dimer composed of two protomers (blue and orange), where their RDs, forming a disc-shaped CBS module, reside atop their respective CDs. The overall architecture is defined by a central helical stalk formed by antiparallel CBS modules alternatively flanked by the CDs dimers on both sides. **E** A single *cis*-dimer 3D reconstruction focused on the flexible linker that acts as a hinge, enabling substantial conformational shifts. **F** Side view of the atomic model of a representative *cis*-dimer with bound SAO. **G** Close-up view of the CBS module with SAO bound at the site S2 located at the interface of the rearranged RDs from both protomers. The binding pocket is primarily stabilized by a hydrophobic patch formed by helix α17 (residues 536–549) from one subunit and helices α12 (residues 431–440) and α14 (residues 461–469) from the complementary subunit. Together with the oligomerization loop, this arrangement promotes the formation of a continuous central stalk. **H** Electrostatic surface potential map of the CBS module interface showing charge distribution contributing to RD–RD interaction of CBS module interface (red—electron-rich negative, blue—electron-poor positive charge/potential).

To investigate how binding of allosteric ligands affects the morphology of the CBS filament, first, we examined the impact of adenosylornithine (sinefungin, SAO), a CBS non-activating SAM analog (Fig. 5 and Supplementary Figs. 10, 12). Previous crystallographic study using CBSΔ516–525 E201S variant demonstrated that SAM binds to the S2 site within the RD with a 1:1 stoichiometry per monomer^17^. This interaction promoted a substantial rearrangement of the RDs into antiparallel, head-to-tail CBS modules. The structural reorganization, facilitated by a flexible interdomain linker (Fig. 2E), resulted in the spatial separation of the RDs from the CDs. However, it remained unclear whether the previously studied SAM analogs, such as S-adenosylhomocysteine (SAH) and SAO (Fig. 5A), could elicit similar structural changes^29^. The presence of SAH and SAO did not activate CBS (Fig. 5B), suggesting that mere occupation of the binding site may not be sufficient for the activation and that specific conformational rearrangements are essential to achieve that. Thus, we initially anticipated that SAO binding would not induce notable quaternary rearrangements. Contrary to this expectation, cryo-EM analysis of CBS WT in the presence of SAO revealed a filament morphology distinctly different from the *trans*-basal state (Fig. 5C). Due to filament flexibility, localized 3D refinement was necessary to obtain a composite global reconstruction at 4.0 Å resolution. The structure revealed a central helical core formed by antiparallel RD dimers flanked on either side by dimers of CDs. This repeating unit, distinct from the *trans*-dimer, was defined as the *cis*-CBS dimer (Fig. 5D–F). Interestingly, this architecture closely resembled that of the constitutively activated CBSΔ516–525 E201S in the SAM-bound state^17^, which previously led to the hypothesis that such a configuration may represent the activated form of full-length CBS^20^. Our current data, however, indicate that SAO binds to the same S2 site as SAM (Fig. 5F–H), but fails to activate the enzyme (Fig. 5B)^29,30^. Thus, despite the structural rearrangement, CBS remained catalytically unaltered in the presence of SAO. Accordingly, we designated this assembly as the *cis*-basal CBS filament. The *cis*-basal CBS filament exhibited a straight geometry with an overall right-handed helical twist of −173° and an axial rise of 49 Å. While the CDs retain minimal contacts with the central stalk consisting of the RDs, consistent with the previous activation models^17,20^, our structure revealed an absence of any functional coupling. Specifically, the lack of interactions between RDs and the flexible loops located near the catalytic entrance indicates that the additional structural determinants, likely specific to SAM-induced assemblies, are essential for CBS allosteric activation. Additionally, we also resolved a medium-resolution map capturing a transient intermediate during SAO binding (Supplementary Fig. 13A, B). This structure resembles a transitional state between the ligand-free *trans*-basal and SAO-bound *cis*-basal conformations, representing an intermediate step in the ligand-induced rearrangement.

### SAM binding facilitates CBS activation via filament stacking

**Fig. 6:**
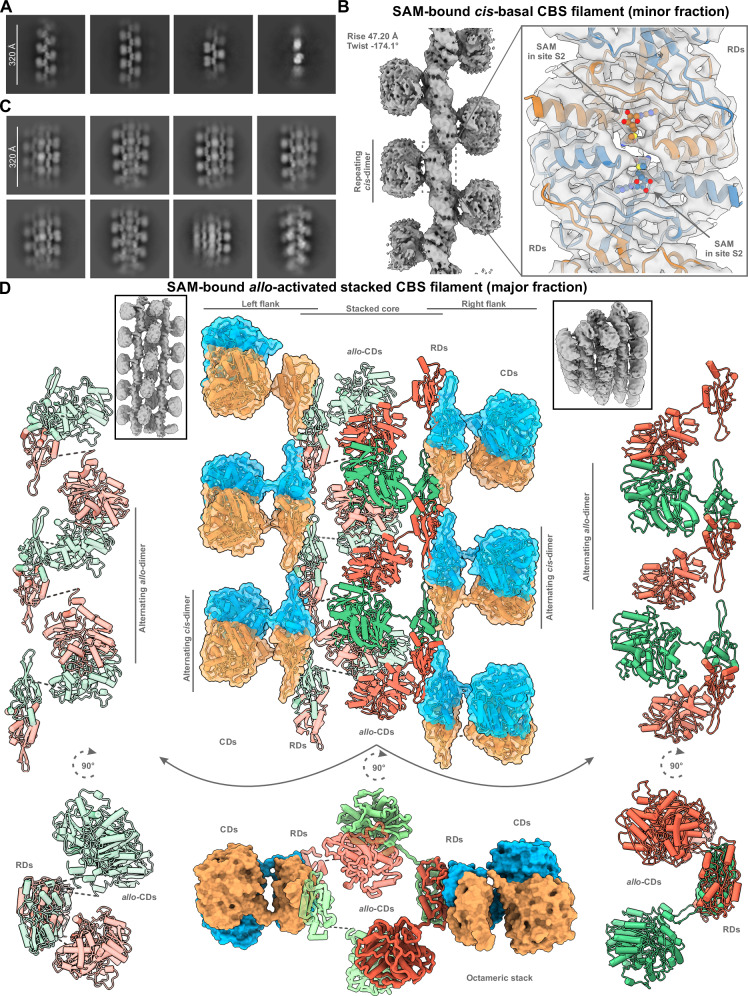
Structural characterization of SAM-induced CBS filamentous assemblies. **A** Representative 2D class averages of SAM-induced CBS filaments representing a minor population, displaying a morphology similar to the SAO-bound *cis*-basal CBS filaments (320 Å scale bar). **B** Helical cryo-EM reconstruction of the SAM-bound *cis*-basal CBS filament essentially identical to that of the SAO-bound *cis*-basal filament. Zoom-in view of the central stalk revealing SAM bound at the S2 site within the CBS module. **C** Representative 2D classes of the predominant population of SAM-induced CBS filaments, revealing the formation of highly compact, stacked assemblies distinct from both *trans*-basal and *cis*-basal states (320 Å scale bar). This distinct arrangement is referred to as the *allo*-activated stacked CBS filament. **D** Detailed architectural breakdown of the SAM-induced *allo*-activated stacked CBS filament. The inset on the left displays the overall 3D helical reconstruction of the SAM-bound *allo*-activated CBS assembly resolved at a global resolution of 7–10 Å. The inset on the right shows that this assembly exhibits a subtle right-handed helical twist, which is proposed to contribute to enhanced structural stability and dense packing. The entire quaternary structure can be broadly divided into the central stacked core and two flanking regions. The central core region comprises a distinct arrangement of *allo*-dimers (CBS protomers shown in alternating dark and light red and green colors), in which the catalytic domain (CD) dimers are contributed from protomers distinct from those forming the regulatory domain (RD) CBS module, unlike the canonical *cis*-dimer arrangement. Each left- and right-side flanking region contains *cis*-dimers, where CBS modules are aligned in alternating fashion with those of the core *allo*-dimers, forming two RD stalks via oligomerization loop-mediated interactions. To better illustrate the core architecture, two separate panels (the left and the right) depict the molecular details of the arrangement. The bottom panel (top-down perspective) highlights the intricate interweaving of CDs and RDs forming the octameric building block composed of two *cis*-dimers and two *allo*-dimers. This structural arrangement underlies the allosteric activation of CBS by SAM and represents a distinct quaternary configuration not seen in basal filament states.

To further investigate the mechanism of CBS allosteric activation, we explored the structural outcomes of CBS in the presence of its native allosteric ligand SAM. Given that the SAO-bound CBS structure challenged the prior models of CBS allosteric regulation, we sought to resolve whether SAM binding induces a comparable or distinct conformational state. Initial cryo-EM micrographs revealed that SAM binding triggered a reorganization of the *trans*-basal CBS filament into a configuration reminiscent of the SAO-bound *cis*-basal CBS filament (Fig. 6A). In this minor population, SAM was bound at the canonical S2 site within the RDs, similar to SAO (Fig. 6B and Supplementary Fig. 14). However, the major population captured in the presence of SAM represented a distinct filamentous assembly resembling two *cis*-basal filaments stacked in a zipper-like fashion (Fig. 6C and Supplementary Figs. 15–18). Due to the considerable size variation and conformational flexibility of these stacked assemblies, standard symmetry-based approaches were insufficient. Instead, multiple rounds of focused local refinements in combination with iterative helical reconstructions targeting individual substructures, including peripheral CDs, RDs forming the central stalks, and stacked layers of CDs, were employed. This strategy enabled us to reconstruct a composite model of the full assembly at a global resolution of 7–10 Å (Fig. 6D).

The major SAM-induced CBS assembly exhibited a distinctive quaternary conformation. A subtle right-handed helical twist of the stack was observed (Fig. 6D insets), likely contributing to the overall structural stabilization. Notably, within the central region of the stacked assembly, CD dimers were alternately contributed by the two opposing filaments and were slightly displaced outward to accommodate spatial constraints. Further analysis revealed that these centrally located CD dimers were structurally distinct from the peripheral CDs, which retained the *cis*-dimer arrangement as seen in the SAO-bound or minor SAM-bound structures (Figs. 5C, D, and 6A, B). Strikingly, the CBS dimer in the stacked core showed an association, wherein the CBS module and CD dimer were assembled from the protomers belonging to the opposing filaments. We refer to this distinct configuration as the *allo*-CBS dimer (Fig. 6D and Supplementary Fig. 13D). Each stacked unit is composed of two *cis*-dimers and two *allo*-dimers, forming an octameric assembly that extended into filaments through interactions mediated by the oligomerization loop (Fig. 6D). The *allo*-dimer likely contributes directly to the CBS activation by creating a catalytically favorable interface absent in *trans*-basal and *cis*-basal CBS filaments. Despite the moderate resolution limiting detailed interpretation of the catalytic site, the architecture of the central CD stack provides a plausible structural basis for SAM-induced activation. The observed inter-filament association, mediated by the distinct *allo*-dimers, appears to be essential for forming the catalytically competent state, which we define as the *allo*-activated stacked CBS filament. Supplementary Fig. 19 shows a series of superpositions (*trans*-basal, SAO-bound *cis*-basal, SAM-bound *cis*-basal and central *allo*-state protomers/dimers) using either the CD or the RD-loop region as alignment references, thereby quantifying how the transitions are dominated by rigid-body repositioning of the RDs relative to an essentially conserved CDs, and highlighting the large *cis*-to-*allo* rearrangement. This figure also emphasizes the CD-RD connecting linker as a plausible hinge coordinating CD-RD reorientation during assembly switching.

**Fig. 7:**
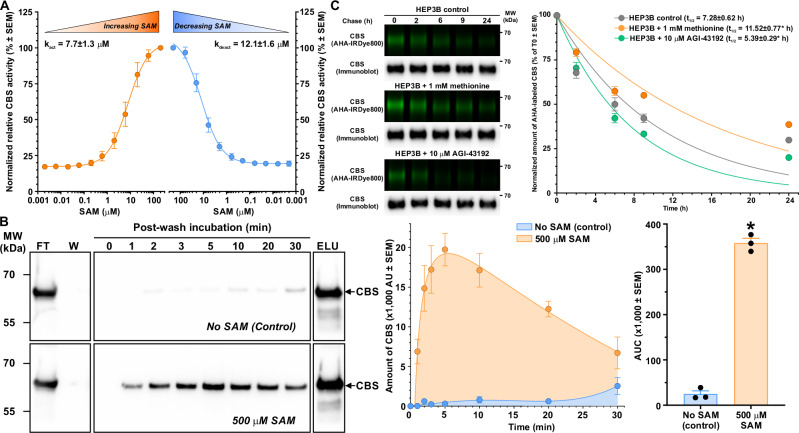
Biochemical characterization of CBS allosteric regulation by SAM. **A** Relative CBS activities in the presence of either increasing SAM up to 200 µM (green) or decreasing SAM content (red) illustrate the reversibility of CBS activation by SAM (*n* = 3 independent repeats). Data are presented as mean values ± SEM. **B** The SDS-PAGE Western blot (left) of major fractions (FT flow-through, W wash, ELU elution) that were collected during an immobilization assay, monitoring CBS filament dynamics at different time points. In the presence of 500 µM SAM, a marked dissociation of CBS filaments was observed, followed by reassociation into the *allo*-activated stacked CBS filament. Quantification (right) illustrates this ligand-induced disassembly and reassembly process (*n* = 3 independent repeats). Data are presented as mean values ± SEM and were analyzed with a two-tailed Student *t* test (*p < 0.0001). **C** Cellular turnover of the endogenous CBS in HEK293 cells is regulated by the intracellular content of SAM. Low SAM levels achieved by inhibition of MAT using 10 µM AGI-43192 resulted in a significantly reduced CBS half-life compared to control conditions (standard medium containing 200 µM methionine), while high levels of SAM achieved by 1 mM methionine supplementation of the culture medium substantially stabilized and increased CBS half-life. Representative gels and blots are shown on the left, were used to quantify CBS decay kinetics (right), with corresponding half-lives calculated (inset) under each condition (biological *n* = 3). Data are presented as mean values ± SEM, and the resulting half-lives were analyzed with ANOVA followed by Tukey’s multiple comparison test (*p value equals 0.0003 and 0.0264 comparing HEP3B controls to the treated ones with 1 mM methionine and 10 µM AGI-43192, respectively).

The co-existence of multiple quaternary states raised key questions about their reversibility and physiological relevance. Enzymatic assays demonstrated that the CBS activation by SAM is reversible: a titration of SAM up to 200 µM resulted in a half-maximal activation constant (*k*_act_) of 7.7 µM. Conversely, pre-incubation with 200 µM SAM, followed by a gradual dilution, led to CBS deactivation with a *k*_deact_ of 12.1 µM (Fig. 7A). This reversibility is consistent with the dynamic nature expected for metabolically regulated allosteric enzymes. To elucidate the structural transition pathway, we employed a Ni-NTA magnetic bead immobilization assay. In the absence of SAM, His-tagged CBS remained stably immobilized on the beads. Upon addition of 500 µM SAM, a rapid release of CBS into the solution was observed, followed by a gradual decline (Fig. 7B). These results suggest that SAM binding promotes disassembly of the *trans*-basal filament, followed by a facilitation of reassembly into *cis*-basal and *allo*-activated stacked CBS filamentous conformations. Interestingly, a similar experiment carried out using His-tagged dimeric CBSΔ516–525 did not lead to a release of protein into the solution, indicating that CBS dimers could conformationally rearrange upon SAM binding without detaching from Ni-NTA beads (data not shown). Together, these basic but informative assays are well in agreement with the proposed morpheein-style dynamics of CBS, while the C-terminal 6xHis tag remains sufficiently exposed to mediate interaction with the Ni-NTA matrix. To further investigate the biological relevance of SAM-induced stacking, we monitored endogenous CBS stability in hepatoma HEP3B cells under variable SAM conditions (Fig. 7C). Under basal conditions, CBS exhibited a half-life of 7.3 h. This increased to 11.5 h, a substantial 58% increase, after 1 mM methionine supplementation, raising the intracellular SAM concentrations. In contrast, the inhibition of SAM synthesis using Met adenosyltransferase inhibitor AGI-43192 (10 µM) reduced the CBS half-life by 26% to 5.4 h compared to the basal conditions. Together, these findings support a model in which SAM triggers CBS activation through stacking mediated by the distinct *allo*-dimer complemented by filamentation mediated by the oligomerization loop of CBS WT. This transition not only enhances enzymatic activity but also stabilizes the protein within the cellular environment.

### Pathogenic mutations disrupt the assembly and stability of CBS conformations

Several pathogenic CBS missense mutations causing HCU are located within the RD, particularly P422L and Q526K, which are predicted to interfere with CBS filamentation (Fig. 2D and Supplementary Fig. 20). When purified to homogeneity, these two pathogenic CBS variants were found indistinguishable in their oligomeric state (Supplementary Fig. 20A), activity-wise and SAM response-wise from CBS WT (Supplementary Fig. 20B). However, when subjected to thermal activation assay, CBS P422L showed half-activation at significantly lower temperature than CBS WT (43.7 vs. 53.2 °C), while on the other hand, CBS Q526K showed half activation at a significantly higher temperature without reaching an obvious plateau (56.6 °C; Supplementary Fig. 20C). Similarly, thermal unfolding of CBS P422L occurred at significantly lower temperature in the absence of SAM compared to CBS WT (36.4 vs. 51.0 °C), while unfolding of CBS Q526K in the presence of SAM yielded a pattern consistent with formation of aggregates (Supplementary Fig. 20D). Preliminary cryo-EM analysis of these pathogenic variants supports the biochemical findings. Specifically, CBS P422L in the absence of SAM yielded only dimers and occasional tetramers without any trace of CBS filaments, while in the presence of SAM, CBS P422L remained largely dimeric/tetrameric with an additional population of short *cis*-basal filaments (Supplementary Fig. 20E). On the other hand, CBS Q526K formed *trans*-basal filaments in the absence of SAM, but the protein was prone to aggregation. Upon addition of SAM, the protein rapidly precipitated, leaving little soluble material suitable for grid preparation. The limited recovered fraction consisted predominantly of aggregates with only a weak indication of *cis*-basal-like assemblies (Supplementary Fig. 20E). Taken together, these initial data on HCU-causing CBS mutants indicate that pathogenic mutations in the oligomerization interface affect assembly and stability of filamentous CBS conformations.

## Discussion

Since its discovery and initial characterization, the oligomeric nature of human CBS has been a subject of considerable debate. Early studies variably described CBS as a heterotetramer or homodimer before converging on the widely accepted model of a homotetramer composed of four 63 kDa subunits^19,31–33^. However, recent findings from McCorvie et al.^20^ and our current cryo-EM data redefine CBS architecture, showing it to be a filamentous protein with repeating dimeric and tetrameric structural units (Fig. 2). Our findings represent a transformative advancement in understanding of human CBS structure-function relationships, directly linking filamentation to the enzyme’s stability, activity, and cellular turnover.

**Fig. 8:**
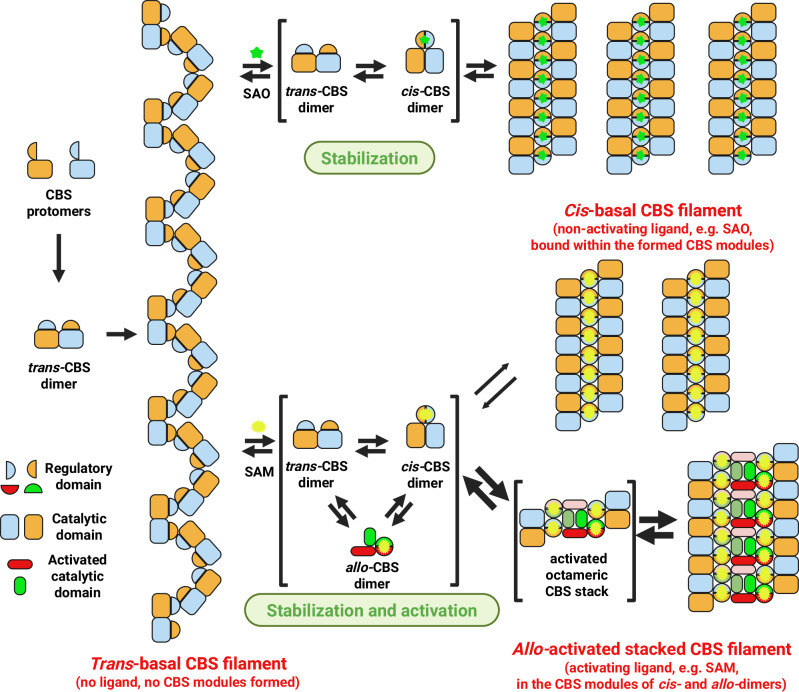
A morpheein-based model of CBS oligomerization and allosteric regulation. Nascent human CBS polypeptides (depicted in orange and blue) first assemble into *trans*-CBS dimers, where the regulatory domains (RDs, shown as half circles) are swapped and interact with the catalytic domains (CDs) of the opposing subunit. Oligomerization of these *trans*-dimers, mediated by the oligomerization loop (residues 516–525), results in the formation of the *trans*-basal CBS filament, which represents the CBS enzyme’s basal, ligand-free state. Binding of a non-activating allosteric ligand, such as adenosylornithine (SAO; green star), results in the dissociation of the *trans*-basal CBS filament, conformational rearrangement of the CBS subunits yielding *cis*-CBS dimers characterized by the formation of a ligand-stabilized CBS module between the RDs of the complementary subunits within the dimer. Subsequent oligomerization of these *cis*-dimers mediated by the same oligomerization loop gives the *cis*-basal CBS filament, characterized by increased stability and retains the basal enzymatic activity. Binding of an activating allosteric ligand, such as S-adenosylmethionine (SAM; yellow star), triggers a similar dissociation of the *trans*-basal CBS filament, but promotes a distinct conformational outcome: formation of *allo*-CBS dimers. These dimers exhibit a CBS module stabilized by RD interactions similar to those observed in *cis*-dimers; however, their CDs form dimers with CDs from adjacent *allo*-dimers, enabling an inter-dimer arrangement. Together with SAM-bound *cis*-dimers, these assemble into an octameric CBS stack composed of two *cis*-dimers and two *allo*-dimers. This stacked octamer serves as a nucleation seed for the formation of the *allo*-activated stacked CBS filament, a higher-order assembly characterized by high enzymatic activity and structural stability. The central CD dimers in this filament (shown in red and green) adopt a distinct conformation not seen in other CBS assemblies. The stacking and the filamentation occur concurrently, facilitated by the oligomerization loop (residues 516–525). Additionally, a minor population of SAM-bound *cis*-dimers associates independently of the *allo*-dimers, yielding a SAM-bound *cis*-basal CBS filament similar to those induced by the non-activating ligands like SAO. Human CBS enzyme thus exists in a dynamic equilibrium between these distinct quaternary states, modulated by both CBS concentration and the nature and availability of the allosteric ligand. Created in BioRender. Majtan, T. (2026) [https://BioRender.com/w2k1dzb].

Based on our structural and biochemical analysis, we propose a dynamic model of CBS oligomerization governed by the morpheein paradigm of allosteric regulation, in line with a model previously proposed by Jaffe^34^. According to this model, homo-oligomeric proteins transition reversibly between multiple quaternary states with distinct properties. These transitions require dissociation into lower-order species and a subsequent conformational change before reassembly. This framework explains the multifaceted kinetic behaviors and regulatory features observed in CBS. Filamentous human CBS exists in at least three distinct states: the ligand-free *trans*-basal filament (Fig. 2), the non-activating ligand-bound *cis*-basal filament (Fig. 5) and the activating ligand-bound *allo*-activated stacked filament (Fig. 6). Importantly, our findings show that both the *trans*-basal and *cis*-basal filaments exhibit basal catalytic activity, while only the *allo*-activated stacked filament formed through SAM-mediated *allo*-dimer association shows catalytic activation. This rules out the previous model proposing a gradual in situ RD rearrangement without filament disassembly^20^, which is incompatible with the structural homogeneity observed in our reconstructions. In our updated model depicted in Fig. 8, CBS dimers associate in an allosteric ligand-dependent mode. In the absence of ligands, RDs are unable to form stabilized CBS modules and instead undergo swapping with CDs of the opposing subunit, generating the *trans*-dimer. These *trans*-dimers assemble into *trans*-basal filaments via the *oligomerization loop* (residues 516–525) (Fig. 2). This assembly is identical to the previously reported cryo-EM structure of human CBS in the absence of allosteric ligands termed similarly as the basal filament^20^, although our structures provide (i) an improved structural resolution for the oligomerization interfaces, (ii) a direct linkage between filamentation state and cellular stability/turnover/localization (Fig. 3) and (iii) a framework for interpreting filament transitions induced by substrates and allosteric ligands (Figs. 4–6). The binding of non-activating ligands, such as SAO or SAH, facilitates the formation of ligand-stabilized rearrangement of CBS modules into a *cis*-dimer configuration, which further oligomerizes into a straight *cis*-basal filament (Fig. 5). In contrast, SAM induces the formation of a distinct *allo*-*dimer* that, along with *cis*-dimers, builds an *octameric stack*. This stack is extended into the catalytically competent *allo-activated stacked filament* through oligomerization loop-mediated interactions (Fig. 6). Although the intrinsically disordered N-terminus (first ∼40 residues) of human CBS has not been resolved in any of the previously published or herein presented structures, it is plausible that this segment could contribute to the stabilization of *allo*-activated stacked assemblies by, e.g., transient crosslinking or inter-stack contacts. The central *allo-CDs* in this assembly adopt an alternative geometry that likely enhances catalytic activity through effects on local crowding, diffusion, and protein dynamics^35^. In parallel, Supplementary Fig. 21 provides a stepwise schematic that connects these structural differences to the morpheein mechanism, illustrating the proposed dissociation of the *trans*-basal filament, formation of *cis*-dimers, emergence of *cis*/*allo* tetrameric intermediates, nucleation of an activated octameric stack, and subsequent elongation into the *allo*-activated stacked filament. This hierarchical assembly, requiring initial dissociation followed by reassociation, aligns well with a proposed morpheein framework. Importantly, stacking and filamentation appear to occur concurrently rather than sequentially, with the octameric stack acting as a structural seed that nucleates the formation of the *allo*-activated stacked filament (Fig. 6). This also explains why CBSΔ516–525, despite the lack of the oligomerization loop and inability to form filaments, is still activated by SAM via a transient formation of the octameric stack. These data on ligand-bound CBS filaments contrast with the previously reported cryo-EM structure of SAM-bound human CBS, where the authors did not use a non-activating ligand, such as SAH or SAO, and in the presence of SAM, obtained only one conformation identical to our SAO-bound *cis*-basal filament and named it the activated filament^20^. Although such observation resulted in a straightforward reconciliation of the previously reported structural and biochemical data^16–18,36^, our data reported here and depicted in the proposed model shown in Fig. 8 (further supported by Supplementary Fig. 21) clearly show that the actual structural landscape of human CBS is more complex involving differential conformational response to non-activating and activating allosteric ligands, forming *allo*-activated stacked filament in the presence of SAM and following morpheein-style dynamics. This represents the main structural difference from the previous report^20^.

Our model also reconciles the binding stoichiometry and affinities of CBS-ligand interactions reported by isothermal titration calorimetry^20,29,36,37^. Filamentous CBS WT showed biphasic binding curves for SAM consistent with two types of binding sites^37^, whereas engineered dimeric CBSΔ516–525 only exhibited a single site binding^36^. We now interpret these differences as a reflection of structural transitions, i.e., dimer rearrangement upon binding and formation of higher-order oligomeric assemblies, that depend on both the character of the allosteric ligand and the presence of the oligomerization loop. Additional supporting evidence comes from the studies employing differential scanning calorimetry, which showed that SAM binding substantially stabilized the RD, an effect significantly less prominent with non-activating ligands, such as SAH and SAO^29,36,37^.

Although earlier interpretations designated the *cis*-dimer as the activated form based on comparisons to structures of constitutively activated *Drosophila melanogaster* CBS and human CBSΔ516–525 E201S variant^17,38^, our findings reveal that activation of human CBS is driven only when SAM mediates formation of the *allo*- and *cis*-dimers, which yield an activated octameric stack, while the intact oligomerization loop facilitates formation of the *allo*-activated stacked CBS filament. These findings suggest that SAM not only stabilizes the CBS module, but—unlike non-activating ligands, such as SAH and SAO—also drives a specific spatial reconfiguration required for activation.

Filamentation of CBS may not be conserved across all species. The critical role of both the composition and the length of the oligomerization loop is further highlighted in Supplementary Fig. 22, showing sequence conservation for a limited number of species. While yeast and insect CBSs essentially lack the oligomerization loop (i.e., are similar in length to CBSΔ516–525 in the corresponding region), mammalian CBS proteins retain the same length as CBS WT and tyrosine residue stabilizing interaction with α-helix 15 (Fig. 2D). Notably, in some species, such as rat and mouse, the major CBS isoform is the one similar to human CBS ISO2. The elongated oligomerization loop introduces a small hydrophilic serine residue instead of a bulky aromatic tyrosine and consequently disrupts CBS filamentation, as we showed recently for mouse CBS^39^. The biological roles of filamentation in CBS likely mirror those seen in other metabolic enzymes^23^, such as providing regulatory flexibility^40,41^, enabling compartmentalization^42^ or protecting against degradation^43^. Indeed, our data show that non-filamentous dimeric CBS constructs exhibit significantly shorter cellular half-lives and altered sub-cellular localization compared to the filament-forming CBS WT (Figs. 3 and 7). Furthermore, CBS WT showed significant stabilization in the presence of methionine and, in contrast, destabilization in the presence of MAT inhibitor blocking biogenesis of SAM (Fig. 7C), likely due to a dynamic shift between less stable and less active basal filamentous assemblies (i.e., ligand-free *trans*-basal and non-activated ligand-bound *cis*-basal conformations) and SAM-stabilized *allo*-activated stacked CBS filament. These data correlate well with the previously reported CBS filamentation response to nutrient changes, showing predominant formation of larger CBS filamentous assemblies near the nuclei in the presence of methionine and/or SAM in culture media compared to smaller CBS puncta shown in their absence^20^.

The proposed morpheein-based model of CBS regulation also has significant pathophysiological implications. Homocystinuria (HCU) is an inborn error of metabolism and conformational disorder resulting from protein misfolding, rapid degradation and/or impaired regulation of pathogenic CBS variants^24,44–47^. Mutations that interfere with the rearrangement of CBS dimers, their assembly into higher order oligomers, impair filamentation or promote aggregation may drive the disease phenotype. Initial view characterizing pathogenic CBS P422L and CBS Q526K variants clearly supports this notion (Supplementary Fig. 20). Previous characterization of CBS P422L showed that this variant (along with two other pathogenic missense variants in the RD, namely CBS I435T and CBS S466L) migrated faster under native conditions than CBS WT indicating possibly a different CBS conformation associated with a constitutive activation and impaired oligomerization/filamentation^26^. Specifically, the basal catalytic activity of CBS P422L was ∼53% higher compared to CBS WT, and the variant displayed aberrant response to thermal activation when compared to activation by SAM. Interestingly, CBS I435T and CBS S466L variants showed faster migrating oligomeric forms and constitutive activation^26^, while their ability to bind SAM has not been impaired^37^. Considering the presented structural data, formation of filamentous assemblies is impaired by the pathogenic mutation, which in the case of the C-terminal variants may lead to SAM-independent activation, oligomeric impairment and conformational instability. Therapeutic strategies targeting CBS filamentation, either by stabilizing functional assemblies or promoting proper allosteric transitions, could offer innovative therapeutic avenues. Particularly, SAM analogs have been hypothesized to act as CBS-specific pharmacological chaperones rescuing the enzyme’s oligomerization, stability and activity^29,47,48^. Quantitative structure-activity relationships study on SAM analogs and proof-of-concept animal study indicate that it may be possible to design “stabilizing” or “activating” SAM analog, which could act as CBS-specific pharmacological chaperones rescuing CBS folding, stability and activity^49^. Anticancer drug Taxol, which stabilizes microtubules by locking beta-tubulin into a straight conformation even after GTP hydrolysis, normally leading to a curved conformation and depolymerization, thereby promoting assembly and blocking disassembly^50^, or morphlock-1, which inhibits the transition of porphobilinogen synthase from inactive hexamers to active octamers^51^, set a precedent for the feasibility and successful development of innovative pharmacological therapies regulating CBS. These may extend beyond HCU into conditions with dysregulated CBS, including wound healing, angiogenesis, Down syndrome and various types of cancer^12,13,52^.

In conclusion, our findings redefine CBS as a filamentous morpheein and establish assembly, stacking and conformational plasticity as central features of its regulatory landscape. This paradigm shift opens exciting possibilities for understanding and therapeutically modulating CBS function.

## Methods

### Preparation of human CBS protein

Full-length human CBS was expressed and purified as described previously^26,30^ with a few modifications. Briefly, *E. coli* Rosetta2 (DE3) cells transformed by pGEX-6P1-hCBS construct were cultured using twelve 2.8 l Fernbach flasks, each containing 1 l of LB medium supplemented with 0.001% thiamine HCl, 0.0025% pyridoxine HCl, 0.15 mM FeCl_3_, 3% ethanol, 0.3 mM delta-aminolevulinic acid and 100 µg/ml ampicillin. The cells were grown at 30 °C, shaking at 275 rpm until the OD_600_ reached ∼0.75. The expression of CBS was induced by the addition of 0.5 mM IPTG. After overnight expression, cells were harvested by centrifugation at 6500 × *g*, 4 °C for 5 min, pooled, washed with ice-cold 1× PBS, and immediately frozen at −80 °C. Later, the cells were resuspended in cold lysis buffer (50 mM sodium phosphate, pH 7.4, 300 mM NaCl, 1 mM DTT, 1% Triton X-100, 0.1 mM PLP, 1× Sigma Protease inhibitor cocktail) using a Dounce homogenizer and lysed with 2 mg/ml lysozyme while rocking at 4 °C for 1 h. After sonication to decrease the viscosity of the suspension, the insoluble fraction was separated by centrifugation at >25,000 × *g*, 4 °C for 30 min. The GST-CBS fusion protein was purified by loading the clarified lysate on the GST Sepharose column, washed with 10 mM sodium phosphate, pH 7.4, 500 mM NaCl, 1 mM DTT and eluted with a wash buffer containing 20 mM reduced glutathione. The GST fusion partner was cleaved off by 0.5 U HRV3C protease per milligram of protein at 4 °C overnight, followed by a separation of GST and CBS using 15–300 mM potassium phosphate, pH 7.2 gradient on DEAE Sepharose column. The CBS-rich fractions were pooled, concentrated and buffer exchanged into 20 mM HEPES pH 7.4, 1 mM TCEP and 0.01% Tween 20 using an Amicon YM-100 membrane. Purified CBS at concentrations typically ranging 10–20 mg/ml was stored at −80 °C in small (20–50 µl) aliquots.

### Cryo-EM

#### Four separate cryo-EM datasets were prepared and analyzed

(1) CBS WT in its basal state (*trans*-basal CBS), (2) CBS bound to its substrate L-serine (Ser-*trans*-basal CBS), (3) CBS bound to allosteric ligand sinefungin (adenosylornithine, SAO) (SAO-*cis*-basal CBS), and (4) CBS bound to SAM (SAM-*allo*-activated stacked CBS).

For all CBS datasets, concentrated CBS WT was diluted to 2.2 mg/ml in a 20 mM HEPES pH 7.2, 100 mM NaCl and 1 mM β-mercaptoethanol. The sample was applied to freshly plasma-cleaned gold Quantifoil R 1.2/1.3 grids (300 mesh) and vitrified using a Vitrobot Mark IV (ThermoFisherScientific) with 100% humidity at 10 °C, using a blot force of 0 and a blot time of 3.5–4.5 s. For the Ser-*trans*-basal CBS, 0.5 mM L-serine was added to CBS and incubated for 20 min on ice before grid preparation. Similarly, SAO-*cis*-basal CBS grids were prepared by incubating CBS with 1 mM SAO for 20 min. To capture SAM-*allo*-activated stacked CBS, CBS was incubated with 0.5 mM SAM for 10 min, generating samples with different stages of allosteric activation. All grids were prepared under identical controlled conditions, ensuring consistency in sample vitrification. Cryo-EM data for all four datasets were collected on Titan Krios G4 microscopes (ThermoFisherScientific) operated at 300 keV, equipped with a Falcon 4 direct electron detector operating in counting mode. For *trans*-basal CBS and Ser-*trans*-basal CBS datasets, a SelectrisX energy filter with a 10-eV slit width was used. Data acquisition was automated using EPU software (v3.0), utilizing aberration-free image shift (AFIS) for efficient high-throughput collection.

#### The *trans*-basal CBS (dataset 1)

For the *trans*-basal CBS conformation, a total of 4865 movies were recorded in Electron Event Representation (EER) mode, using a calibrated pixel size of 0.72 Å and a total electron dose of 72  e⁻/Å². After careful assessment and selection based on CTF estimation and overall image quality, 3530 movies were chosen for further processing. Initial motion correction and dose weighting were performed using the built-in tools in cryoSPARC (v4.4.1)^53^. The contrast transfer function (CTF) parameters were estimated on a per-micrograph basis^54^. For filament-based helical reconstruction, filaments were manually traced using cryoSPARC’s filament tracer module with an inter-box separation distance set at 32 Å. An initial set of approximately 1.2 million filament segments was extracted. Multiple iterative rounds of 2D classification were performed to remove false positives and damaged particles, leading to a high-quality subset containing 528,877 particles. This refined set was used for an ab initio reconstruction into four classes. Two well-defined classes were selected and combined for further processing (Supplementary Fig. 2). Initial helical parameters (twist and rise) were estimated using a combination of tools, including cryoSPARC’s symmetry search utility, HELIXPLORER, ab-initio volume reconstructions examined in UCSF Chimera^55,56^, and analysis of power spectra from elongated filament segments (Supplementary Fig. 3).

Subsequently, heterogeneous refinement with two classes was performed, and the best class was selected for high-resolution helical refinement. A final set of 335,525 particles was used, yielding an initial reconstruction at 3.4 Å resolution. Further iterative refinements were carried out using C1 and D1 symmetries separately, resulting in final maps at 3.2 and 3.0 Å, respectively. The final refined helical parameters were a twist of −116.11° with a rise of 49.9 Å for C1 symmetry, and −116.06° with a rise of 49.97 Å for D1 symmetry, respectively. For single-particle-like (SPA-like, non-helical) processing, an independent particle-picking strategy was employed. An initial set of approximately 1.4 million particles was extracted. Extensive rounds of 2D classification were performed to clean the dataset, ultimately retaining 437,000 high-quality particles. These particles underwent an ab initio 3D classification into three classes, from which two well-resolved classes were selected and merged. The merged particle set, consisting of 338,925 particles, was subjected to homogeneous refinement, nonuniform (NU) refinement, and local refinement steps^57^. Application of D1 symmetry during NU refinement and subsequent focused local refinement on the dimer–dimer interface significantly improved map quality. The resolution progressively improved from 2.89 Å (without symmetry, C1) to 2.63 Å when D1 symmetry was imposed, following final global CTF aberration correction and additional nonuniform and local refinements (Supplementary Fig. 2). The final high-resolution map allowed for detailed visualization of side-chain densities and confident atomic model building. Note that the intrinsically disordered region spanning the first ∼40 residues has not been resolved in any of our structures reported in this study, likely due to its flexibility. The numbering of secondary structure elements has been previously established when the crystal structure of the engineered human CBS construct lacking the oligomerization loop 516–525 has been resolved and followed in the field^16–18,20^. Supplementary Fig. 1 shows details about the CBS secondary structure elements and their designation.

#### The Ser-*trans*-basal CBS (dataset 2)

For the Ser-*trans*-basal CBS (dataset 2), a total of 14,954 movie stacks were collected in EER format, out of which 13,200 movies were selected after careful evaluation of CTF estimations and overall image quality. Data collection was performed at a nominal pixel size of 0.452 Å and with a total electron dose of approximately 52 e⁻/Å² per movie, allowing for fine sampling of high-resolution features. Using the filament tracer tool implemented in cryoSPARC, initial particle picking was carried out along the filaments with an inter-particle separation of 50 Å. This approach resulted in an initial set of approximately 2.18 million filament segments. Several iterative rounds of 2D classification were subsequently performed to remove poor-quality or contaminant particles, refining the dataset to around 1.2 million particles suitable for downstream processing. Following 2D cleaning, an ab initio reconstruction was performed with three initial classes. Two well-defined classes exhibiting clear secondary structure features were selected and merged for further helical reconstruction (Supplementary Fig. 6). Initial helical parameters, including twist and rise, were determined as described previously.

Approximately 1 million particles were subjected to helical refinement using both C1 and D1 symmetry constraints, yielding final resolutions of 2.68 and 2.70 Å, respectively. The optimized helical twist was refined to −114.6°, and the rise converged at approximately 49.9 Å. To further dissect the heterogeneous intermediates trapped during catalysis, several rounds of focused 3D classification were performed. Classification jobs with *K* = 5, 8, and 10 classes were run after anisotropic correction to separate distinct catalytic substates. After detailed analysis, three classes comprising a combined total of 577,766 particles were merged from *K* = 8 classification for additional focused refinement. These merged particles were also subjected to an SPA-like processing, where duplicates closer than 60 Å were excluded, followed by NU refinement and further helical refinement. This workflow yielded maps with resolutions of 2.34 and 2.65 Å for the major intermediate states (Supplementary Fig. 6). To further resolve local conformational differences, a final round of focused 3D classification was performed using custom masks spanning the full catalytic domain and flanking regulatory interfaces, using *K* = 5 classes and C1 symmetry (D1 symmetry was not imposed in any of the 3D classifications). The three best 3D classes were individually refined, revealing subtle conformational rocking motions of the RDs relative to the CDs. Final SPA-like refinements on these distinct classes led to the identification of three well-resolved substrates corresponding to (i) internal aldimine CBS-Lys-PLP refined to 2.72 Å resolution, (ii) external aldimine CBS PLP-Ser refined to 2.20 Å resolution and (iii) aminoacrylate CBS-PLP-AA refined to 2.00 Å resolution (Supplementary Fig. 7). Corresponding helical refinements with D1 symmetry constraints yielded consistent maps at 2.97 Å (twist −115.7°, rise 50.4 Å), 2.77 Å (twist −115.0°, rise 50.8 Å) and 2.54 Å (twist −115.3°, rise 49.9 Å), respectively (Supplementary Fig. 8). These final high-resolution maps allowed for unambiguous visualization of the ligand densities and precise side-chain positioning, facilitating confident atomic model building and enabling detailed mechanistic interpretation of each intermediate state. We used LigPlot+ graphical system for 2D visualization of ligand-protein interaction from 3D coordinates^58^.

#### The SAO-*cis*-basal CBS (dataset 3)

For the SAO-*cis*-basal CBS (dataset 3), a total of 14,300 movies were collected in EER format. Data collection was performed at a nominal pixel size of 0.52 Å, and the total electron dose was set at 50.0 e⁻/Å². Following initial motion correction and CTF estimation, 12,400 high-quality movies were retained for further processing. Particles were automatically picked using filament tracer in cryoSPARC with an inter-particle separation of ~50 Å. After the initial extraction, approximately 2.2 million filament particles were obtained. These underwent multiple rounds of 2D classification to discard broken or low-quality segments, resulting in a refined subset of 1.1 million particles. This refined subset was subjected to two rounds of ab initio reconstruction with three classes each, after which two well-defined classes were selected and merged. Further 2D classification was performed to isolate the best filament views, yielding a final particle set of 411,125 particles for helical refinement. Initial helical parameters, including twist and rise, were determined using cryoSPARC, HELIXPLORER, and real-space measurements in UCSF Chimera. Helical refinement using both C1 and D1 symmetries was then carried out, producing final maps with a helical twist of 172.4° and a rise of 48.47 Å, reaching an overall resolution of 4.2 Å (Supplementary Figs. 10 and 11).

To improve local detail, focused refinement on the central regulatory domain stalk was performed using a soft mask, increasing the resolution locally to 4.0 Å. Additionally, separate helical refinement focusing on two catalytic domains and adjacent regulatory domains was conducted to highlight the linker motif connecting CDs and RDs, achieving a resolution of approximately 4.8 Å (Supplementary Figs. 10 and 11). In parallel, an SPA-like approach was employed. Duplicate particles were removed from the helical dataset (filtered by a minimum separation threshold), followed by two further rounds of 2D classification and one ab-initio reconstruction. This process yielded 106,308 particles, which were subjected to NU and local refinements to better resolve one catalytic domain and two regulatory domains, clarifying inter-domain interfaces (Supplementary Fig. 12). Finally, an overall filament-focused local refinement without explicit helical symmetry (C1 symmetry imposed) was performed to validate structural consistency. No significant differences were observed when comparing maps generated using C1 and D1 symmetries, confirming the stability and homogeneity of the SAO-*cis*-basal CBS conformation.

#### The SAM-*allo*-activated stacked CBS (dataset 4)

For the SAM-*allo*-activated stacked CBS (dataset 4), two batches of movies were collected: one consisting of 3910 movies used primarily to optimize grid preparation and enhance the number of non-aggregated assemblies, and a second, larger batch of 8670 movies. After CTF correction and initial screening, 11,510 high-quality movies were selected from a total of 12,580 movies recorded in EER format. Movies were collected at a total electron dose of 42 e⁻/Å² and a pixel size of 0.83 Å.

Filament particles were initially picked using cryoSPARC’s filament tracer tool, testing separation distances of 30, 40, 60, and 100 Å, ultimately choosing 100 Å separation for the best capture of the stacked filament architecture. An initial set of ~620,000 particles was extracted and subjected to multiple rounds of 2D classification to remove low-quality picks and aggregates, resulting in a refined set of 120,000 particles. Ab-initio reconstructions with *K* = 4 classes were performed, and the two best classes were merged for initial helical reconstructions. Helical parameters, including twist and rise, were determined using cryoSPARC tools, real-space marker measurements in UCSF Chimera, and analysis of power spectra of elongated filaments (Supplementary Fig. 14).

Several iterative rounds of helical refinement and reconstruction were conducted, progressively restricting the alignment resolution to 12, 10, 8, and 6 Å to enhance overall map quality and minimize misalignment artifacts. C1, D1, and C2 helical symmetries were systematically tested to improve map fidelity and to verify that no artifacts were introduced by symmetry imposition (Supplementary Figs. 15 and 16). The subsequent two rounds of 3D classification (*K* = 3), followed by additional 2D classification to exclude suboptimal segments, resulted in a final refined subset of 33,700 particles for helical reconstruction. The RDs stalks and outer CDs displayed an overall right-handed twist with optimized parameters of 175.6° and a rise of 48 Å, while the central stacked CDs region showed a distinct structural arrangement.

In parallel, an SPA-like analysis was also performed. From the initial extraction, ~1.25 million particles were subjected to multiple rounds of 2D classification. A final subset of 41,174 particles, corresponding to stacked filament segments, was selected after duplicate removal using an 80 Å separation cutoff. These particles were refined through helical refinement, NU refinement, and local refinement to validate map consistency and to resolve orientations of the outer CDs observed in 2D classes. Final reconstructions using this approach achieved resolutions between 7 and 10 Å, providing improved visualization of the outer CDs and RDs stalks (Supplementary Fig. 17).

Focused classification and refinement with masks centered on the core of the filament were performed in both helical and SPA-like frameworks. Further NU and local refinements were used to attempt to resolve the diffuse central CD density and to trace the linker motifs connecting the RDs to the CDs in the center of the stack. Despite extensive efforts, the resolution of the central CD region did not improve substantially; however, these refinements clarified which RDs contribute to the central CD assembly. From the ~8 Å maps, we concluded that the motif organization and CD dimer assembly in the central core of the SAM-*allo*-activated stacked CBS filament are distinct from those in the *trans*-basal, *cis*-basal, and outer stacked CDs.

In addition, similarly to the SAO-*cis*-basal CBS dataset, we reconstructed a SAM-bound *cis*-basal CBS conformer. Using helical reconstruction, this structure reached an overall resolution of 4 Å, which was further improved to 3.8 Å through NU, local, and SPA-like refinements (Supplementary Fig. 18). We also observed various fragmented assemblies, including isolated RD filaments, partial CDs not arranged into filaments, and incomplete tetrameric structures, highlighting the structural heterogeneity induced by SAM binding (Supplementary Fig. 13).

### Model building, refinement, and validation

For the *trans*-basal CBS and Ser-*trans*-basal CBS (datasets 1 and 2) structures, initial models were generated by docking the crystal structure of CBSΔ516–525 (PDB 4COO) into the cryo-EM maps using rigid-body fitting in UCSF Chimera/ChimeraX^55,56^. The missing loop region (residues 513–527) that was absent in the template model and is critical for oligomerization, was manually rebuilt in Coot based on visible cryo-EM density and known secondary structure predictions^59^. The catalytic core in these reconstructions exhibited clear density for both cofactors, supporting one heme per CBS monomer with the expected axial coordination by C52 and H65, and one PLP cofactor was positioned as in the template structures. In the substrate-free state, the PLP-Lys internal aldimine was modeled as the modified residue LLP, reflecting the covalent Schiff-base linkage to K119 and was iteratively refined in Phenix and Coot.

Multiple copies of the refined CBS protomer were then placed into the helical assemblies guided by the determined symmetry parameters. Initial fits were followed by iterative rounds of real-space refinement using Phenix to optimize geometry, improve map-to-model correlation, and minimize steric clashes^60,61^. Flexible fitting steps were performed using Coot, allowing local conformational adjustments to better accommodate subtle variations within the filament interfaces^59^.

For the catalytic intermediates (Ser-*trans*-basal CBS): external-aldimine and amino-acrylate assignments and covalent geometry were guided by the high-resolution reference structures from *Drosophila* CBS (e.g., aminoacrylate: PDB 3PC3; external-aldimine-like intermediate: PDB 3PC4). The cryo-EM densities in our substrate-bound datasets were of sufficiently high resolution to distinguish the different PLP adduct states, enabling identification of the external aldimine and aminoacrylate intermediates. These modified PLP ligands were then manually adjusted in Coot to match the density and refined in Phenix with covalent and geometric restraints, with particular attention to bond geometry, planarity, and local coordination around the PLP adduct.

For the SAO-*cis*-basal CBS structure (dataset 3), models of the RDs and CDs were fitted into focused maps to account for relative flexibility between the central RD stalk and CDs. The CBS module containing the RD in *cis* conformation was modeled based on the SAM-bound CBSΔ516–525 E201S structure (PDB 4UUU) as a template. The heme and PLP cofactors in the catalytic core were positioned using the template-derived placement and retained under Phenix restraints, with coordination/geometry verified in Coot. The SAO ligand was generated from the standard PDB chemical library (SMILES-based coordinates), docked into density at the S2 site, and refined iteratively in Coot and Phenix using appropriate stereochemical restraints.

For the SAM-bound *allo*-activated stacked assembly (dataset 4), modeling was performed in a domain-by-domain manner because of the anisotropic resolution and flexibility. The RD models derived from the relatively higher-resolution *cis*-basal structures were rigid-body fitted into two central stalk densities in Chimera and adjusted in Coot. Peripheral catalytic domains were docked using CD models of the *cis*-basal conformer into focused reconstructions where density allowed reliable placement^55,56,59^. In the stacked core, the local resolution remained insufficient to refine active-site geometry at atomic detail. Therefore, the model interpretation of the central catalytic interfaces was limited to rigid-body placement and qualitative agreement of the backbone chain with the density. The inter-filament contacts defining the *allo*-dimer arrangement were assessed by visual inspection in ChimeraX, and overall model consistency was evaluated against the global map^56^.

In summary, the ligands were handled according to the confidence provided by the maps. Heme and PLP were retained in all reconstructions where the CD was resolved sufficiently to support cofactor placement and coordination geometry (i.e., datasets 1–3 and peripheral CDs in dataset 4). In contrast, ligands and PLP-derived adducts that change chemically during catalysis required additional modeling as described earlier. All the models were subjected to comprehensive validation with MolProbity to assess overall stereochemistry, hydrogen bonding networks, and sidechain rotameric states^62^. The refined models displayed acceptable overall geometry with minimal outliers and were consistent with the cryo-EM densities across the entire filament structures.

### Preparation of CBS-expressing cell lines

The HEK293A cells (Invitrogen# R70507) lacking CBS (CBS knockout) were generated by the CRISPR-Cas9 approach as detailed elsewhere^24^. Stable cell lines expressing various human CBS constructs (ISO1, ISO2, Δ516–525, and CBS45) were prepared as described previously^24^. Briefly, codon-optimized human CBS WT (ISO1, Uniprot# P35520-1) and isoform 2 (ISO2, Uniprot# P35520-2) carrying myc-tag and 6×His-tag at their N- and C-termini, respectively, were synthesized by Genscript and subcloned using pLentiCMVBlast-empty (w263-1) plasmid (cat# 17486, Addgene) directionally into SalI and XbaI restriction endonuclease sites. The CBSΔ516–525 and CBS45 (CBSΔ414–551) were prepared by site-directed mutagenesis by Genscript using the CBS WT (ISO1) plasmid as a template. All constructs were verified by DNA sequencing (Genscript). Supplied plasmids were used for the generation of lentiviral particles, which were used to transduce HEK293A CBS KO in the presence of 6 μg/ml protamine sulfate. Blasticidin S (45 μg/ml) was added to the culture 72 h after transduction to select for cells carrying the CBS construct.

### Determination of CBS half-life

Cellular turnover of overexpressed CBS constructs was determined by using nonradioactive method based on the incorporation of L-azidohomoalanine (AHA), a biorthogonal analog of Met, as described previously^24^ with a few modifications. The cells were grown to ∼80% confluency in a six-well plate. The cells were then washed with Met-deficient DMEM containing 10% dialyzed FBS, 2 mM GlutaMax, 1 mM pyruvate and 200 μM cystine hydrochloride, followed by live-labeling for 2 h (pulse) in a Met-deficient DMEM supplemented with 75 μM AHA. The AHA pulse was terminated by removing the labeling medium and washing with a complete standard medium, then incubated in the same medium for up to 24 h at 37 °C. Immediately after the pulse (0 h) and at designated timepoints during the chase period (2, 4, 6, 9, 12, and 24 h), the cells were harvested, lysed and the lysates analyzed as described previously^24^.

The half-life of endogenous CBS under different metabolic conditions was determined similarly as described above, with the following modifications. The HEP3B cells were seeded at 30% confluency and cultured in 100 mm dishes for 48 h. After the 4 h labeling pulse with 100 μM AHA, the AHA-containing Met-deficient DMEM medium was replaced with the complete low-glucose DMEM medium (control) or the same medium supplemented with 1 mM Met or 10 µM AGI-43192. Cells were cultured for up to 24 h after the AHA pulse and harvested at designated timepoints (0, 2, 6, 9, and 24 h).

### Confocal imaging

The cells were seeded into an 8-well cell culture chamber glass slide with a removable frame at a density of 80,000 cells/well. After overnight incubation, the cells were washed, fixed with 4% PFA for 15 min at room temperature (RT), washed three times, then permeabilized in PBS containing 0.2% Triton X-100 for 10 min at RT and washed three times before being incubated in 10% goat serum for 10 min at RT. Cells were then washed and incubated with anti-CBS monoclonal antibody (CST# 14782) at a concentration of 1:500 overnight at 4 °C. The following day, cells were washed three times with PBS and incubated with the Alexa Fluor Plus 568-conjugated anti-rabbit secondary antibody (Invitrogen# A-11011) at a 1:1000 dilution for 1 h at RT. Nuclear stain DAPI (5 µg/ml) was added for the last 5 min of the incubation. Cells were then washed three times with PBS, and the cover slip was mounted using Prolong Gold antifade reagent. Slides were dried overnight in the dark and stored at −20 °C until further use. Leica TCS SP5 confocal microscope equipped with a motorized conventional Galvo stage was used for the visualization of cells in a Z-stack. Several optical sections were acquired along the Z-axis at 1 μm step size using ×40 magnification. Series of images were captured using an image format of 1024 × 1024 pixels and 200 Hz scan speed. DAPI was excited using a 405 nm UV laser, while CBS was visualized using a 561 nm DPSS laser. Fluorescence emission was recorded at 419–474 nm (DAPI) and 584–683 nm (Alexa Fluor 568) in a sequential mode. In each chamber, images from two randomly selected regions were acquired, and all experiments were independently performed five times. This resulted in 10 regions of interest for each group with more than 100 cells per group. Image reconstruction was completed from a series of Z-stack images using Imaris v10.0.1 software. Identical parameters for fluorescence intensity settings were applied for each group in all four investigated groups. Images are shown as a single layer from the middle part of the stack and as a 3D reconstructed z-stack in series of all images. The corrected total CBS fluorescence was quantified by Fiji/ImageJ software using the CTCF formula: CTCF = integrated density − (area of selected cell × mean fluorescence of a background). Results are shown as mean ± standard errors of the mean (SEM), from five independent experiments. Graphpad Prism 8 software was used for statistical analysis using one-way ANOVA followed by Tukey’s post-hoc test. The value of *p* < 0.05 indicated statistical significance, denoted by an asterisk (*).

### Protein electrophoresis and Western blot analysis

Cell lysates were mixed with either 2× Novex Tris-glycine Native sample buffer for native electrophoresis or 4× Bolt LDS sample buffer containing a sample reducing agent for reducing denaturing electrophoresis. Denaturation of protein samples was achieved by heating at 95 °C for 10 min. Native proteins were resolved in NativePAGE 4–16% Bis-Tris gels in 1× NativePAGE running buffer at 4 °C, while reduced, denatured proteins were separated in NuPAGE 4–12% Bis-Tris gels in 1×NuPAGE MES SDS running buffer. Subsequently, proteins were transferred onto the PVDF membrane using an iBlot 2 gel transfer device (Invitrogen). Membranes were blocked in 5% non-fat milk in TBST (Tris-buffered saline supplemented with 0.1% Tween 20) for 1 h at RT, followed by incubation with the primary antibody while gently agitating either for 1 h at RT or overnight at 4 °C. Primary antibodies were diluted in TBST containing 5% BSA anti-CBS (1:2000, CST# 14782) or anti-beta-actin (1:5000; Sigma# A1978). After washing three times with TBST for 5 min, blots were incubated with anti-rabbit or anti-mouse IgG HRP-conjugated secondary antibody (1:5000; CST# 7074 and 7076, respectively) in TBST containing 5% non-fat milk for 1 h at RT. After washing with TBST for 5 min twice and TBS for 5 min twice, the proteins were visualized with Radiance Plus chemiluminescence substrate using the Azure Imaging System 300. Captured images were analyzed using Fiji/ImageJ and plotted by Graphpad Prism.

### CBS activity assay

CBS activities in native gels and cell lysates were determined as described previously^24^. Briefly, cell lysates (10 µg/lane) were resolved on native PAGE, and the gel was developed in a staining solution (100 mM Tris.HCl pH 8.0, 20 mM cysteine, 50 mM beta-mercaptoethanol, 100 μM PLP, 200 μM lead acetate) in the absence or presence of 200 μM SAM. The gels were gently shaking at 37 °C until the active CBS bands became apparent. The reaction was terminated after the same amount of time for both conditions by placing the gels into 7% acetic acid. The gels were scanned, and the CBS activity was quantified by densitometry using ImageJ.

CBS activity in the lysates or purified proteins was determined using H_2_S-specific fluorescent probe 7-azido-4-methylcoumarin (AzMC) as described previously^63^. Briefly, the reaction (200 μl total volume) containing 50 mM Tris HCl pH 8.6, 5 μM PLP, 10 μM AzMC, 10 μg of cell lysate or 1 µg of purified CBS was initiated after 10 min equilibration at 37 °C with a mixture of substrates yielding final concentrations of 2 mM cysteine and 500 μM homocysteine. The assays containing variable final concentrations of SAM were followed for 90 min at 37 °C, recording fluorescent intensities in 5 min intervals (excitation 365 nm, emission 450 nm) Spectramax M5 microplate reader. All assays were done in three replicates, and the data were analyzed using Microsoft Excel and Graphpad Prism software.

The CBS thermal activation assay was essentially carried out as described previously^26,27^. Briefly, the purified enzymes were diluted to a final concentration of 0.1 mg/ml in 20 mM HEPES pH 7.4, 200 mM NaCl, 50 µM PLP, and gradually heated in 0.2 ml thin-walled PCR tubes using the Mastercycler gradient PCR thermal cycler (Eppendorf). The temperature was raised from 37 to 60 °C in 0.5 °C increments with a 1 min incubation at each temperature. At designated temperatures, small aliquots were collected into separate tubes and kept on ice until the last aliquot was taken. The CBS activity was subsequently determined by colorimetric methylene blue CBS activity assay^7,64^.

To investigate the reversibility of CBS allosteric activation by SAM, the full-length human CBS (10 mg/ml) was incubated at room temperature for 10 min in 100 mM Tris HCl, pH 8.6, in the absence and presence of 200 µM SAM. Subsequently, standard CBS activity assay using AzMC was performed as described above in the increasing or decreasing final concentration of SAM equal to 200, 60, 20, 6, 2, 0.6, 0.2, 0.06, 0.02, 0.006, 0.002, and 0 µM. The mixtures were incubated at 37 °C for 10 min prior to the addition of substrates (0.5 mM Hcy and 2 mM Cys final), which started the reaction.

### Dissociation of CBS filaments

Fifty µl slurry of Ni^2+^-chelated magnetic beads (Genscript) was washed 3× with PBS containing 0.1% Tween20. Subsequently, 300 µl of 500 µg/ml full-length human CBS containing a permanent C-terminal 6×His tag was mixed with the washed beads and allowed to bind while gently mixed at room temperature for 1 h. Afterwards, the unbound portion was removed by washing the beads 3× with PBS containing 0.1% Tween20, followed by an additional wash with PBS. The suspension was then divided into two tubes, and the beads were collected at the tube wall using a magnet. The beads were resuspended in 300 µl PBS, pulled by the magnet, and 30 µl aliquot of the supernatant was removed to serve as the initial time zero sample. Then, 30 µl of PBS or 5 mM SAM (to achieve 0 or 500 µM SAM final concentration) were mixed with the beads, and the supernatants were collected after 1, 2, 3, 5, 10, 20, and 30 min, ensuring that the beads are pulled by the magnet to the wall of the tube and not disturbed. After the final time point, the beads were eluted in 1× LDS Sample buffer. All collected samples were subsequently analyzed using SDS-PAGE and Western Blot (WB) to assess the kinetics of CBS filament dissociation.

### Thermal shift assay

Purified CBS proteins were diluted to 0.3 mg/ml in assay buffer (25 mM HEPES, pH 7.4). An original stock of SYPRO Orange dye (ThermoFisherScientific) was 5000× diluted in assay buffer, yielding a 50× working solution. For each reaction, 8 µl of CBS solution was mixed with 10 µl of either assay buffer or 500 µM SAM and 2 µl of the 50× SYPRO Orange working solution. The samples in the PCR plate were sealed and subjected to a thermal ramp from 30 to 80 °C at a rate of 0.5 °C/min in a QuantStudio5 RT-PCR machine (ThermoFisherScientific). Fluorescence intensities were measured using the TAMRA channel at 0.5 °C intervals with a 20-s hold prior to each reading. The melting temperatures (*T*_m_) were determined from the inflection points of the fluorescence-versus-temperature curves.

### Statistical analysis

Data are presented as mean ± SEM unless otherwise specified. Statistical analyses were carried out using Prism software (GraphPad). Comparisons between two groups were performed using an unpaired, two-tailed Student’s *t* test. For experiments involving more than two groups, one-way ANOVA followed by Dunnett’s or Tukey’s post-hoc test was applied to determine statistical significance. The value of *p* < 0.05 was considered significant and designated by the asterisk in the figures. The number of biological or technical replicates (*n*) varied and is indicated in the figure legends.

### Reporting summary

Further information on research design is available in the Nature Portfolio Reporting Summary linked to this article.

## Supplementary information

Supplementary Information

Description of Additional Supplementary Files

Supplementary Movie 1

Supplementary Movie 2

Reporting Summary

Transparent Peer Review File

## Source data

Source Data

## Data Availability

All cryo-EM density maps and corresponding atomic coordinates generated in this study have been deposited in the Electron Microscopy Data Bank (EMDB) and Protein Data Bank (PDB). The *trans*-basal CBS filaments in the absence of substrates or allosteric ligands are available under accession codes EMD-55115/PDB 9SQQ, EMD-55117/PDB 9SQU, and EMD-55105/PDB 9SQ0. Serine-bound *trans*-basal CBS is deposited as EMD-55037/PDB 9SML, EMD-54904/PDB 9SHM, EMD-54905/PDB 9SHN, and EMD-54925/PDB 9SI8. The SAO-bound *cis*-basal CBS filaments are deposited as EMD-55095/PDB 9SPT, EMD-55097/PDB 9SPV, EMD-55099/PDB 9SPW, and EMD-55102. The SAM-bound *allo*-activated stacked CBS filaments are deposited as EMD-55128/PDB 9SR3, EMD-55130/PDB 9SR4 and PDB 9SR7. In addition, the SAM-bound *cis*-basal CBS filament is deposited as EMD-55132/PDB 9SR6 and EMD-55133. A complete list of all datasets and accession codes is provided in Supplementary table 1. Other structures referenced in this article are indicated, including 4COO, 3PC3, 3PC4, and 4UUU. Source data are provided within this paper. Source data are provided with this paper.

## References

[1] Stipanuk, M. H. Sulfur amino acid metabolism: pathways for production and removal of homocysteine and cysteine. *Annu. Rev. Nutr.* **24**, 539–577 (2004). 15189131 10.1146/annurev.nutr.24.012003.132418

[2] Brosnan, J. T. & Brosnan, M. E. The sulfur-containing amino acids: an overview. *J. Nutr.* **136**, 1636S–1640S (2006). 16702333 10.1093/jn/136.6.1636S

[3] Majtan, T., Kozich, V. & Kruger, W. D. Recent therapeutic approaches to cystathionine beta-synthase-deficient homocystinuria. *Br. J. Pharmacol.* **180**, 264–278 (2023). 36417581 10.1111/bph.15991 PMC9822868

[4] Cirino, G., Szabo, C. & Papapetropoulos, A. Physiological roles of hydrogen sulfide in mammalian cells, tissues, and organs. *Physiol. Rev.* **103**, 31–276 (2023). 35435014 10.1152/physrev.00028.2021

[5] Zuhra, K., Augsburger, F., Majtan, T. & Szabo, C. Cystathionine-beta-synthase: molecular regulation and pharmacological inhibition. *Biomolecules* **10**, 697 (2020). 32365821 10.3390/biom10050697 PMC7277093

[6] Mudd, S. H. et al. The natural history of homocystinuria due to cystathionine β-synthase deficiency. *Am. J. Hum. Genet.* **37**, 1–31 (1985). 3872065 PMC1684548

[7] Majtan, T. et al. Biogenesis of hydrogen sulfide and thioethers by cystathionine beta-synthase. *Antioxid. Redox Signal.* **28**, 311–323 (2018). 28874062 10.1089/ars.2017.7009

[8] Szabo, C. et al. Tumor-derived hydrogen sulfide, produced by cystathionine-beta-synthase, stimulates bioenergetics, cell proliferation, and angiogenesis in colon cancer. *Proc. Natl. Acad. Sci. USA* **110**, 12474–12479 (2013). 23836652 10.1073/pnas.1306241110 PMC3725060

[9] Erdelyi, K. et al. Reprogrammed transsulfuration promotes basal-like breast tumor progression via realigning cellular cysteine persulfidation. *Proc. Natl. Acad. Sci. USA* **118**, e2100050118 (2021). 34737229 10.1073/pnas.2100050118 PMC8609449

[10] Jiang, Q. Y. et al. Cystathionine beta-synthase regulates the proliferation, migration, and invasion of thyroid carcinoma cells. *Oxid. Med. Cell. Longev.* **2022**, 8678363 (2022). 35795862 10.1155/2022/8678363 PMC9252770

[11] Panagaki, T., Randi, E. B., Augsburger, F. & Szabo, C. Overproduction of H2S, generated by CBS, inhibits mitochondrial complex IV and suppresses oxidative phosphorylation in Down syndrome. *Proc. Natl. Acad. Sci. USA* **116**, 18769–18771 (2019). 31481613 10.1073/pnas.1911895116 PMC6754544

[12] Szabo, C. The re-emerging pathophysiological role of the cystathionine-beta-synthase- hydrogen sulfide system in Down syndrome. *FEBS J.* **287**, 3150–3160 (2020). 31955501 10.1111/febs.15214

[13] Ascencao, K. & Szabo, C. Emerging roles of cystathionine beta-synthase in various forms of cancer. *Redox Biol.* **53**, 102331 (2022). 10.1016/j.redox.2022.102331 PMC9168780 35618601

[14] Meier, M., Janosik, M., Kery, V., Kraus, J. P. & Burkhard, P. Structure of human cystathionine beta-synthase: a unique pyridoxal 5’-phosphate-dependent heme protein. *EMBO J.* **20**, 3910–3916 (2001). 11483494 10.1093/emboj/20.15.3910 PMC149156

[15] Taoka, S. et al. Human cystathionine beta-synthase is a heme sensor protein. Evidence that the redox sensor is heme and not the vicinal cysteines in the CXXC motif seen in the crystal structure of the truncated enzyme. *Biochemistry* **41**, 10454–10461 (2002). 12173932 10.1021/bi026052d

[16] Ereno-Orbea, J., Majtan, T., Oyenarte, I., Kraus, J. P. & Martinez-Cruz, L. A. Structural basis of regulation and oligomerization of human cystathionine beta-synthase, the central enzyme of transsulfuration. *Proc. Natl. Acad. Sci. USA* **110**, E3790–E3799 (2013). 24043838 10.1073/pnas.1313683110 PMC3791738

[17] Ereno-Orbea, J., Majtan, T., Oyenarte, I., Kraus, J. P. & Martinez-Cruz, L. A. Structural insight into the molecular mechanism of allosteric activation of human cystathionine beta-synthase by S-adenosylmethionine. *Proc. Natl. Acad. Sci. USA* **111**, E3845–E3852 (2014). 25197074 10.1073/pnas.1414545111 PMC4169959

[18] McCorvie, T. J. et al. Inter-domain communication of human cystathionine beta-synthase: structural basis of S-adenosyl-L-methionine activation. *J. Biol. Chem.* **289**, 36018–36030 (2014). 25336647 10.1074/jbc.M114.610782 PMC4276868

[19] Kraus, J. P., Packman, S., Fowler, B. & Rosenberg, L. E. Purification and properties of cystathionine β-synthase from human liver. *J. Biol. Chem.* **253**, 6523–6528 (1978). 681363

[20] McCorvie, T. J. et al. Architecture and regulation of filamentous human cystathionine beta-synthase. *Nat. Commun.* **15**, 2931 (2024). 38575566 10.1038/s41467-024-46864-x PMC10995199

[21] Sen, S. & Banerjee, R. A pathogenic linked mutation in the catalytic core of human cystathionine beta-synthase disrupts allosteric regulation and allows kinetic characterization of a full-length dimer. *Biochemistry* **46**, 4110–4116 (2007). 17352495 10.1021/bi602617f PMC3204387

[22] Lawrence, S. H. & Jaffe, E. K. Expanding the concepts in protein structure-function relationships and enzyme kinetics: teaching using morpheeins. *Biochem. Mol. Biol. Educ.* **36**, 274–283 (2008). 19578473 10.1002/bmb.20211 PMC2575429

[23] Park, C. K. & Horton, N. C. Structures, functions, and mechanisms of filament forming enzymes: a renaissance of enzyme filamentation. *Biophys. Rev.* **11**, 927–994 (2019). 31734826 10.1007/s12551-019-00602-6 PMC6874960

[24] Mijatovic, E., Ascencao, K., Szabo, C. & Majtan, T. Cellular turnover and degradation of the most common missense cystathionine beta-synthase variants causing homocystinuria. *Protein Sci.* **33**, e5123 (2024). 39041895 10.1002/pro.5123 PMC11264351

[25] Janosik, M., Kery, V., Gaustadnes, M., Maclean, K. N. & Kraus, J. P. Regulation of human cystathionine beta-synthase by S-adenosyl-L-methionine: evidence for two catalytically active conformations involving an autoinhibitory domain in the C-terminal region. *Biochemistry* **40**, 10625–10633 (2001). 11524006 10.1021/bi010711p

[26] Majtan, T., Liu, L., Carpenter, J. F. & Kraus, J. P. Rescue of cystathionine beta-synthase (CBS) mutants with chemical chaperones: purification and characterization of eight CBS mutant enzymes. *J. Biol. Chem.* **285**, 15866–15873 (2010). 20308073 10.1074/jbc.M110.107722 PMC2871454

[27] Majtan, T. & Kraus, J. P. Folding and activity of mutant cystathionine beta-synthase depends on the position and nature of the purification tag: characterization of the R266K CBS mutant. *Protein Expr. Purif.* **82**, 317–324 (2012). 22333527 10.1016/j.pep.2012.01.019 PMC3319291

[28] Singh, S., Padovani, D., Leslie, R. A., Chiku, T. & Banerjee, R. Relative contributions of cystathionine beta-synthase and gamma-cystathionase to H2S biogenesis via alternative trans-sulfuration reactions. *J. Biol. Chem.* **284**, 22457–22466 (2009). 19531479 10.1074/jbc.M109.010868 PMC2755967

[29] Majtan, T., Pey, A. L. & Kraus, J. P. Kinetic stability of cystathionine beta-synthase can be modulated by structural analogs of S-adenosylmethionine: potential approach to pharmacological chaperone therapy for homocystinuria. *Biochimie* **126**, 6–13 (2016). 26805382 10.1016/j.biochi.2016.01.009

[30] Frank, N., Kent, J. O., Meier, M. & Kraus, J. P. Purification and characterization of the wild type and truncated human cystathionine beta-synthase enzymes expressed in *E. coli*. *Arch. Biochem. Biophys.* **470**, 64–72 (2008). 18060852 10.1016/j.abb.2007.11.006 PMC3365551

[31] Kashiwamata, S., Kotake, Y. & Greenberg, D. M. Studies of cystathionine synthase of rat liver: dissociation into two components by sodium dodecyl sulfate disc electrophoresis. *Biochim. Biophys. Acta* **212**, 501–503 (1970). 5466144 10.1016/0005-2744(70)90256-1

[32] Skovby, F., Kraus, J. P. & Rosenberg, L. E. Biosynthesis and proteolytic activation of cystathionine b-synthase in rat liver. *J. Biol. Chem.* **259**, 588–593 (1984). 6706953

[33] Bukovska, G., Kery, V. & Kraus, J. P. Expression of human cystathionine beta-synthase in *Escherichia coli*: purification and characterization. *Protein Expr. Purif.* **5**, 442–448 (1994). 7827502 10.1006/prep.1994.1063

[34] Jaffe, E. K. Morpheeins–a new structural paradigm for allosteric regulation. *Trends Biochem. Sci.* **30**, 490–497 (2005). 16023348 10.1016/j.tibs.2005.07.003

[35] Ma, B. & Nussinov, R. Structured crowding and its effects on enzyme catalysis. *Top. Curr. Chem.* **337**, 123–137 (2013). 23571857 10.1007/128_2012_316 PMC6361544

[36] Pey, A. L., Martinez-Cruz, L. A., Kraus, J. P. & Majtan, T. Oligomeric status of human cystathionine beta-synthase modulates AdoMet binding. *FEBS Lett.* **590**, 4461–4471 (2016). 27861796 10.1002/1873-3468.12488

[37] Pey, A. L., Majtan, T., Sanchez-Ruiz, J. M. & Kraus, J. P. Human cystathionine beta-synthase (CBS) contains two classes of binding sites for S-adenosylmethionine (SAM): complex regulation of CBS activity and stability by SAM. *Biochem. J.* **449**, 109–121 (2013). 22985361 10.1042/BJ20120731

[38] Koutmos, M., Kabil, O., Smith, J. L. & Banerjee, R. Structural basis for substrate activation and regulation by cystathionine beta-synthase (CBS) domains in cystathionine {beta}-synthase. *Proc. Natl. Acad. Sci. USA* **107**, 20958–20963 (2010). 21081698 10.1073/pnas.1011448107 PMC3000283

[39] Lee, H. O. et al. Impact of primary sequence changes on the self-association properties of mammalian cystathionine beta-synthase enzymes. *Protein Sci.* **33**, e5223 (2024). 39548832 10.1002/pro.5223 PMC11568414

[40] Lynch, E. M. & Kollman, J. M. Coupled structural transitions enable highly cooperative regulation of human CTPS2 filaments. *Nat. Struct. Mol. Biol.* **27**, 42–48 (2020). 31873303 10.1038/s41594-019-0352-5 PMC6954954

[41] Hvorecny, K. L., Hargett, K., Quispe, J. D. & Kollman, J. M. Human PRPS1 filaments stabilize allosteric sites to regulate activity. *Nat. Struct. Mol. Biol.* **30**, 391–402 (2023). 36747094 10.1038/s41594-023-00921-z PMC10033377

[42] Chang, C. C. et al. Molecular crowding facilitates bundling of IMPDH polymers and cytoophidium formation. *Cell. Mol. Life Sci.* **79**, 420 (2022). 35833994 10.1007/s00018-022-04448-2 PMC11072341

[43] Greene, E. et al. Product-stabilized filamentation by human glutamine synthetase allosterically tunes metabolic activity. *eLife* **14**, RP108336 10.7554/eLife.108336.1 (2025).

[44] Janosik, M. et al. Impaired heme binding and aggregation of mutant cystathionine beta-synthase subunits in homocystinuria. *Am. J. Hum. Genet.* **68**, 1506–1513 (2001). 11359213 10.1086/320597 PMC1226138

[45] Kozich, V. et al. Cystathionine beta-synthase mutations: effect of mutation topology on folding and activity. *Hum. Mutat.* **31**, 809–819 (2010). 20506325 10.1002/humu.21273 PMC2966864

[46] Collard, R. & Majtan, T. Genetic and pharmacological modulation of cellular proteostasis leads to partial functional rescue of homocystinuria-causing cystathionine-beta synthase variants. *Mol. Cell. Biol.* **43**, 664–674 (2023). 38051092 10.1080/10985549.2023.2284147 PMC10761163

[47] Majtan, T., Mijatovic, E. & Petrosino, M. Understanding the impact of mutations in the cystathionine beta-synthase gene: towards novel therapeutics for homocystinuria. *Mol. Cell. Biol*. **45**, 327–342 (2025). 10.1080/10985549.2025.2511338 40495464

[48] Majtan, T., Pey, A. L., Ereno-Orbea, J., Martinez-Cruz, L. A. & Kraus, J. P. Targeting cystathionine beta-synthase misfolding in homocystinuria by small ligands: state of the art and future directions. *Curr. Drug Targets* **17**, 1455–1470 (2016). 26931358 10.2174/1389450117666160302094910

[49] Philipp, T. M. et al. Structure-activity relationship of S-adenosylmethionine analogs as pharmacological chaperones for cystathionine beta-synthase-deficient homocystinuria. *Int. J. Biol. Macromol.* **339**, 150016 (2026). 41478486 10.1016/j.ijbiomac.2025.150016

[50] Derry, W. B., Wilson, L. & Jordan, M. A. Substoichiometric binding of taxol suppresses microtubule dynamics. *Biochemistry* **34**, 2203–2211 (1995). 7857932 10.1021/bi00007a014

[51] Lawrence, S. H. et al. Shape shifting leads to small-molecule allosteric drug discovery. *Chem. Biol.* **15**, 586–596 (2008). 18559269 10.1016/j.chembiol.2008.04.012 PMC2703447

[52] Szabo, C. & Papapetropoulos, A. Hydrogen sulphide and angiogenesis: mechanisms and applications. *Br. J. Pharmacol.* **164**, 853–865 (2011). 21198548 10.1111/j.1476-5381.2010.01191.x PMC3195910

[53] Punjani, A., Rubinstein, J. L., Fleet, D. J. & Brubaker, M. A. cryoSPARC: algorithms for rapid unsupervised cryo-EM structure determination. *Nat. Methods* **14**, 290–296 (2017). 28165473 10.1038/nmeth.4169

[54] Rohou, A. & Grigorieff, N. CTFFIND4: fast and accurate defocus estimation from electron micrographs. *J. Struct. Biol.* **192**, 216–221 (2015). 26278980 10.1016/j.jsb.2015.08.008 PMC6760662

[55] Pettersen, E. F. et al. UCSF Chimera–a visualization system for exploratory research and analysis. *J. Comput. Chem.* **25**, 1605–1612 (2004). 15264254 10.1002/jcc.20084

[56] Pettersen, E. F. et al. UCSF ChimeraX: structure visualization for researchers, educators, and developers. *Protein Sci.* **30**, 70–82 (2021). 32881101 10.1002/pro.3943 PMC7737788

[57] Punjani, A., Zhang, H. & Fleet, D. J. Non-uniform refinement: adaptive regularization improves single-particle cryo-EM reconstruction. *Nat. Methods* **17**, 1214–1221 (2020). 33257830 10.1038/s41592-020-00990-8

[58] Laskowski, R. A. & Swindells, M. B. LigPlot+: multiple ligand-protein interaction diagrams for drug discovery. *J. Chem. Inf. Model.* **51**, 2778–2786 (2011). 21919503 10.1021/ci200227u

[59] Emsley, P., Lohkamp, B., Scott, W. G. & Cowtan, K. Features and development of Coot. *Acta Crystallogr. D Biol. Crystallogr.* **66**, 486–501 (2010). 20383002 10.1107/S0907444910007493 PMC2852313

[60] Adams, P. D. et al. The Phenix software for automated determination of macromolecular structures. *Methods* **55**, 94–106 (2011). 21821126 10.1016/j.ymeth.2011.07.005 PMC3193589

[61] Afonine, P. V. et al. Real-space refinement in PHENIX for cryo-EM and crystallography. *Acta Crystallogr. D Struct. Biol.* **74**, 531–544 (2018). 29872004 10.1107/S2059798318006551 PMC6096492

[62] Chen, V. B. et al. MolProbity: all-atom structure validation for macromolecular crystallography. *Acta Crystallogr. D Biol. Crystallogr.* **66**, 12–21 (2010). 20057044 10.1107/S0907444909042073 PMC2803126

[63] Thorson, M. K., Majtan, T., Kraus, J. P. & Barrios, A. M. Identification of cystathionine beta-synthase inhibitors using a hydrogen sulfide selective probe. *Angew. Chem. Int. Ed.* **52**, 4641–4644 (2013). 10.1002/anie.201300841 23512751

[64] Philipp, T. M. et al. Mechanism of action and impact of thiol homeostasis on efficacy of an enzyme replacement therapy for classical homocystinuria. *Redox Biol.* **77**, 103383 (2024). 39366068 10.1016/j.redox.2024.103383 PMC11489331

