# Supplementary material for: Structural basis for a filamentous morpheein model of human cystathionine beta-synthase
Source: Nat Commun. 2026 Jun 6;17:7221. doi: 10.1038/s41467-026-73198-7 (PMC13396399; doi:10.1038/s41467-026-73198-7)

Supplementary table 1. Cryo-EM data collection, refinement and validation statistics of the reported human CBS datasets.

	Ligand-free *trans*-basal CBS (Dataset 1)			Serine-bound *trans*-basal CBS (Dataset 2)				SAO-bound *cis*-basal CBS (Dataset 3)				SAM-bound *allo*-activated stacked CBS (Dataset 4)			
Data collection and processing															
Magnification	165,000			270,000				155,000				96,000			
Voltage (Kev)	300			300				300				300			
Electron exposure (e/Å^2)	64			52				50				42			
Defocus range (μm)	-1.1 to -2.5			-1.1 to -2.2				-1.2 to -2.3				-1.2 to -2.5			
Pixel size (Å)	0.72			0.45				0.52				0.83			
Method	Helical	Helical	SPA	Helical	SPA	Helical	SPA	Helical	Helical	SPA	SPA	Helical	SPA	Helical(*cis*)	SPA(*cis*)
EMD ID#	55115	55117	55105	54925	54904	55037	54905	55095	55097	55099	55102	55128	55130	55132	55133
PDB ID#	9SQQ	9SQU	9SQ0	9SI8	9SHM	9SML	9SHN	9SPT	9SPV	9SPW	n/a	9SR3	9SR4	9SR6	n/a
Symmetry	D1	D1	D1	D1	D1	D1	D1	D1	D1	D1	C1	C1	C1	D1	C2
Rise (Å)	49.97	49.88	n/a	49.940	n/a	50.789	n/a	48.50	49.20	n/a	n/a	48.28	n/a	51.07	n/a
Twist (°)	-116.06	-115.16	n/a	-115.312	n/a	-114.984	n/a	-172.47	-172.98	n/a	n/a	-176.08	n/a	-175.49	n/a
Initial particle #	528,877	528,877	528,877	577,766	577,766	577,766	577,766	1.1 million	1.1 million	1.1 million	1.1 million	620,000	500,000	620,000	500,000
Final particle #	335,525	202,702	338,925	187,763	187,763	135,813	135,813	211,125	211,125	106,300	106,300	33,700	27,072	67,000	56,000
Map resolution at FSC (0.143)	3.06	4.71	2.70	2.62	2.08	2.92	2.20	4.86	4.04	4.11	4.86	8.43	7.87	6.76	4.13
Refinement															
Model resolution (Å)	3.15	4.8	2.65	2.5	1.9	2.7	2.1	4.3	4.0	4.0		7.8	7.2	4.0	
FSC threshold	0.143	0.143	0.143	0.143	0.143	0.143	0.143	0.143	0.143	0.143		0.143	0.143	0.143	
Map sharpening B factor (Å^2)	-102.8	-189	-84.7	-98	-68	-125	-72	-450	-86	-91		-500	-500	-120	
Model composition															
Chains	20	32	4	12	4	12	4	42	12	8		76	24	12	
Non-hydrogen atoms	39,540	63,808	7,952	23,880	7,960	23,886	7,962	46,314	8,604	9,870		97,686	25,380	6,972	
Protein residues	5,069	8,112	1,019	3,057	1,019	3,057	1,019	5,970	1,112	1,273		12,645	3,324	864	
Ligands	HEM: 10<br>PLP: 10	HEM: 16	HEM: 2<br>PLP: 2	HEM: 6<br>P1T: 6	HEM: 2<br>P1T: 2	HEM: 6<br>KOU: 6	HEM: 2<br>KOU: 2	HEM: 6<br>PLP: 6<br>SFG: 6	SFG: 4	HEM: 2,<br>PLP: 2<br>SFG: 2		HEM: 26<br>PLP: 26		SAM: 6	
ADP (B factors Å^2)															
Protein	144.44	421.88	117.15	151.43	115.16	192.65	125.45	1132.21	107.99	139.40		1601.98	1190.62	156.17	
Ligand	141.10	257.15	82.13	114.82	61.31	143.85	62.70	908.15	27.50	110.80		1601.98	-	55.07	
Bonds (RMSD)															
Length (Å) (# > 4σ)	0.012	0.003	0.003	0.004	0.005	0.003	0.004	0.003	0.004	0.004		0.004	0.005	0.004	
Angles (°) (# > 4σ)	0.903	0.794	0.762	0.929	0.980	0.679	0.767	0.845	0.918	0.959		0.004	1.173	1.068	
Validation															
MolProbity score	1.86	1.76	1.36	1.22	1.12	1.41	1.24	1.77	1.78	1.77		1.99	1.86	1.71	
Clashscore	10.43	10.64	5.90	3.35	3.26	6.99	3.51	7.69	6.19	7.32		13.86	8.72	4.76	
Poor rotamers (%)	0.02	0.00	0.24	0.00	0.00	0.00	0.12	0.02	0.00	0.19		0.01	0.00	0.00	
Ramachandran plot															
Favored (%)	95.35	96.68	97.83	97.56	98.12	97.89	97.53	94.95	93.15	94.56		95.04	94.24	92.49	
Allowed (%)	4.63	3.31	2.17	2.24	1.88	1.88	2.27	4.95	6.85	5.36		4.92	5.42	7.16	
Disallowed (%)	0.02	0.01	0.00	0.20	0.00	0.23	0.20	0.10	0.00	0.08		0.03	0.34	0.35	

Supplementary figure 1. Topology of secondary structure elements of human CBS WT. Cartoon presentation of the human CBS WT secondary structures within the amino acid sequence. Alpha helices are shown as light blue ovals while beta strands are depicted as light red arrows. Residues of the oligomerization loop 516-525 are in bold.

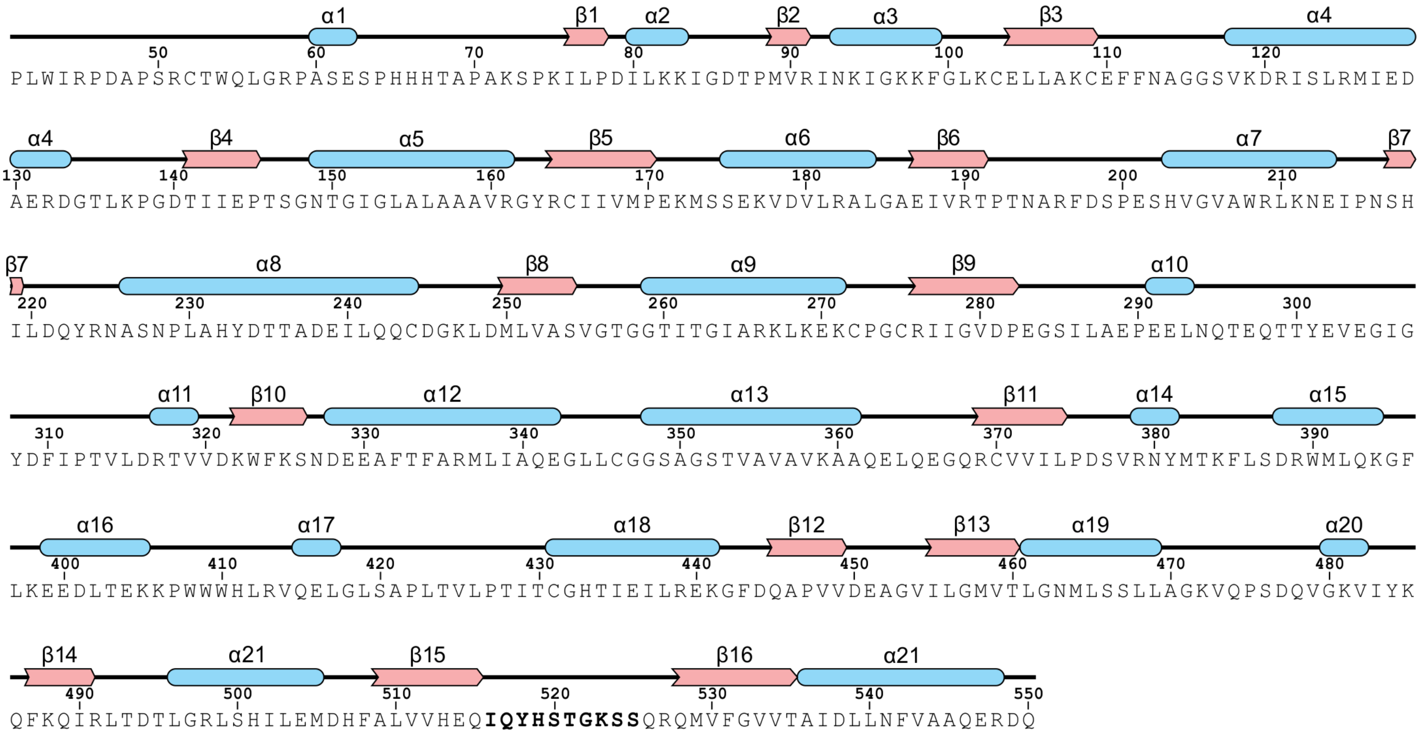

Supplementary figure 2. Cryo-EM image processing workflow for structural elucidation of human CBS in the absence of substrate and allosteric ligands. **A** – Representative electron micrograph showing long filamentous assemblies of WT-CBS. **B&C** – Representative 2D class averages (**B**) and 3D classification density maps (**C**) obtained using the helical reconstruction approach. **D&E** – Representative 2D class averages (**D**) and 3D classification maps (**E**) obtained using the single-particle analysis (SPA) approach. **F** – Final D1-symmetrized reconstruction of the *trans*-basal CBS filament from the SPA shown with local resolution map and the corresponding Fourier shell correlation (FSC) curve, as well as particle orientation distribution (**I**). **G** – Final helically reconstructed map of the *trans*-basal CBS filament at 3.0 Å resolution, shown with local resolution estimation and the corresponding FSC curve (**H**).

The *trans*-basal CBS filament (dataset 1)

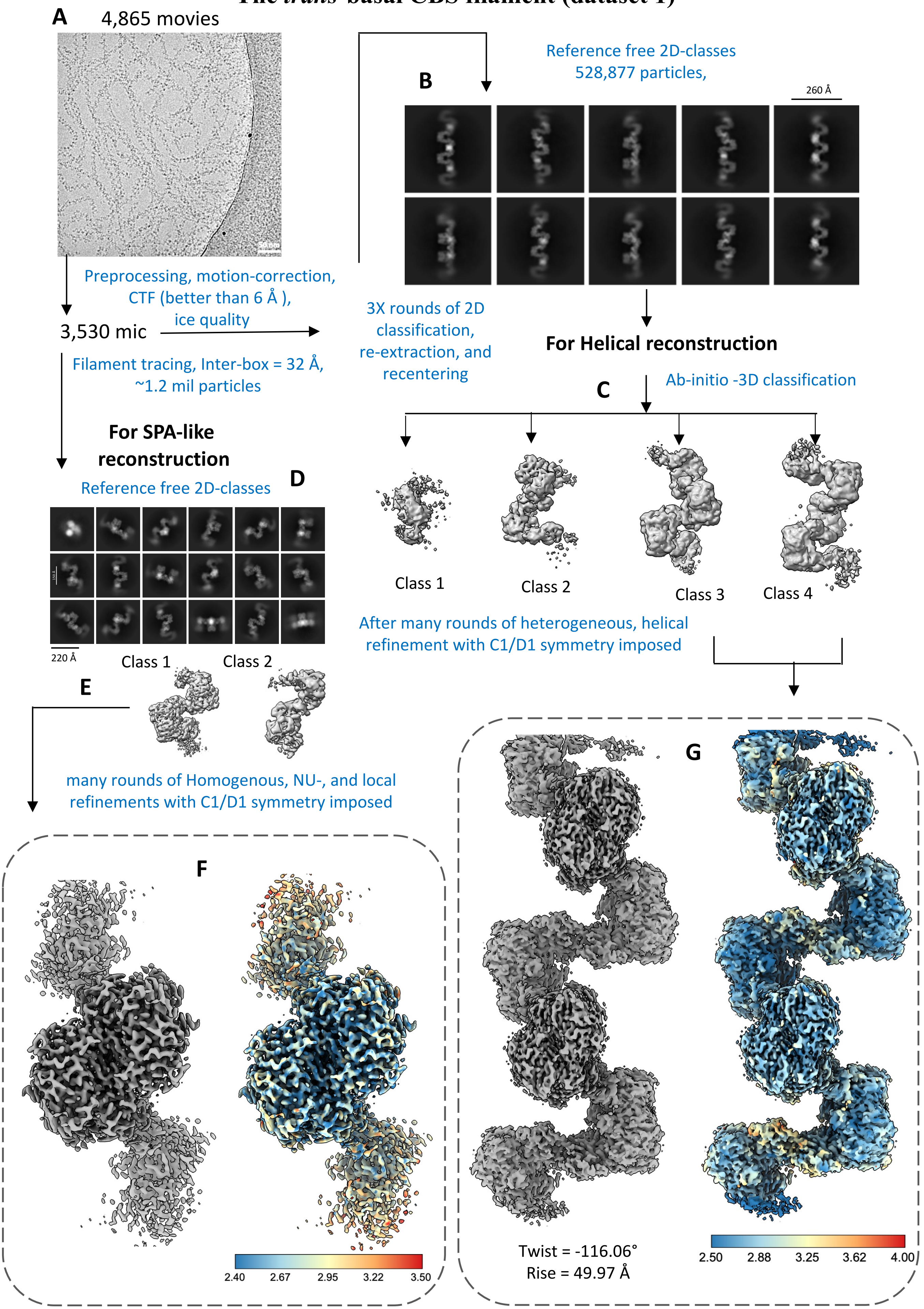

Supplementary figure 2

The *trans*-basal CBS filament (dataset 1)

Helical reconstruction

H

D1 symmetry imposed

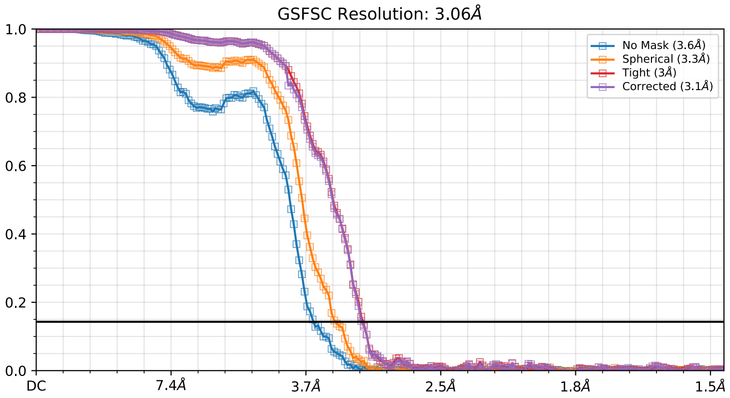

Twist = -116.06°
Rise = 49.97 Å

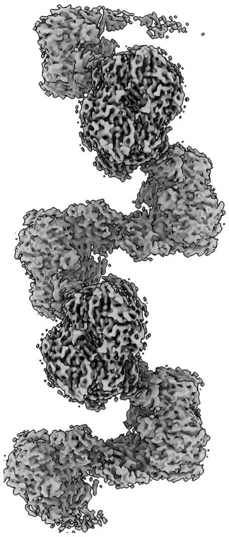

SPA-like reconstruction

I

D1 symmetry imposed

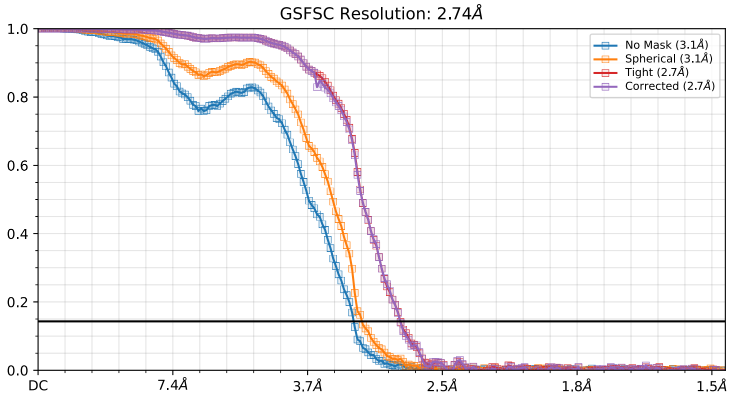

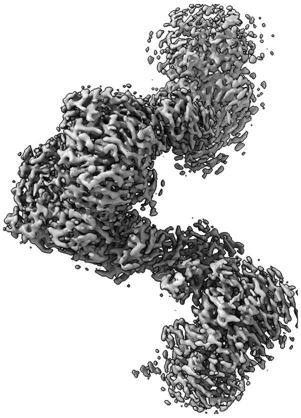

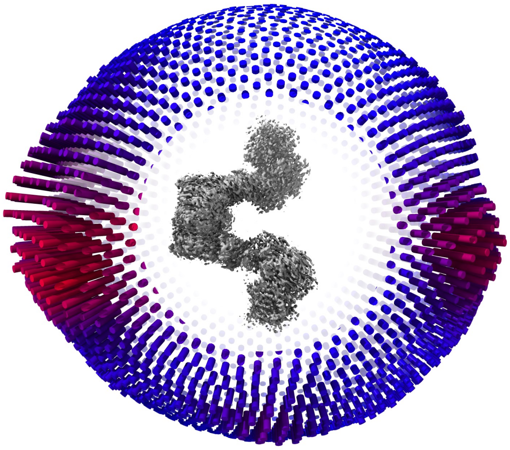

Supplementary figure 2 (continued)

Supplementary figure 3. Cryo-EM analysis for determination of helical parameters of the *trans*-basal CBS filament. A – Ab initio helical reconstruction of a CBS filament containing approximately 14–16 dimeric repeating units, used to estimate the helical pitch and rise in real space with UCSF Chimera. **B** – Representative 2D class average of the same long filament together with its corresponding power spectrum, used to calculate the helical parameters in the Fourier domain. Both real-space and Fourier analyses provided consistent initial estimates of ~164 Å pitch and ~54 Å rise. **C** – Validation of these parameters by refining and reconstructing a longer filament comprising 12–14 dimer repeats. The resulting map, fitted model, and FSC curve are shown, reaching a resolution of 4.71 Å.

The *trans*-basal CBS filament (dataset 1)

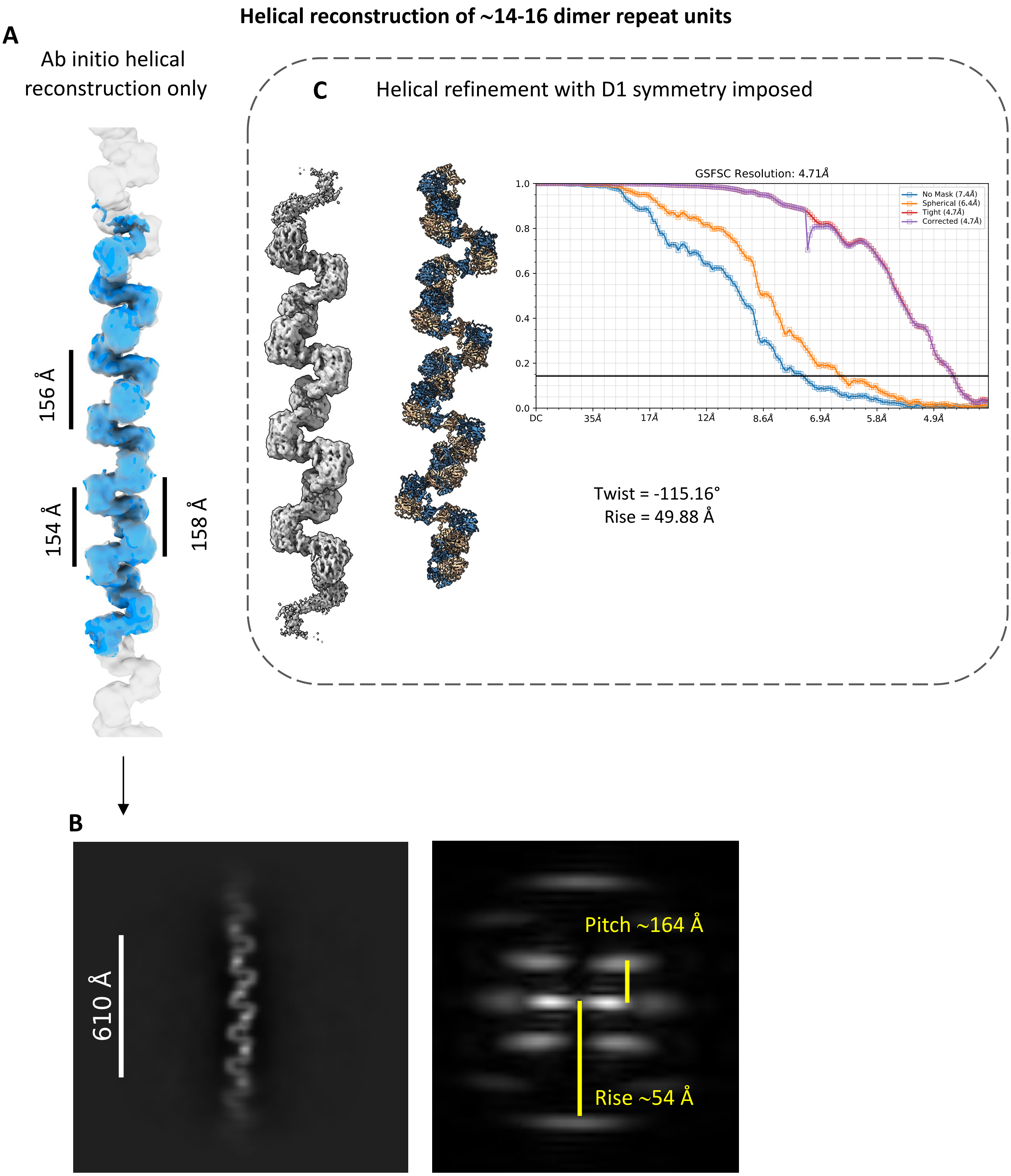

Supplementary figure 3

Supplementary figure 4. Role of CBS filamentation in protein stability – oligomerization loop variants. **A** – Native PAGE (top) and SDS-PAGE (bottom) Western blots showing that compared to the original CBS oligomerization loop construct CBSΔ516-525, both the 2-residues-shorter CBSΔ515-522 and longer CBSΔ515-526 oligomerization loop variants forms predominantly dimers (biological n=3). **B** – AzMC-based fluorescence assay shows that these loop variants retain normal CBS activity and response to 200 μM SAM similar to CBS WT and CBSΔ515-526 (n=18 independent repeats). Data are presented as mean values ± SEM and were analyzed with one-way ANOVA followed by Tukey's multiple comparison test (*p < 0.0001 compared to WT in the absence of SAM). **C** – Thermal activation profile (left) and the corresponding melting temperatures (right) of the studied variants showing significantly lower thermal stability of the loop variants compared to a full-length CBS WT (n=7 independent repeats). Data are presented as mean values ± SEM and were analyzed with one-way ANOVA followed by Tukey's multiple comparison test (*p < 0.0001 compared to WT). **D** – Thermal stability profile (top) and the corresponding melting temperatures (bottom) in the absence and presence of 200 μM SAM showing significantly lower thermal stability of the dimeric oligomerization loop variants compared to the filamentous full-length CBS WT (n=9 independent repeats). Data are presented as mean values ± SEM and were analyzed with one-way ANOVA followed by Tukey's multiple comparison test (*p < 0.0001 compared to WT in the absence of SAM).

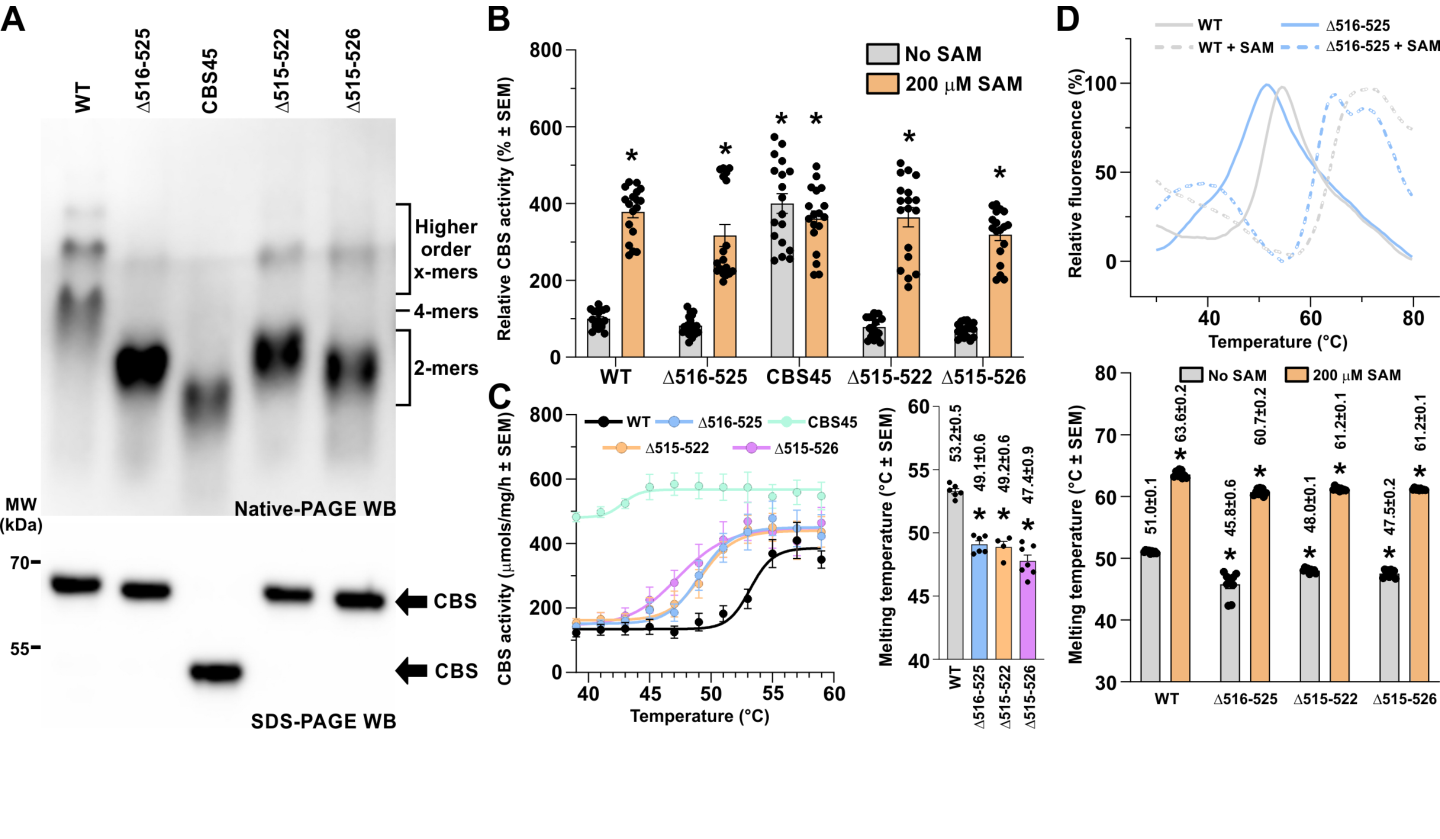

Supplementary figure 4

Supplementary figure 5. Role of CBS filamentation in protein stability – oligomerization loop point mutants. **A** – Native PAGE (top) and SDS-PAGE (bottom) Western blots showing that point mutations I516G, Q517G and/or Y518G yielded dimeric CBS constructs similar to CBSΔ516-525 (biological n=3). **B** – AzMC-based fluorescence assay shows that these point mutants retain normal CBS activity and response to 200 μM SAM similar to CBS WT and CBSΔ515-526 (n=4 independent repeats). Data are presented as mean values ± SEM and were analyzed with one-way ANOVA followed by Tukey's multiple comparison test (*p < 0.0001 compared to WT in the absence of SAM). **C** – Thermal activation profile (left) and the corresponding melting temperatures (right) of the studied point mutants showing CBS activation at significantly lower temperature compared to a full-length CBS WT (n=3 independent repeats). Data are presented as mean values ± SEM and were analyzed with one-way ANOVA followed by Tukey's multiple comparison test (*p < 0.0001 compared to WT). **D** – Determination of cellular turnover of the studied point mutants showing representative gels and blots used for signal quantification, normalization, plotting of the CBS decay (right) and calculation of cellular CBS half-life. Similar to CBSΔ516-525, the dimeric oligomerization loop point mutants showed significantly faster cellular turnover compared to filamentous CBS WT (biological n=3). Data are presented as mean values ± SEM and the resulting half-lives were analyzed with one-way ANOVA followed by Dunnett's multiple comparison test (*p values equal to 0.0312, 0.0426, 0.0269 and 0.0019 comparing WT with the I516G, Q517G, Y518G and I516G+Q517G+Y517G CBS mutants, respectively).

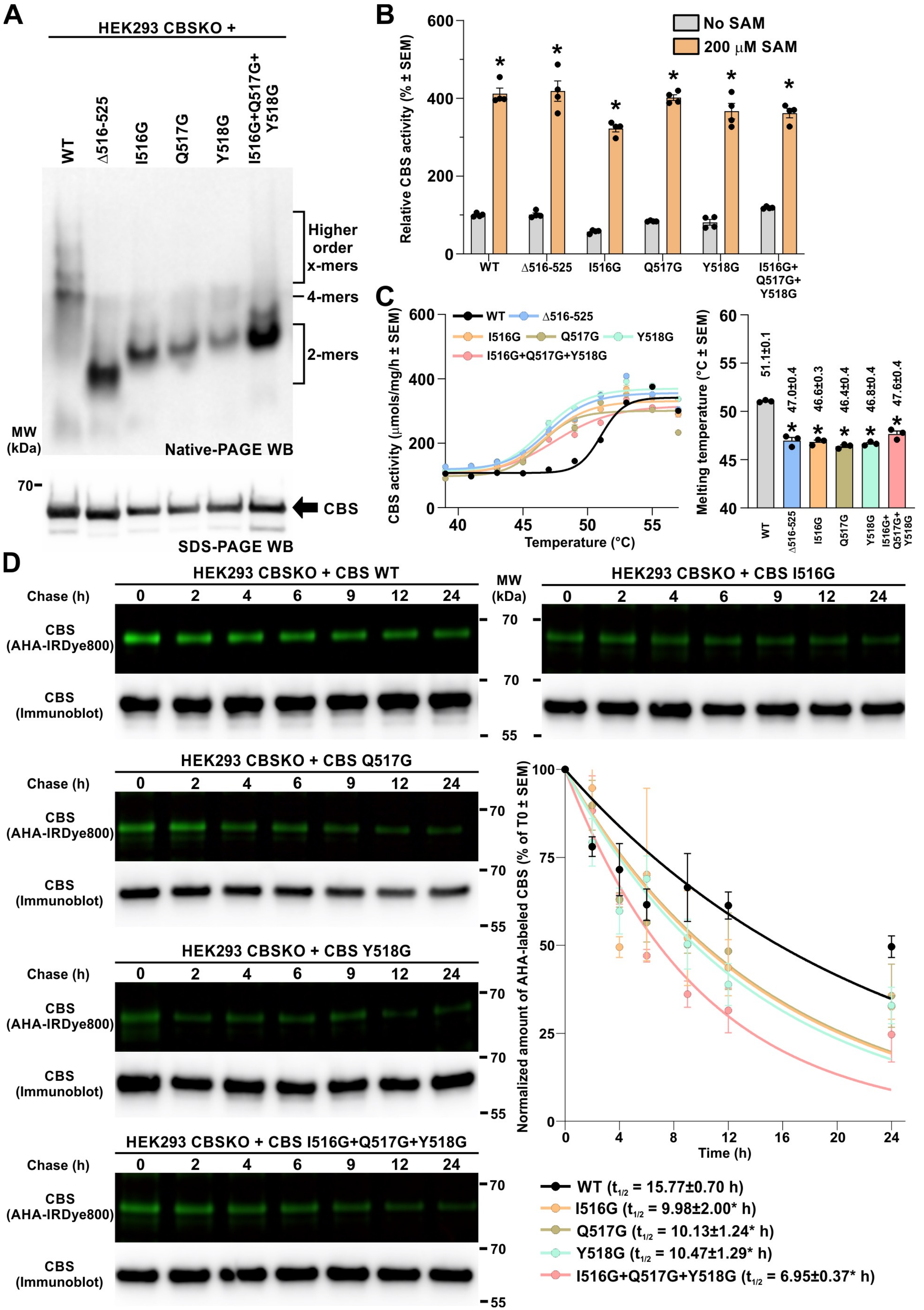

Supplementary figure 5

Supplementary figure 6. Cryo-EM image processing workflow for structural elucidation of human CBS in the presence of its substrate L-serine. **A** – Processing pipeline combining both helical and single-particle analysis (SPA)-like approaches. Representative reference-free 2D class averages are shown. Particles contributing to a consensus map were subsequently used to sort and classify substates by both strategies: SPA classification with a focused mask around one CD dimer repeat and four RDs, and helical classification with a mask covering three dimer repeats. **B&C** – This approach resolved three well-defined structural classes, corresponding to the key catalytic intermediates: CBS-Lys-PLP (class 3), CBS-PLP-Ser (class 4), and CBS-PLP-AA (class 5).

The Ser-*trans*-basal CBS filament (dataset 2)

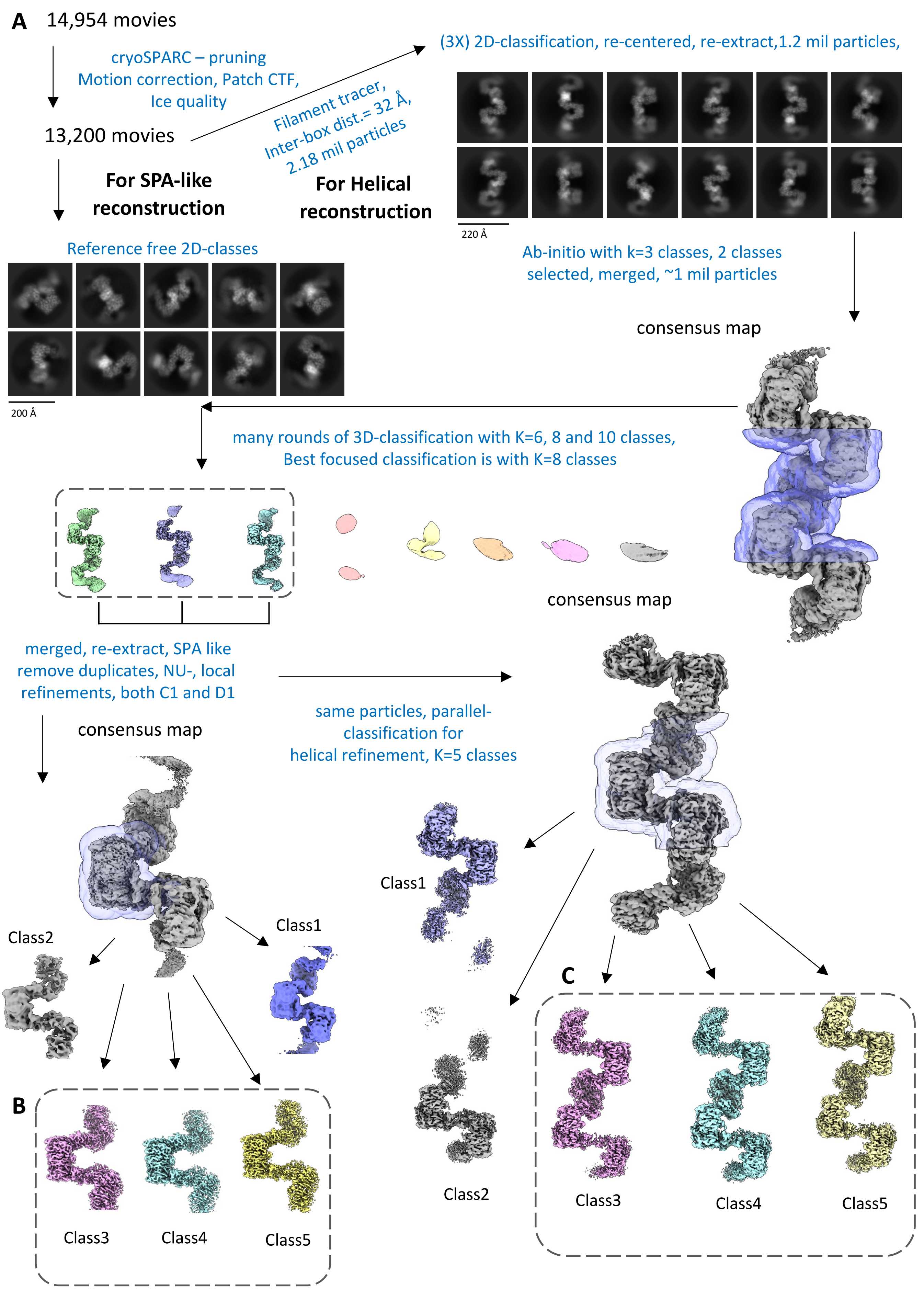

Supplementary figure 6

Supplementary figure 7. Cryo-EM helical reconstructions of L-serine substrate-bound CBS *trans*-basal filaments. **A** – CBS-PLP-AA (class 5), representing the PLP–aminoacrylate intermediate, resolved at 2.62 Å. The panel includes the local resolution map and corresponding FSC curve. **B** – CBS-PLP-Ser (class 4), representing the PLP–serine external aldimine intermediate, resolved at 2.92 Å. The global resolution is shown by the FSC curve alongside the local resolution map.

The Ser-*trans*-basal CBS filament (dataset 2)

Helical reconstruction

A

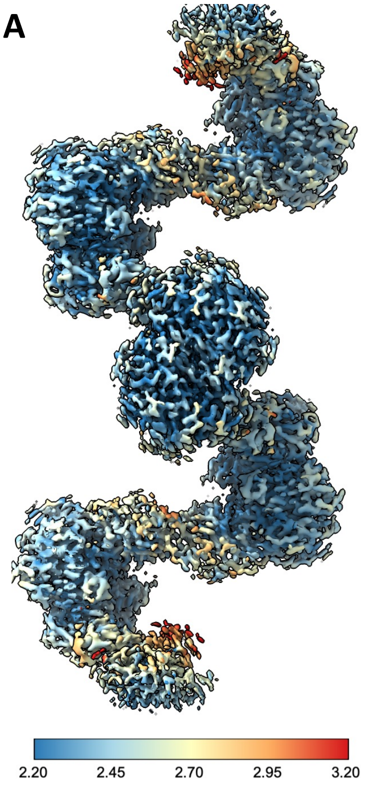

Class5, D1 symmetry imposed,
Twist = -115.312°, Rise = 49.940 Å

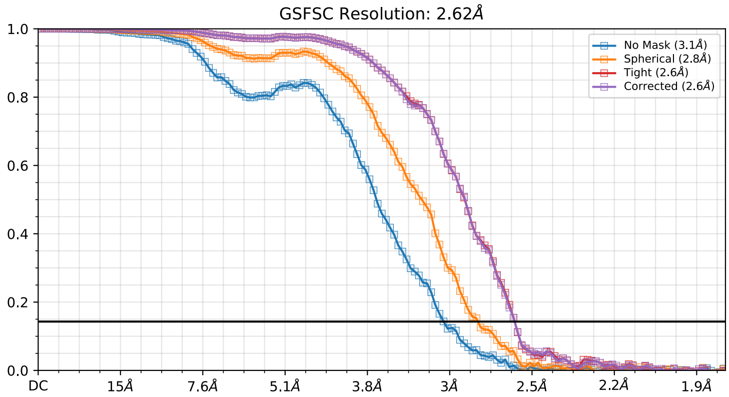

B

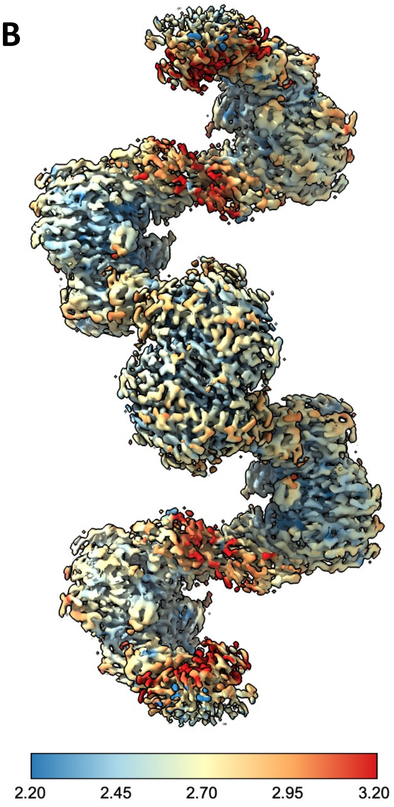

Class4, D1 symmetry imposed,
Twist = -114.984°, Rise = 50.78 Å

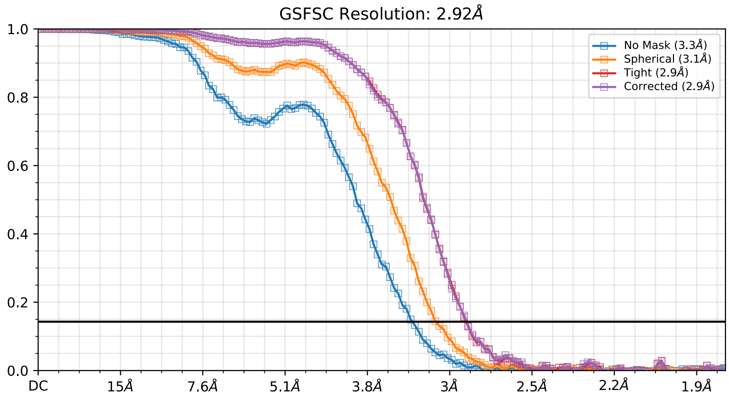

Supplementary figure 7

Supplementary figure 8. Cryo-EM single-particle reconstructions of L-serine substrate-bound CBS *trans*-basal filaments. **A** – CBS-PLP-AA (class 5), corresponding to the PLP–aminoacrylate intermediate, reconstructed at 2.08 Å resolution. The panel shows the local resolution map, FSC curve, and particle orientation distribution. **B** – CBS-PLP-Ser (class 4), corresponding to the PLP–serine external aldimine intermediate, reconstructed at 2.20 Å resolution. The global resolution is validated by the FSC curve, with accompanying local resolution and particle orientation distribution maps.

The Ser-*trans*-basal CBS filament (dataset 2)

SPA-like reconstruction

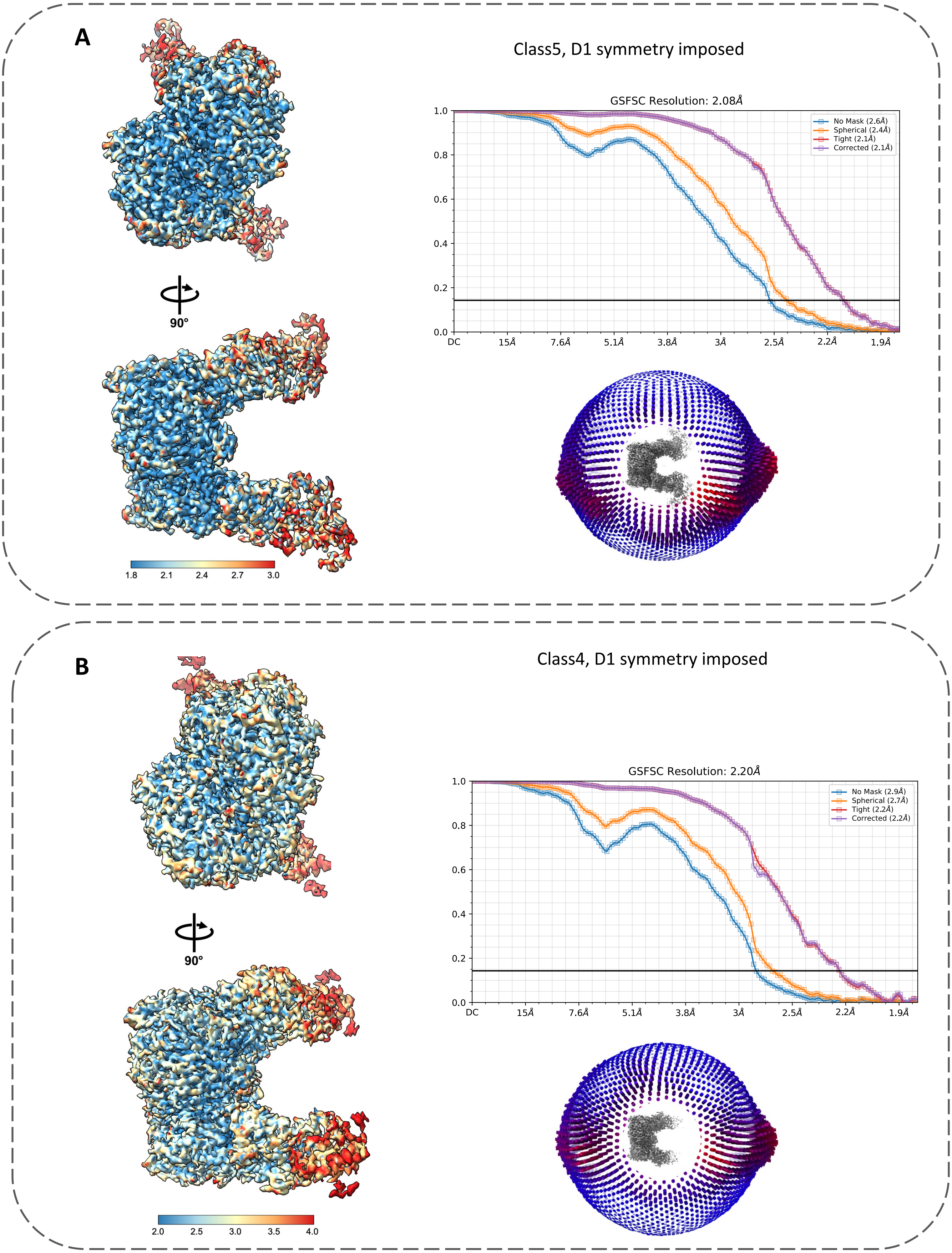

Supplementary figure 8

Supplementary figure 9. Active-site remodeling during serine turnover – matched snapshots of PLP intermediates. Three LigPlot+ interaction diagrams are shown for the serine-bound dataset, displayed in the same orientation and with consistent residue labeling to emphasize progressive closure of the catalytic pocket during catalysis: (**A**) internal aldimine (CBS-Lys-PLP), (**B**) external aldimine (CBS PLP-Ser), and (**C**) PLP–aminoacrylate (CBS PLP-AA). Compaction is illustrated by (i) coordinated repositioning and engagement of serine-contacting residues S147, T150, T146, Q222, and N149 upon external aldimine formation, (ii) further tightening around the PLP-reactive center as the aminoacrylate intermediate forms, and (iii) preservation of the phosphate anchoring network T257/T260 and the G256–G259 loop, which remains largely unchanged and serves as a structural pivot during these transitions. In the diagrams, green dashed lines indicate hydrogen-bonding interactions, red "sunburst" arcs denote hydrophobic/van der Waals contacts between ligand atoms and nearby residues, and residues drawn as light-brown sticks represent polar/charged side chains positioned to form stabilizing electrostatic and/or hydrogen-bond interactions with the PLP adducts and associated intermediates, depicted here with bold green.

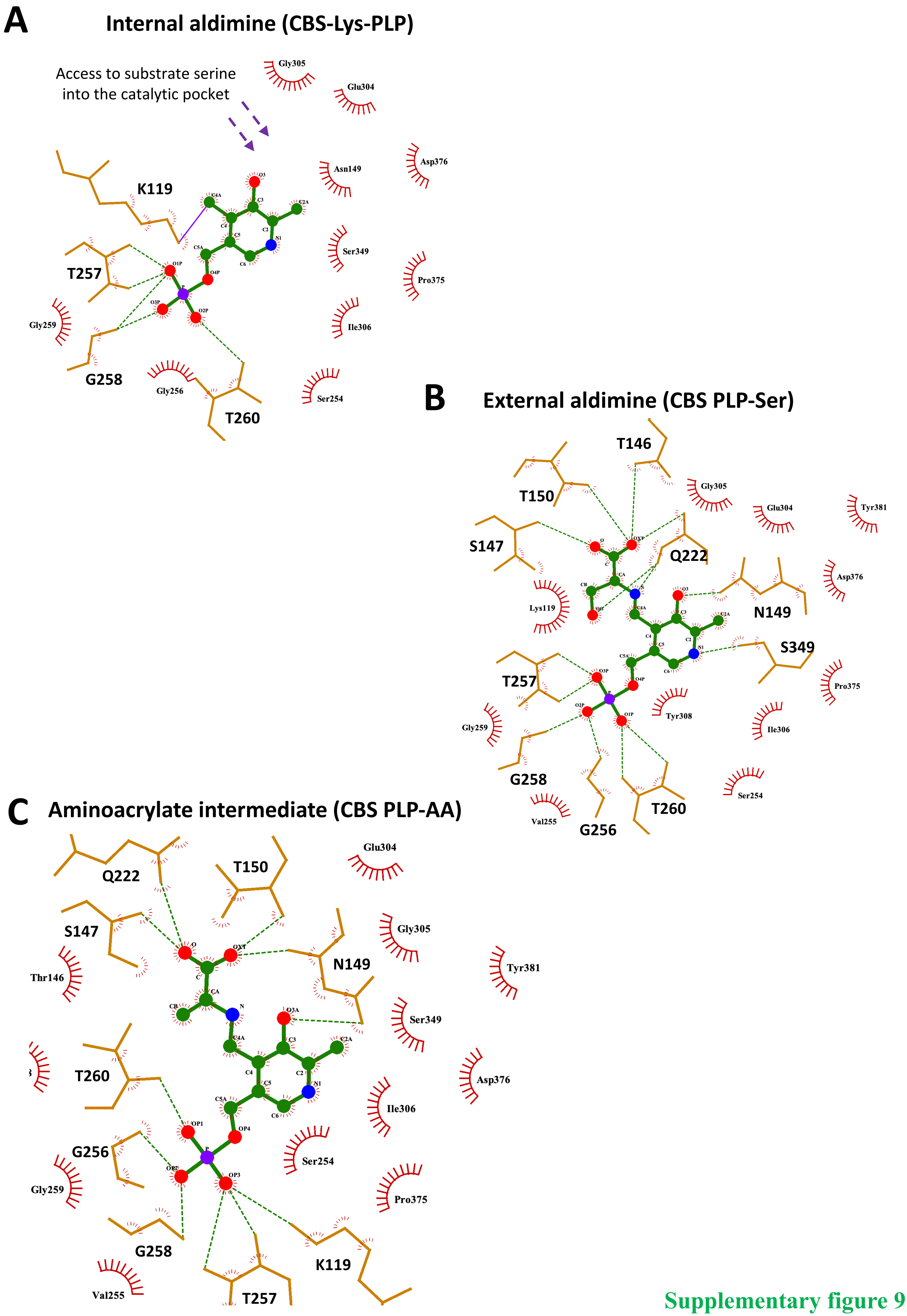

Supplementary figure 9

Supplementary figure 10. Cryo-EM image processing workflow of the *cis*-basal CBS filament in the presence of a non-activating allosteric ligand sinefungin/adenosylornithine (SAO). A – Representative electron micrograph of human CBS incubated with 0.5 mM SAO, showing long filamentous assemblies with a distinct morphology compared to the *trans*-basal state. This is further illustrated in the corresponding 2D class averages. The processing workflow combined both helical reconstruction and SPA-like approaches, beginning with reference-free 2D class averaging. Particles contributing to a consensus reconstruction were subsequently sorted and classified to exclude suboptimal images. **B&C** – Helical classification and refinement strategies. Panel **B** shows refinement using masks covering the entire repeats of both CDs and RDs, while panel **C** focuses on refinement with a localized mask around the central RDs only.

The SAO-bound *cis*-basal CBS filament (dataset 3)

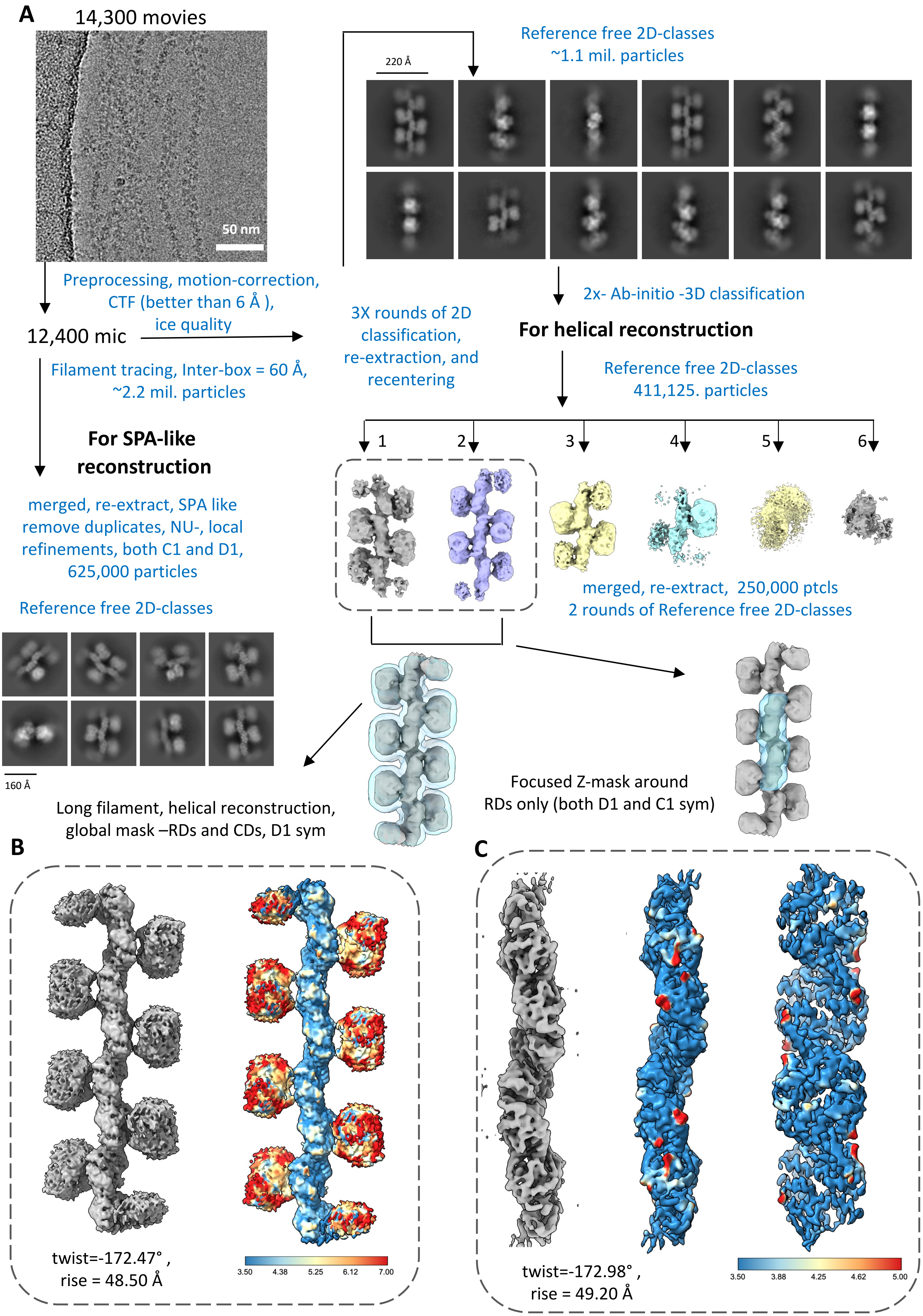

Supplementary figure 10

Supplementary figure 11. Cryo-EM helical reconstructions of SAO-bound *cis*-basal CBS filament. A – Helical refinement using masks covering the entire repeats of both CDs and RDs, yielding a reconstruction at 4.86 Å resolution as indicated by the FSC curve. **B** – Focused refinement with a localized Z-mask applied around the central RDs, which improved the map quality and allowed clearer visualization of the bound SAO ligand. The corresponding FSC curve is shown.

The SAO-bound *cis*-basal CBS filament (dataset 3)

Helical reconstruction

A

Long filament, helical reconstruction, global mask –RDs and CDs, D1 sym

Twist = -172.47°
Rise = 48.50 Å

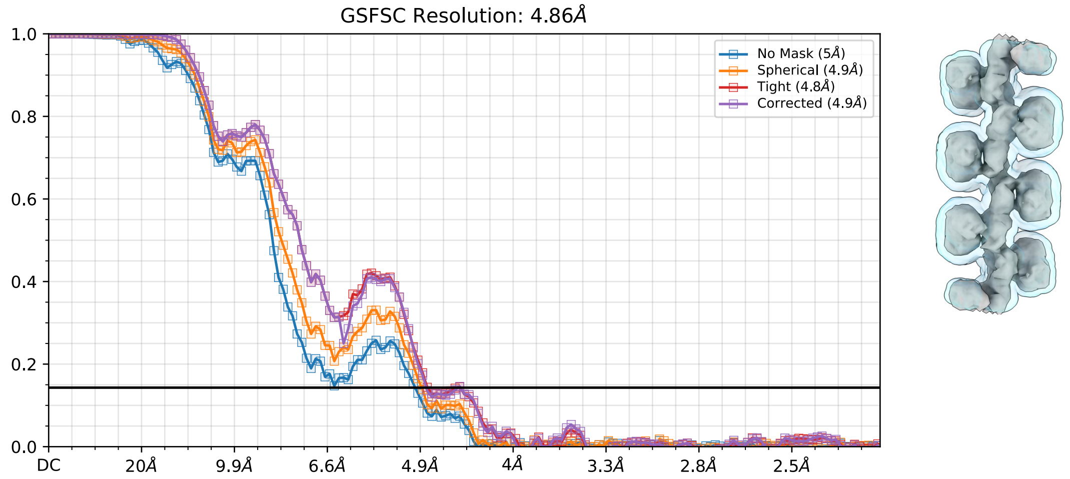

B

Focused Z-mask around RDs only (both D1- and C2-sym)

Twist = -172.98°
Rise = 49.20 Å

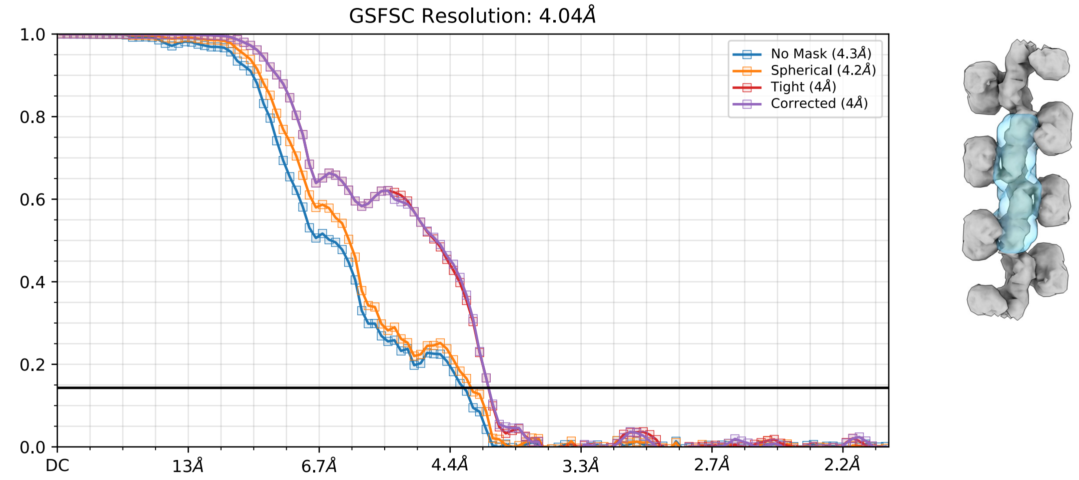

Supplementary figure 11

Supplementary figure 12. Cryo-EM single-particle reconstruction of cis-basal CBS in the presence of the SAO ligand. A&B – Representative 2D class averages (**A**) and the resulting 3D reconstruction (**B**) obtained using a focused mask encompassing one CD and six RD domains. The local resolution map, refined to 4.1 Å, shows that the CD is resolved at slightly lower resolution compared to the central RD region. **C** – FSC curve and particle orientation distribution corresponding to the reconstruction.

The SAO-bound *cis*-basal CBS filament (dataset 3)

SPA-like reconstruction

A

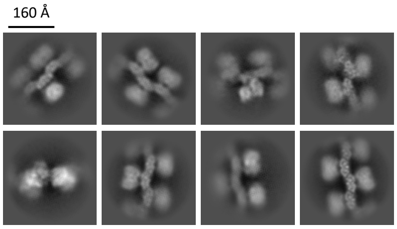

Reference free 2D-classes
106,300 particles
D1 symmetry imposed

B

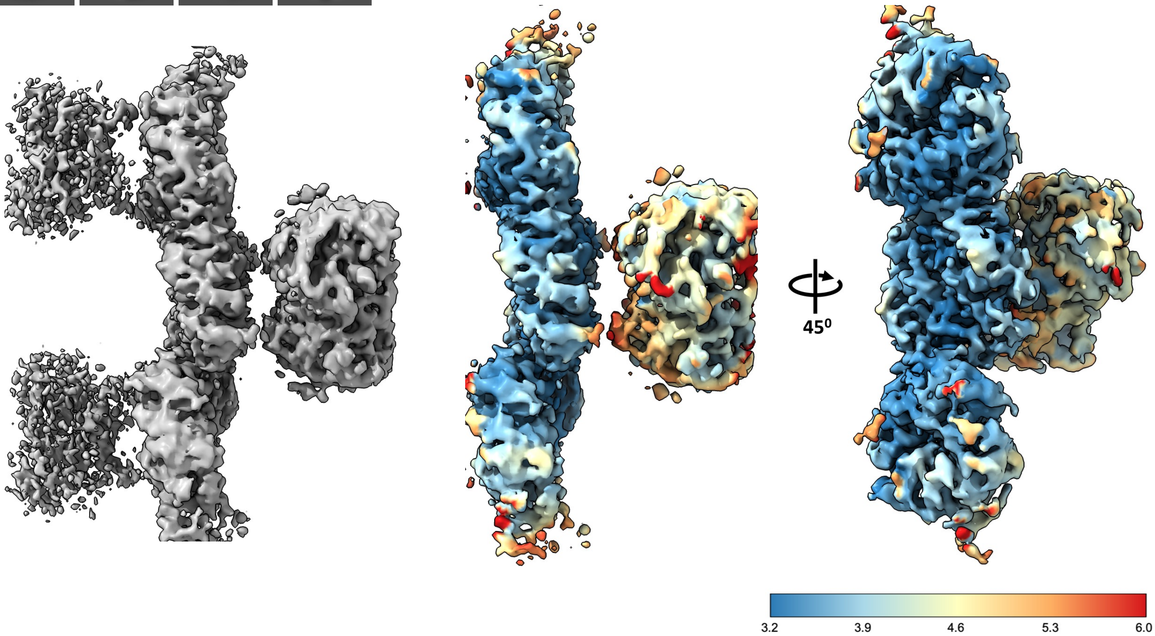

C

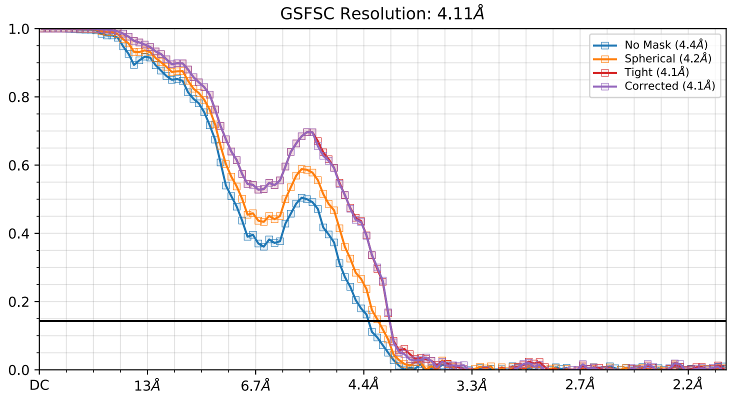

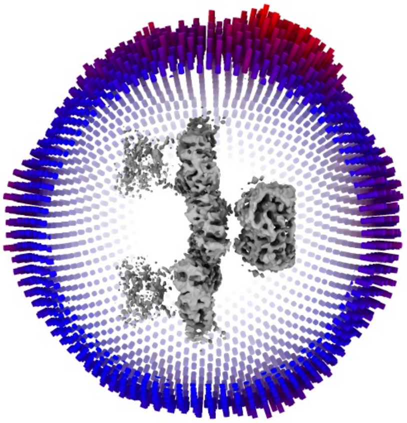

Supplementary figure 12

Supplementary figure 13. Additional molecular assemblies and disassembly states observed in the SAO and SAM datasets. **A** – Broken dimeric assemblies containing two CDs and two RDs identified in both the SAO- and SAM-bound datasets. Below, extended dimers consisting of two CDs and four RDs were also observed. **B** – A unique assembly observed only in the SAO dataset, possibly representing a transitional intermediate captured during the shift from the ligand-free *trans*-basal to the SAO-bound *cis*-basal state. **C** – Long filamentous assemblies of RDs detected in 2D class averages. **D** – Isolated densities corresponding to broken central *allo*-CDs observed only in the SAM-bound dataset, likely representing disassembled intermediates of the resolved *allo*-activated stacked CBS filament.

Other molecular CBS species observed in of SAO- and SAM-bound datasets

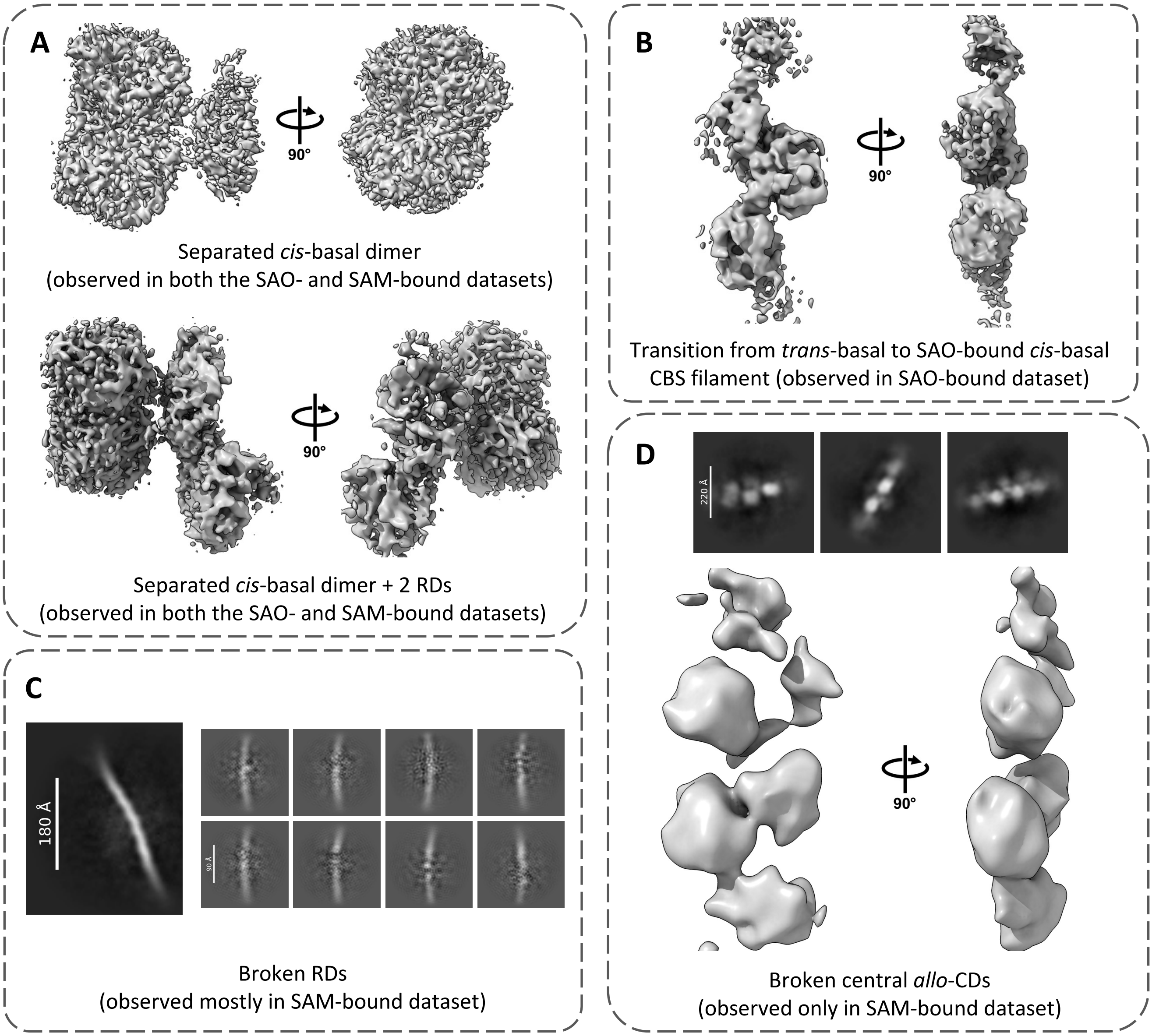

Supplementary figure 13

Supplementary figure 14. Cryo-EM reconstructions of cis-basal CBS in the presence of SAM. A – Helical reconstruction approach revealing a minor population of SAM-bound *cis*-basal CBS filaments. Representative 2D class averages, the corresponding 3D reconstruction, local resolution map, and FSC curve are shown, yielding a final resolution of 6.76 Å. **B** – SPA-like reconstruction of the SAM-bound *cis*-basal CBS filament using a focused mask around the central RD region. The corresponding FSC plots and local resolution maps are shown, demonstrating that the RDs are better resolved than the CDs. This refinement yielded a map at 4.12 Å resolution.

The SAM-bound *cis*-basal CBS filament (minor fraction; dataset 4)

Helical reconstruction

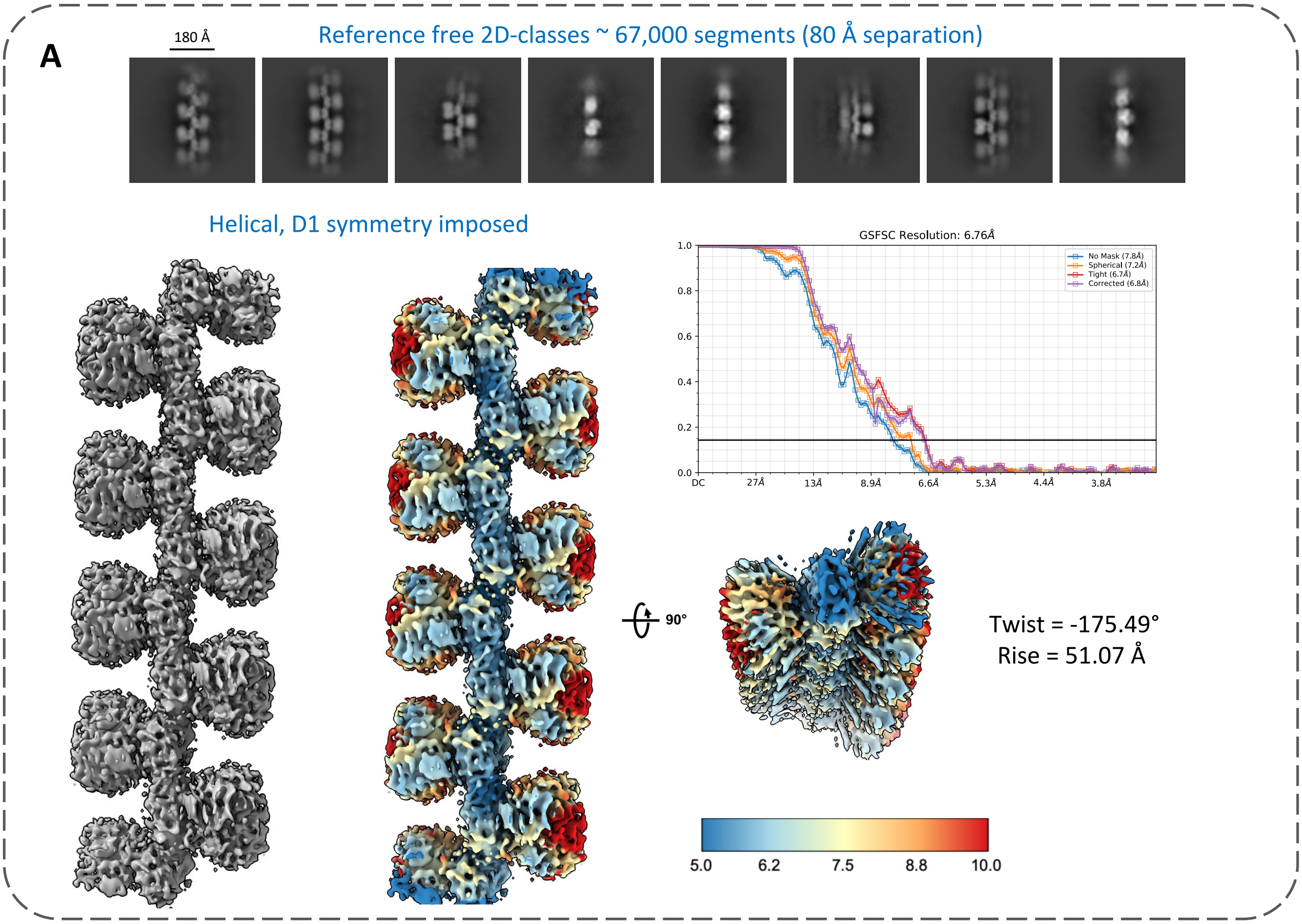

SPA-like reconstruction

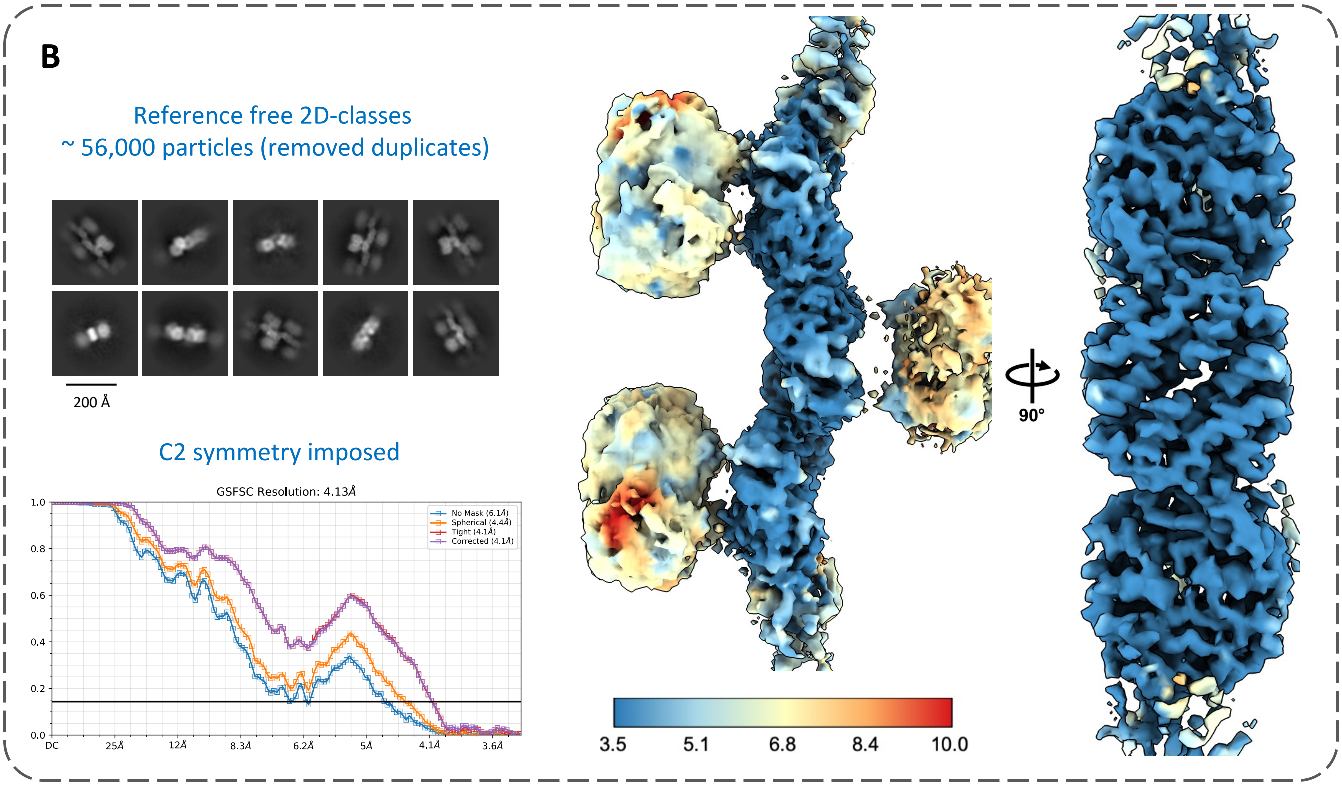

Supplementary figure 14

Supplementary figure 15. Cryo-EM image processing workflow for structural elucidation of human CBS in the presence of an activating allosteric ligand S-adenosylmethionine (SAM). A – Representative electron micrograph showing higher-order filamentous assemblies of human full-length CBS in the presence of 0.5 mM SAM (highlighted with yellow markers). These assemblies display distinct morphology compared to both the *trans*-basal and SAO-bound *cis*-basal CBS filaments. **B&C** – Representative 2D class averages (**B**) and 3D classification density maps (**C**) obtained from ab initio and helical reconstructions performed without imposing helical parameters. **D** – After multiple rounds of 3D classification, the best classes were merged, re-extracted with a larger box size, and subjected to additional 2D classification to exclude suboptimal particles. The resulting averages reveal stacked filaments with a core formed uniquely rearranged CDs termed "*allo*-CDs" (yellow arrows).

The SAM-bound *allo*-activated stacked CBS filament (major fraction; dataset 4)

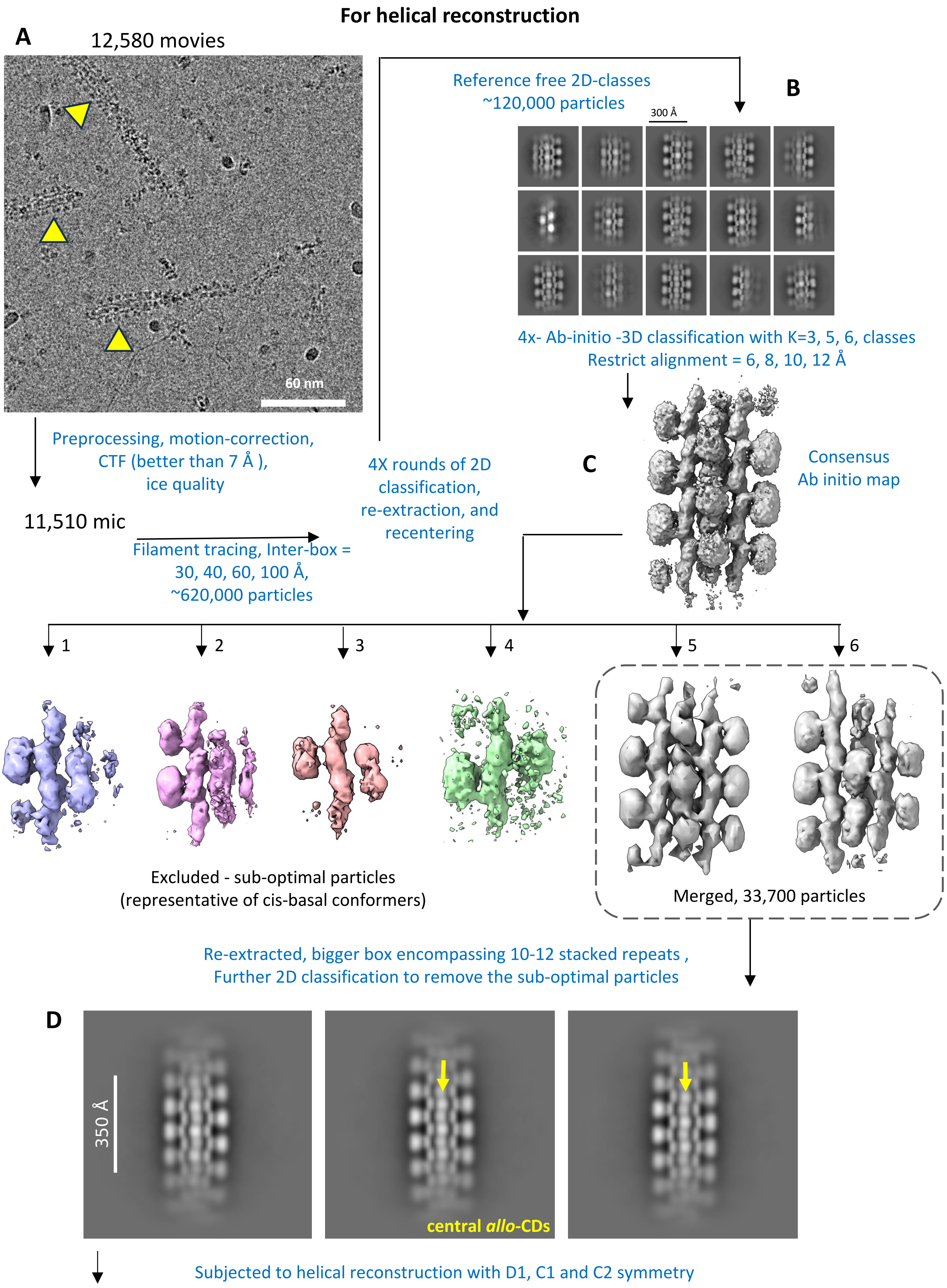

Supplementary figure 15

Supplementary figure 16. Cryo-EM helical reconstructions of the SAM-bound *allo*-activated stacked CBS filament. A – Helical reconstruction and refinement performed using masks covering two consecutive repeats of both CDs and RDs. Reconstruction was carried out without imposing symmetry (C1) to allow visualization of the central *allo*-CD region, which is stacked between the two incoming filaments. The CD-RD connecting linker motifs, RDs, outer CDs, and central *allo*-CDs are designated. **B** – The final reconstruction was resolved at 8.43 Å, as indicated by the FSC curve. The corresponding local resolution map shows that the flanking RDs are better defined compared to the central *allo*-CDs.

The SAM-bound *allo*-activated stacked CBS filament (major fraction; dataset 4)

Helical reconstruction

Final helical reconstruction = after many rounds of D1 symmetry and followed by C1 symmetry

A

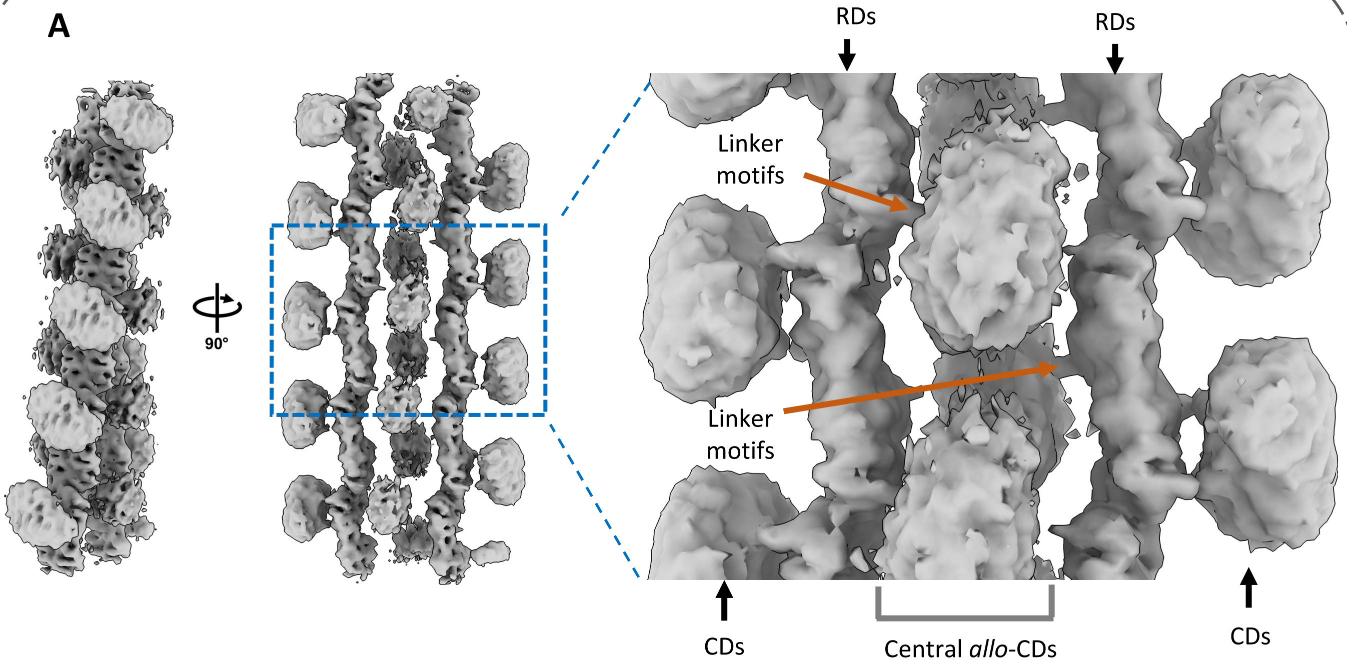

C1 symmetry imposed

B

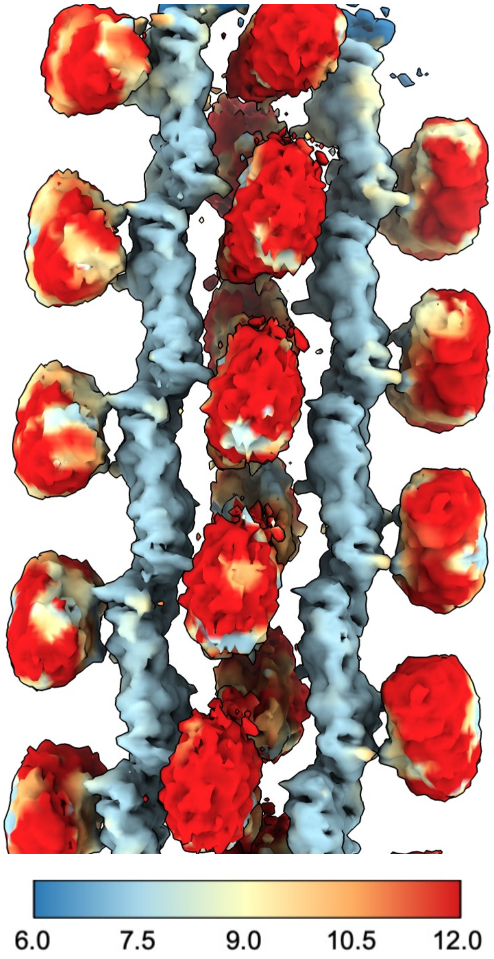

Twist = -176.08°
Rise = 48.28 Å

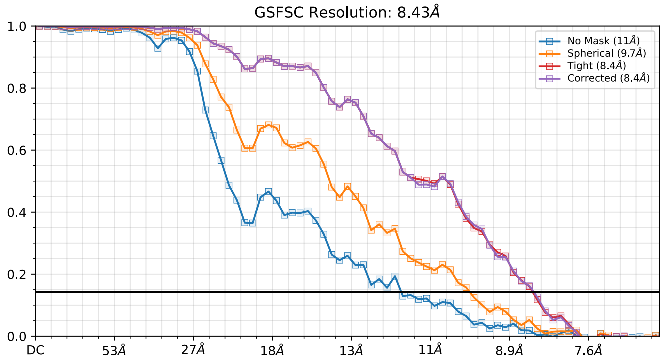

Supplementary figure 16

Supplementary figure 17. Complementary cryo-EM helical reconstruction of the SAM-bound *allo*-activated stacked CBS filament with C2 symmetry imposed. **A** – Helical reconstruction and refinement carried out with C2 symmetry applied, aimed at improving the resolution of the central *allo*-CD region stacked between the two incoming filaments. The RDs, CDs, and central *allo*-CDs are highlighted. **B** – The reconstruction reached 9.65 Å resolution, as indicated by the FSC curve. The corresponding local resolution map shows no improvement in density for the central *allo*-CDs compared to the C1 reconstruction (Supplementary figure 10), underscoring its intrinsic flexibility.

The SAM-bound *allo*-activated stacked CBS filament (major fraction; dataset 4)

Helical reconstruction

A Helical reconstruction = C2 symmetry imposed to resolve the central CDs

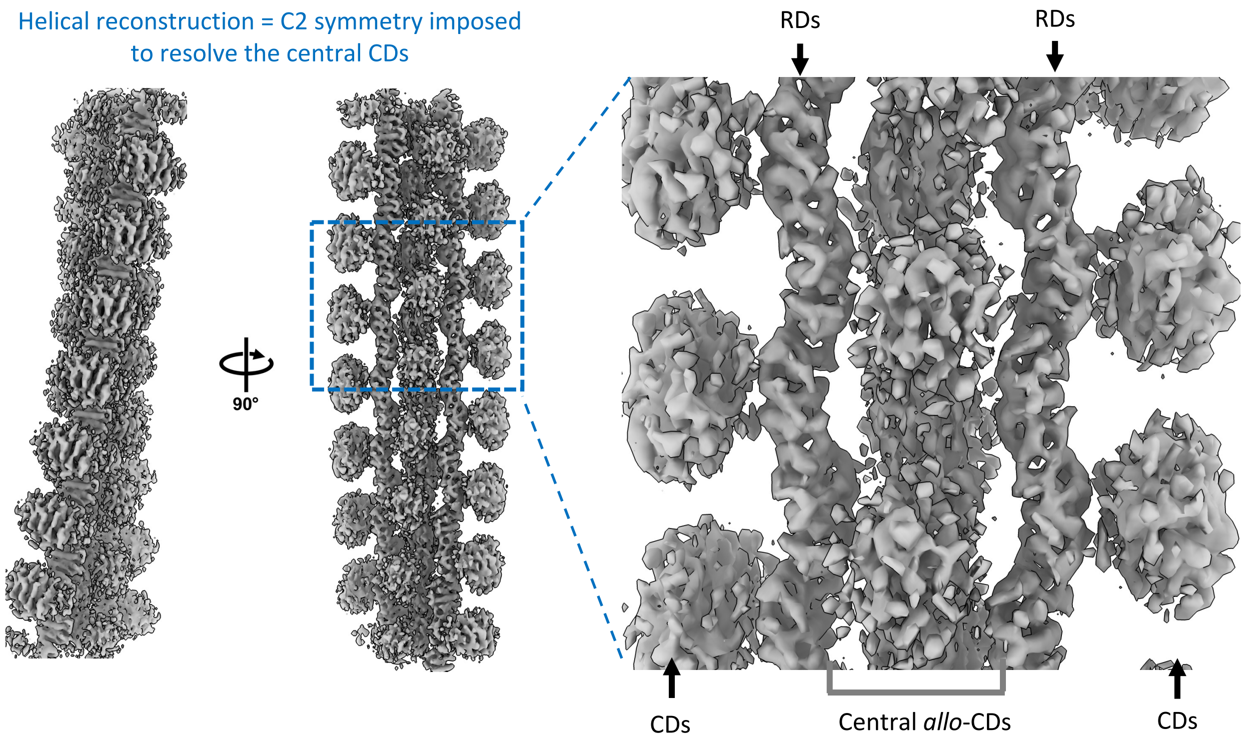

B C2 symmetry imposed

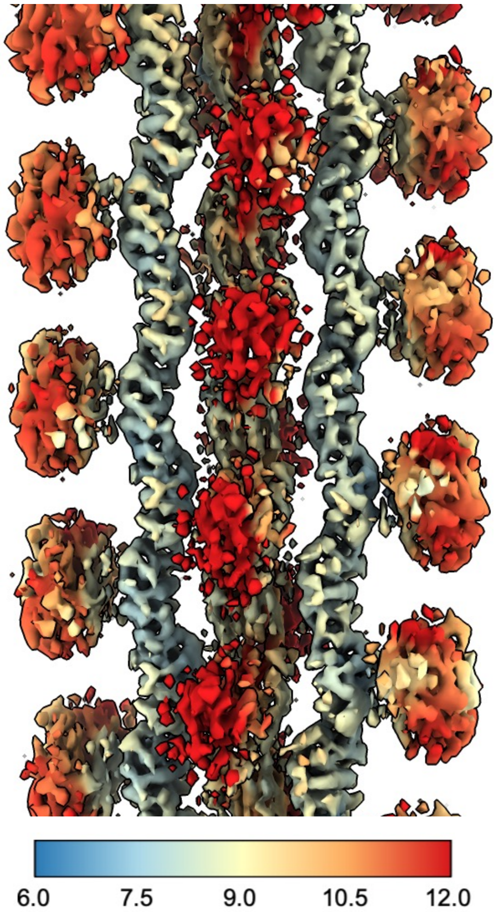

Twist = -174.766°
Rise = 48.150 Å

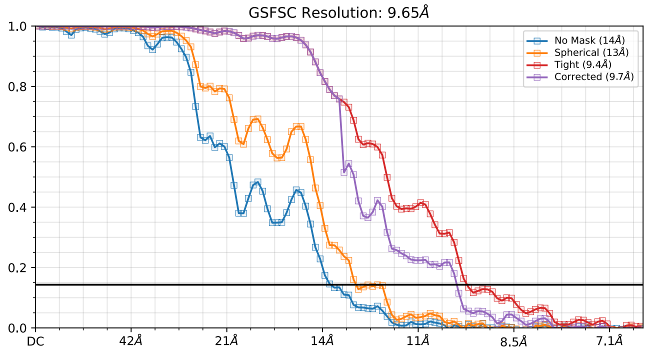

Supplementary figure 17

Supplementary figure 18. Cryo-EM single-particle reconstruction of the SAM-bound *allo*-activated stacked CBS filament. A – Representative 2D class averages showing end-on and tilted views of the SAM-bound *allo*-activated stacked CBS filament. Yellow arrows highlight the central *allo*-CDs. **B** – Focused refinement using a localized Z-mask around the central *allo*-CD region with C2 symmetry imposed. **C** – Subsequent refinement with a full mask under C1 symmetry improved overall map quality, enabling clearer visualization of the central *allo*-CD stalk. The corresponding FSC curve and local resolution map are shown. This strategy yielded a global reconstruction at 7.87 Å resolution.

The SAM-bound *allo*-activated stacked CBS filament (major fraction; dataset 4)

SPA-like reconstruction

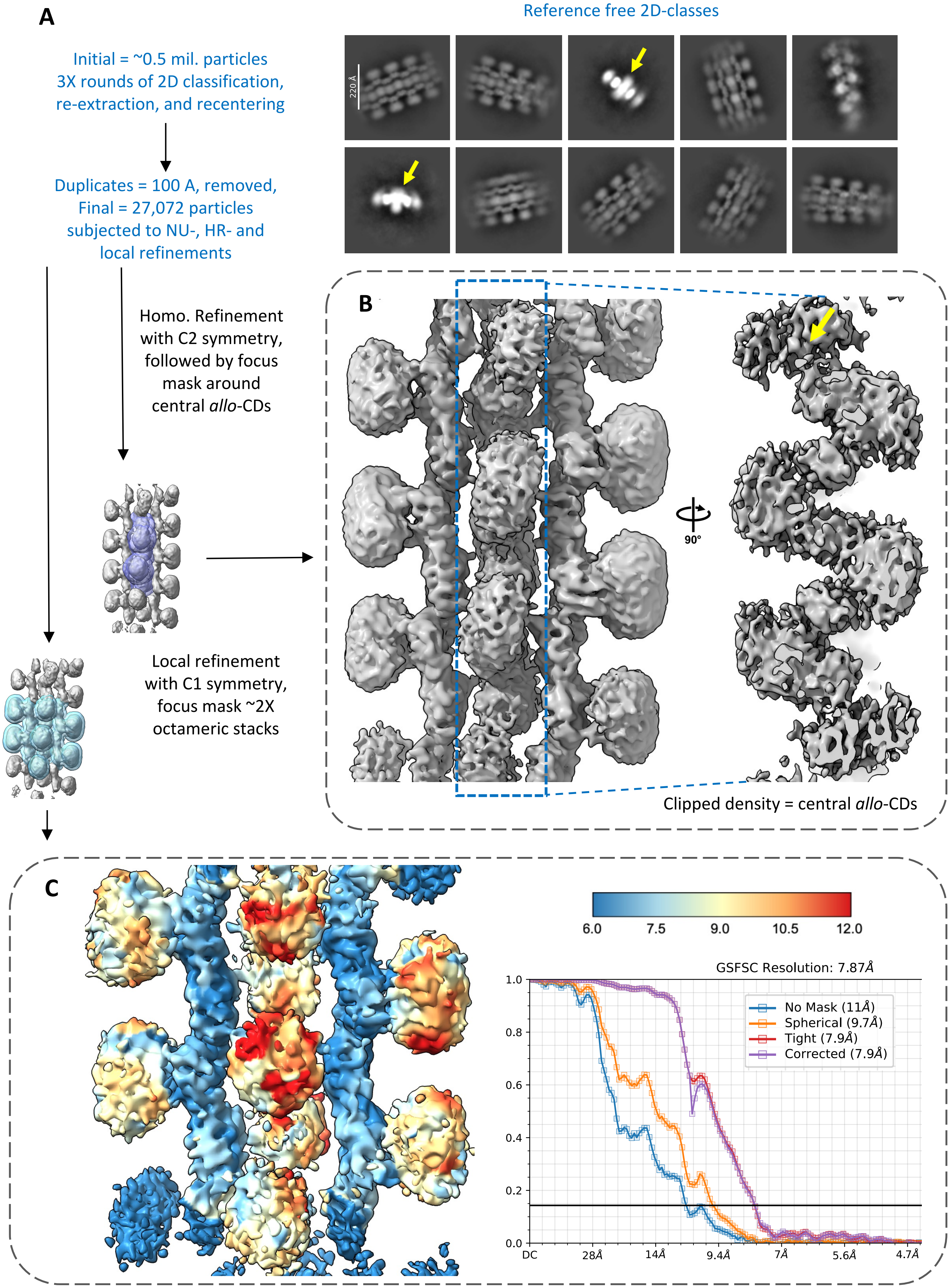

Supplementary figure 18

Supplementary figure 19. Structural superpositions of CBS assemblies reveal ligand-dependent rigid-body rearrangements that underpin the proposed morpheein model. **A** – Superposition of single protomers (grey: *trans*-basal, cyan: SAO-bound *cis*-basal, purple: SAM-bound *cis*-basal) aligned on the catalytic domain (CD) shows that the CD cores are highly conserved across states (minimal RMSD, < 1Å), whereas the regulatory domains (RDs) in the *cis*-basal conformers undergo a pronounced rigid-body displacement relative to the *trans*-basal state, characterized by an >90 Å translation and ~70° rotation, consistent with the formation of the CBS modules (**B**). **B** – Overlay of the SAO-bound *cis*-basal (cyan) and SAM-bound *cis*-basal (purple) dimers demonstrates near-identical architecture, with only minor deviations (~1–2 Å) localized near the oligomerization-loop region (residues 512–526). **C** – Superposition aligned on the RD loop (residues 512–534) comparing a SAM-bound *cis*-basal protomer (purple) with the central *allo*-state protomer (green) highlights a large relative repositioning of the CD, involving an >100 Å translation and ~140° rotation. This contrast is emphasized by comparison to the corresponding alignment between a central *allo*-state protomer (green) and the *trans*-basal protomer (grey) (bottom). **D** – RD-loop–anchored overlay of central *allo*-state dimers (green/red) with the *trans*-basal dimer (grey) shows that the *allo*-dimer CD–CD interface differs more subtly from the *trans*-basal arrangement, including a small separation across the CD dimer interface (~40 Å) and an angular offset (~25°), compared with a much larger *cis*-to-*allo* rearrangements. Black circles indicate the CD–RD connecting linker (helix–loop–helix containing consecutive tryptophane residues), which is positioned as a potential hinge coordinating large rigid-body movements between CD and RD during assembly transitions.

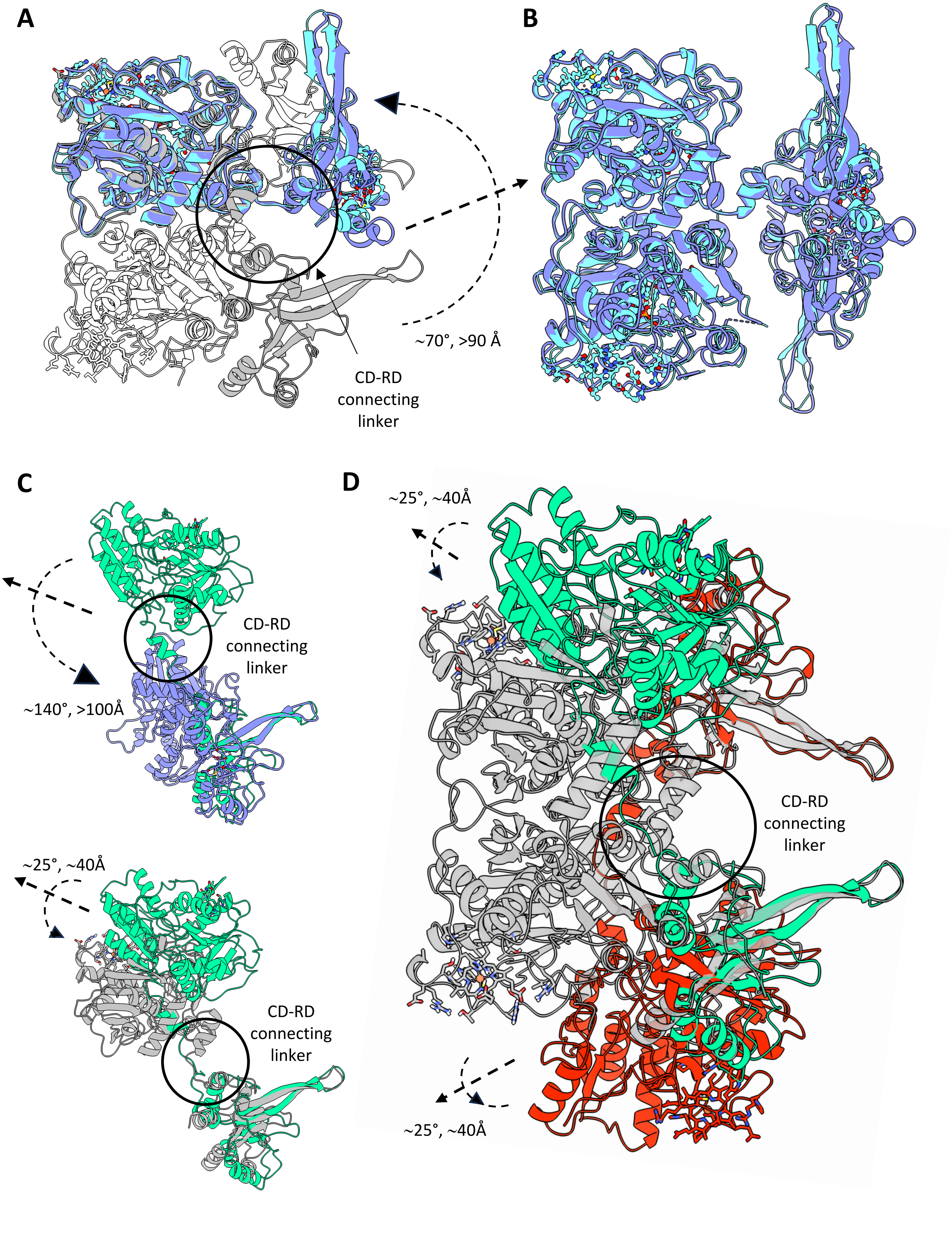

Supplementary figure 19

Supplementary figure 20. Role of CBS filamentation in protein stability – pathogenic HCU-causing mutants. **A** – Native PAGE (top) and SDS-PAGE (bottom) Western blots showing that pathogenic mutations P422L and Q526K do not impair ability of CBS to form higher order oligomers. Red dashed lines indicate splicing and removal of lanes containing other non-relevant CBS constructs (biological n=3). **B** – AzMC-based fluorescence assay shows that these pathogenic mutants retain normal CBS activity and response to 200 μM SAM similar to CBS WT (n=18 independent repeats). Data are presented as mean values ± SEM and were analyzed with one-way ANOVA followed by Tukey's multiple comparison test (*p < 0.0001 compared to WT in the absence of SAM). **C** – Thermal activation profile (left) and the corresponding melting temperatures (right) of the studied pathogenic mutants significantly differential response compared to a full-length CBS WT (n=7 independent repeats). Data are presented as mean values ± SEM and were analyzed with one-way ANOVA followed by Tukey's multiple comparison test (*p values equal <0.0001 and 0.0005 comparing WT with P422L and Q526K CBS variants, respectively). **D** – Thermal stability profile (top) and the corresponding melting temperatures (bottom) in the absence and presence of 200 μM SAM showing significantly impaired thermal stability of the studied pathogenic CBS mutants compared to CBS WT (n=9 independent repeats). Data are presented as mean values ± SEM and were analyzed with one-way ANOVA followed by Tukey's multiple comparison test (*p values equal to <0.0001 except for CBS Q526K in the absence of SAM yielding a value of 0.0331 when compared to WT in the absence of SAM). **E** – Visualization of the affected residues within the RD-RD interface and cryoEM micrographs of the studied pathogenic mutants in the absence and presence of SAM. Red asterisk and hashtag denote *trans*-basal and *cis*-basal CBS filaments, respectively.

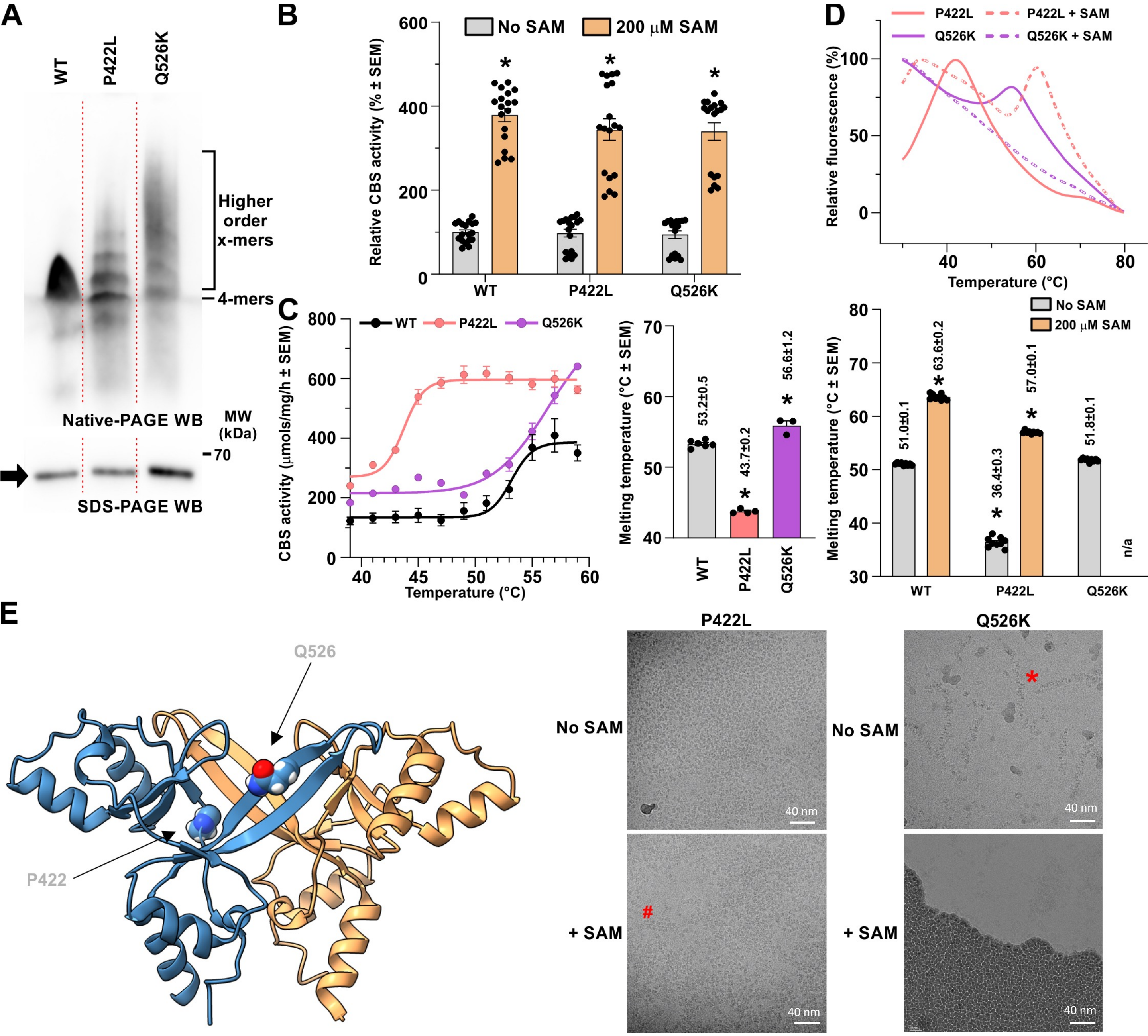

Supplementary figure 20

Supplementary figure 21. Morpheein-based assembly pathway for SAM-dependent CBS activation from *trans*-basal to *allo*-activated stacked filaments. A – Schematic of the ligand-free *trans*-basal filament (repeating *trans*-dimer in blue and orange) and the proposed SAM-induced dissociation step (**1**), generating a mixture of lower-order species, including *cis*-dimers (blue/orange) and less frequent *allo*-dimer intermediates (red/green). The *cis*-dimers can also self-associate to form the *cis*-basal filament (**2**). **B** – Representative *cis–allo* tetrameric intermediates (i and j), illustrating how *cis*-like peripheral dimers (blue/orange) can associate with an *allo*-dimer pair (red/green) to generate a stacking interface for nucleation. **C** – Formation of an activated octameric stack (**3**) consisting of two *cis*-dimers and two *allo*-dimers, proposed as the minimal seed for the *allo*-activated stacked filament. **D** – End-to-end elongation of stacked filaments by iterative addition of octameric units (**4**), requiring priming of the RD from the *cis*-/*allo*-tetramer (asterisked protomers, i* and j*, shown in green and red) to accommodate the next incoming assembly and drive bidirectional growth to generate the *allo*-activated stacked filament.

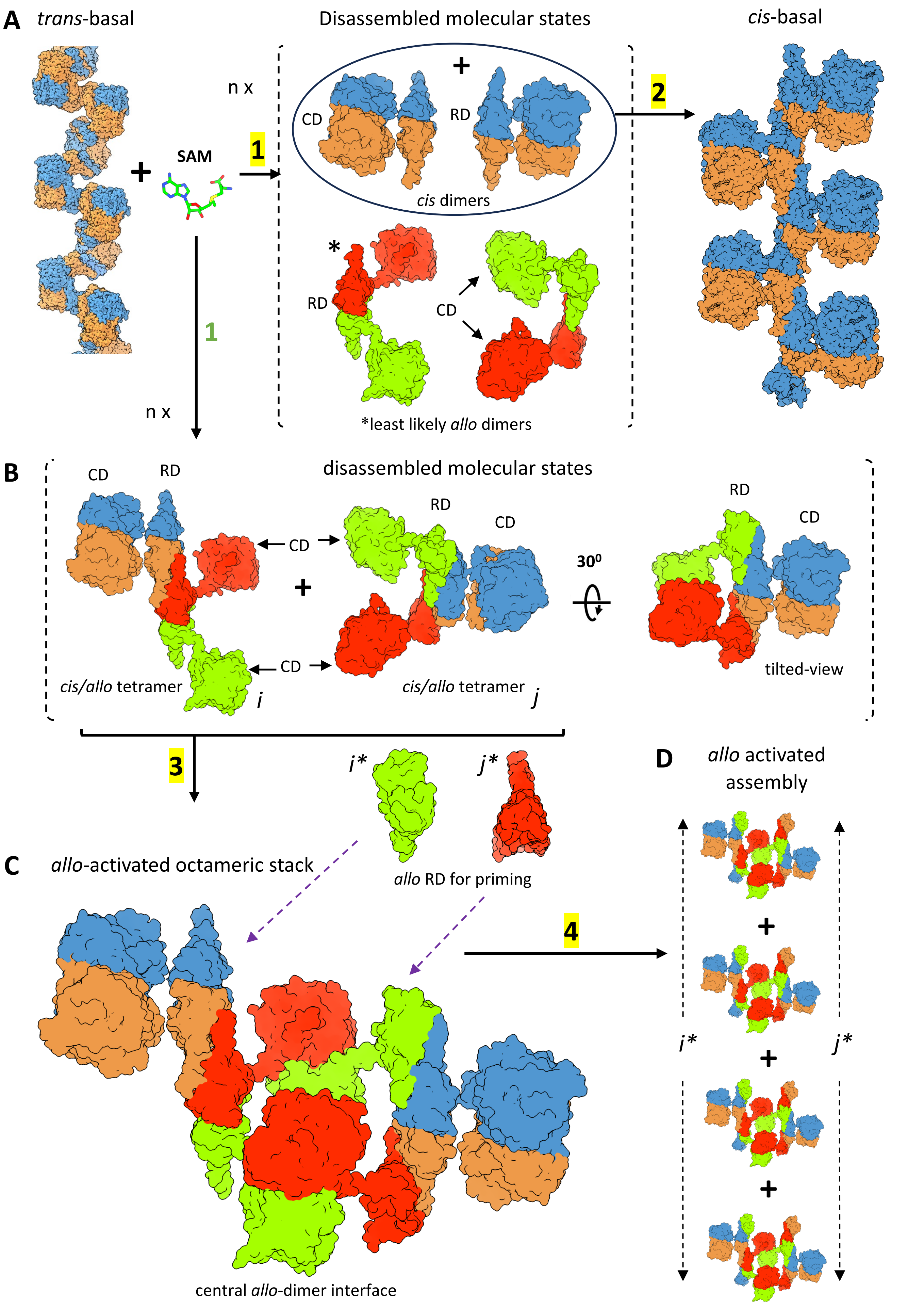

Supplementary figure 21

Supplementary figure 22. Sequence conservation of the oligomerization loop. Protein sequence alignment of a limited number of CBS enzymes focusing on the section of CBS2 domain within the regulatory domain, which harbors the oligomerization loop. The oligomerization loop 516-525 and residue Y518 of human CBS WT (isoform 1) are indicated by orange rectangle and red arrow, respectively. Protein sequences were taken from the Uniprot database as indicated by the accession ID in the name followed by abbreviations of species: *Homo sapiens* (HS), *Macaca fascicularis* (MF, macaque), *Gorilla gorilla* (GG, gorilla), *Oryctolagus cuniculus* (OC, rabbit), *Ratus norvegicus* (RN, rat), *Mus musculus* (MM, mouse), *Cavia porcellus* (CP, guineapig), *Xenopus laevis* (XL, frog), *Danio rerio* (DR, zebrafish), *Apis mellifera* (AM, honeybee), *Drosophila melanogaster* (DM, fruitfly), *Dictyostelium discoideum* (DD, slime mold), *Saccharomyces cerevisiae* (SC, yeast).

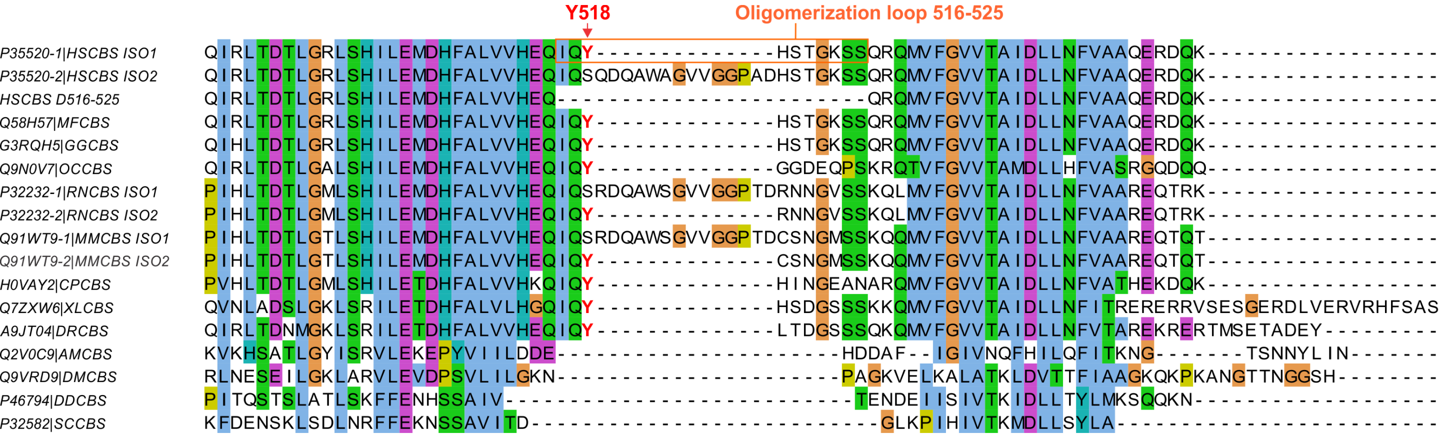

Supplement: Supplementary file 1 — Supplementary Information [file 41467_2026_73198_MOESM1_ESM.pdf]
