# Supplementary material for: Structural basis for a filamentous morpheein model of human cystathionine beta-synthase
Source: Nat Commun. 2026 Jun 6;17:7221. doi: 10.1038/s41467-026-73198-7 (PMC13396399; doi:10.1038/s41467-026-73198-7)
Supplement: Supplementary file 2 — Description of Additional Supplementary Files [file 41467_2026_73198_MOESM2_ESM.pdf]

Description of Additional Supplementary Files

Supplementary Movie 1. Conformational changes in human CBS associated with a catalytic cycle. Subtle changes within the filamentous structure of trans-basal CBS filament upon substrate binding and initiation of catalysis. Three catalytic intermediates were successfully captured, resolved and modeled into this morph movie: internal aldimine (CBS-Lys-PLP), serine-bound external aldimine (CBS PLP-Ser) and aminoacrylate intermediate (CBS PLP-AA). Movie depicts a segment consisting of three trans-dimer with individually colored subunits: orange-grey, green-yellow and pink-purple.

Supplementary Movie 2. Detail of the conformational changes in human CBS associated with a catalytic cycle. Zoomed in view into the central green-yellow trans-dimer showed in Supplementary movie 1.
