# Supplementary material for: Structural basis for a filamentous morpheein model of human cystathionine beta-synthase
Source: Nat Commun. 2026 Jun 6;17:7221. doi: 10.1038/s41467-026-73198-7 (PMC13396399; doi:10.1038/s41467-026-73198-7)
Supplement: Supplementary file 5 — Reporting Summary [file 41467_2026_73198_MOESM5_ESM.pdf]

nature portfolio

Corresponding author(s): Tomas Majtan and Inayathulla Mohammed

Last updated by author(s): Apr 20, 2026

Reporting Summary

Nature Portfolio wishes to improve the reproducibility of the work that we publish. This form provides structure for consistency and transparency in reporting. For further information on Nature Portfolio policies, see our Editorial Policies and the Editorial Policy Checklist.

Statistics

For all statistical analyses, confirm that the following items are present in the figure legend, table legend, main text, or Methods section.

n/a	Confirmed	
☐	☒	The exact sample size (*n*) for each experimental group/condition, given as a discrete number and unit of measurement
☐	☒	A statement on whether measurements were taken from distinct samples or whether the same sample was measured repeatedly
☐	☒	The statistical test(s) used AND whether they are one- or two-sided<br>*Only common tests should be described solely by name; describe more complex techniques in the Methods section.*
☒	☐	A description of all covariates tested
☒	☐	A description of any assumptions or corrections, such as tests of normality and adjustment for multiple comparisons
☐	☒	A full description of the statistical parameters including central tendency (e.g. means) or other basic estimates (e.g. regression coefficient) AND variation (e.g. standard deviation) or associated estimates of uncertainty (e.g. confidence intervals)
☐	☒	For null hypothesis testing, the test statistic (e.g. *F*, *t*, *r*) with confidence intervals, effect sizes, degrees of freedom and *P* value noted<br>*Give P values as exact values whenever suitable.*
☒	☐	For Bayesian analysis, information on the choice of priors and Markov chain Monte Carlo settings
☒	☐	For hierarchical and complex designs, identification of the appropriate level for tests and full reporting of outcomes
☒	☐	Estimates of effect sizes (e.g. Cohen's *d*, Pearson's *r*), indicating how they were calculated

Our web collection on statistics for biologists contains articles on many of the points above.

Software and code

Policy information about availability of computer code

Data collection	Dubochet Center for Imaging, Lausanne and LBEM in-house TITAN G4, Falcon 4 and Selectris (Thermo Fisher Scientific), EPU software v2.1<br>Fluorescent imaging was performed at UNIFR's Bioimage core facility using Leica Stellaris 8 Falcon operated by LAS X software and Agilent Cytation 5 operated by Gen5 software. Confocal images were processed using Imaris.<br>Activity assay data were recorded using Molecular Devices Spectramax M5 operated by Softmax Pro software.<br>Western blot chemiluminescence was captures using Azure Imagin System 300, while infrared signal was recorder using LICOR Odyssey CLx.
Data analysis	CryoSPARC v3.1.0/v4.4.0/v4.7.0, COOT v0.9.8.92, MOLREP CCP4 v8.0, UCSF Chimera v1.19, UCSF ChimeraX v1.10dev, PHENIX v1.1.4.5430dev, NAMDINATOR (no version available: https://github.com/namdinator/), MS Excel 365, GraphPad Prism v10.4.0, Imaris v9.7.1, ImageJ Fiji v1.54

For manuscripts utilizing custom algorithms or software that are central to the research but not yet described in published literature, software must be made available to editors and reviewers. We strongly encourage code deposition in a community repository (e.g. GitHub). See the Nature Portfolio guidelines for submitting code & software for further information.

Data

Policy information about availability of data

All manuscripts must include a data availability statement. This statement should provide the following information, where applicable:

- Accession codes, unique identifiers, or web links for publicly available datasets
- A description of any restrictions on data availability
- For clinical datasets or third party data, please ensure that the statement adheres to our policy

All cryo-EM density maps and corresponding atomic coordinates generated in this study have been deposited in the Electron Microscopy Data Bank (EMDB) and Protein Data Bank (PDB). The trans-basal CBS filaments in the absence of substrates or allosteric ligands are available under accession codes EMD-55115/PDB 9SQQ, EMD-55117/PDB 9SQU, and EMD-55105/PDB 9SQ0. Serine-bound trans-basal CBS is deposited as EMD-55037/PDB 9SML, EMD-54904/PDB 9SHM, EMD-54905/PDB 9SHN, and EMD-54925/PDB 9SI8. The SAO-bound cis-basal CBS filaments are deposited as EMD-55095/PDB 9SPT, EMD-55097/PDB 9SPV, EMD-55099/PDB 9SPW, and EMD-55102. The SAM-bound allo-activated stacked CBS filaments are deposited as EMD-55128/PDB 9SR3, EMD-55130/PDB 9SR4 and PDB 9SR7. In addition, SAM-bound cis-basal CBS filament is deposited as EMD-55132/PDB 6SR6 and EMD-55133. A complete list of all datasets and accession codes is provided in Supplementary table 1. Source data are provided within this paper.

Research involving human participants, their data, or biological material

Policy information about studies with human participants or human data. See also policy information about sex, gender (identity/presentation), and sexual orientation and race, ethnicity and racism.

Reporting on sex and gender	n/a
Reporting on race, ethnicity, or other socially relevant groupings	n/a
Population characteristics	n/a
Recruitment	n/a
Ethics oversight	n/a

Note that full information on the approval of the study protocol must also be provided in the manuscript.

Field-specific reporting

Please select the one below that is the best fit for your research. If you are not sure, read the appropriate sections before making your selection.

[x] Life sciences [ ] Behavioural & social sciences [ ] Ecological, evolutionary & environmental sciences

For a reference copy of the document with all sections, see nature.com/documents/nr-reporting-summary-flat.pdf

Life sciences study design

All studies must disclose on these points even when the disclosure is negative.

Sample size	Experimental sample sizes were established to achieve sufficient signal quality and statistical reliability for data interpretation. Details on the total micrographs acquired for cryo-EM analyses are presented in the Supplementary material. For biochemical assays, sample size for each experiment was represented by at least 3 replicates. For cell biology assays, at least 3 biological replicates were used.
Data exclusions	No data excluded. In some experiments, certain data may be missing due to a failure to obtain reasonable curve fit, which resulted in less points shown in the charts compared to technical/biological replicates generated.
Replication	For cell biology assays, at least 3 biological replicates. For biochemical assays, minimum of 3 technical replicates were used. Replication attempts were successful confirming that the reported data can be successfully replicated and reproduced.
Randomization	No randomization was implemented as it was not for structural, biochemical and cell biology studies reported in the manuscript.
Blinding	No blinding was implemented as it was not relevant for structural, biochemical and cell biology studies reported in the manuscript.

Reporting for specific materials, systems and methods

We require information from authors about some types of materials, experimental systems and methods used in many studies. Here, indicate whether each material, system or method listed is relevant to your study. If you are not sure if a list item applies to your research, read the appropriate section before selecting a response.

Materials & experimental systems

n/a	Involved in the study	
☐	☒	Antibodies
☐	☒	Eukaryotic cell lines
☒	☐	Palaeontology and archaeology
☒	☐	Animals and other organisms
☒	☐	Clinical data
☒	☐	Dual use research of concern
☒	☐	Plants

Methods

n/a	Involved in the study	
☒	☐	ChIP-seq
☒	☐	Flow cytometry
☒	☐	MRI-based neuroimaging

Antibodies

Antibodies used: anti-CBS (CST# 14782, 1:500 - 1:2,000), anti-rabbit AlexaFluorPlus 568 (Invitrogen# A-11011, 1:1,000), anti-beta-actin (Sigma# A1978, 1:5,000), anti-rabbit IgG HRP (CST# 7074, 1:5,000), anti-mouse IgG HRP (CST# 7076, 1:5,000)

Validation: validated by the commercial manufacturer...see the product website for details.

Eukaryotic cell lines

Policy information about cell lines and Sex and Gender in Research

Cell line source(s): HEK293A (Invitrogen# R70507) and its genetic modifications reported in the manuscript

Authentication: authenticated by the commercial manufacturer...see the product website for details.

Mycoplasma contamination: All cell lines were negative for mycoplasma contamination

Commonly misidentified lines (See ICLAC register): used HEK293A line is different from ICLAC-00063

Plants

Seed stocks: n/a

Novel plant genotypes: n/a

Authentication: n/a
