# Supplementary material for: Structural basis for a filamentous morpheein model of human cystathionine beta-synthase
Source: Nat Commun. 2026 Jun 6;17:7221. doi: 10.1038/s41467-026-73198-7 (PMC13396399; doi:10.1038/s41467-026-73198-7)
Supplement: Supplementary file 6 — Transparent Peer Review File [file 41467_2026_73198_MOESM6_ESM.pdf]

nature portfolio

Peer Review File

Structural basis for a filamentous morpheein model of human cystathionine beta-synthase

Corresponding Author: Dr Tomas Majtan

This file contains all reviewer reports in order by version, followed by all author rebuttals in order by version.

Version 0:

Reviewer comments:

Reviewer #1

(Remarks to the Author)
In this manuscript, Inayathulla Mohammed., et al determined three distinct cryo-EM structures of filamentous human cystathionine beta-synthase (CBS), which are (1) ligand-free trans-basal filaments ,(2) adenosylornithine (SAO)-bound cis-basal filaments and (3) S-adenosylmethionine (SAM)-bound allo-activated stacked filaments. These structures revealed human CBS is a filamentous morpheein, it undergoes significant conformational changes in the absence or presence of different allosteric ligands. They showed these distinct filamentous CBS can transition reversibly through disassociation and subsequent re-assembly. The authors advance our understanding of CBS structure-function relationships through linking CBS distinct filamentous states to its enzyme stability, activity and cellular turnover by performing biochemistry, molecular biology and confocal imaging experiments. Through carefully analyzing their structural, biological and cellular data and extensive rational discussion, they proposed a morpheein-based model to dissect the CBS oligomerization and allosteric regulation.
They also determined cryo-EM structures of the pyridoxal-5’-phosphate(PLP)-bound CBS filaments in both the absence or presence of its substrate, L-serine. These structures uncovered the molecular mechanism of CBS catalysis and shed lights on the conformational flexibility of filamentous CBS in the active sites.
In summary, all of these novel findings in this manuscript will advance our understanding on the functions of distinct filamentous CBS and offer us new ways therapeutically modulating CBS function to cure diseases. This is a pretty novel study. The manuscript is relatively well written, although I still have some concerns regarding to this manuscript. I recommend accepting this manuscript for publication on Nature Communications after addressing my following concerns.

Major Comments:
1. In the introduction, the authors introduced the methionine metabolism pathway. I recommend the authors draw a visualized schematic figure to show this pathway and highlight the key steps that regulated by cystathionine beta-synthase. It will make the readers easily understand this pathway.

2. How do the authors think about the balance between the increased stability by filamentation and conformational plasticity of CBS? Does filamentation of CBS increase the rigidity and reduce the conformational plasticity of CBS? Could author answer this question?

3. In this manuscript, the authors think that SAM triggers CBS activation through stacked filamentation, which will enhance its enzymatic activity by stabilizing the protein within the cellular environment. Have the authors checked the active site of SAM-bound allo-activated stacked CBS filament? Could the authors observe the induced conformational changes in the active site by SAM binding in SAM-bound allo-activated stacked CBS filament? Although I noticed that the cryo-EM map of SAM-bound allo-activated stacked CBS filament is low resolution.

Minor comments:
In text lines 490-491, the authors claimed that basal state of CBS filament in variable length. This conclusion is not so convincible in figure 1B. They should provide some 2D classes of CBS with distinct difference in length.

In text lines 502-509, the authors mentioned “Helix α15”, but they don’t provide details about how they name this helix. A sophisticated figure is required to show these details, as well as details of the interaction interface.

Figure citation in text line 569 is not correct. Please cite figure 4E in text line 569.

Please correct the text line 625 as “ Each stacked unit is composed of two cis-dimers”.

There is a grammatical mistake in the text line 941 in the legend of figure 1D. please correct it as “ Atomic model details (or shows)…”

Figure issues:
In figure 1.
1. In figure 1A. The N-terminal domain should include residues 1-70 as mentioned in text, but the bi-directional arrow points residues 1-40 indicating N-terminal domain in figure 1A. Please correct it.
2. In figure 1A. The evolutionary conserved residue in catalytic domain is K199 in text, but K119 is indicated in figure 1A. It’s confusing, please correct it.
3. In figure 1C. Please indicate and show the rise and twist in the figure 1C. I also recommend to remove the inset in figure 1C, it’s redundant.
4. In figure 1D. The label “Helix α15” in right bottom panel is not mentioned before. If it’s first time to mention this helix in their figures, the author should show reader how they name this helix.
5. In figure 1D. The description for interaction mediated by oligomerization loops in this figure legend is not detailed and precise, but they described these interactions pretty well in their text lines 502-510. I recommend that they re-make figure 1D to show the interaction details between oligomerization loops that they mentioned in main text and modify this figure legend.

In figure 3.
1. Please indicate and label the serine in the CBS PLP-Ser intermediate in right panel of figure 3B. It will clearly show readers where the serine is covalently bound to PLP.
2. Could the authors label the residues which involve the chemical reaction events in the active sites? It’s meaningful to discuss how the conformational changes of these residues would alter the compaction of active site in the main text.
3. The authors mentioned the compaction of active site when transition from CBS-Lys-PLP to CBS PLP-AA. Could they figure out a way to show this compaction in figure 3?

In figure 4.
1. In figure 4A. Please provide high-resolution molecular formula of SAM,SAH and SAO.
2. In figure 4D. I recommend to remove inset figure from figure 4D. It’s redundant. The 2D classes in figure 4C already shows the right-handed helical geometry for SAO-bound CBS.
3. Please correct the text line 995 as “ their CDs forming disc-shaped CBS module reside atop of their respective RDs.”
4. I recommend to move figure 4F up and figure 4E down. So it will make a smooth transition from figure 4F to 4G.
5. Please specify what represents by different color codes in the legend of figure 4H, in order to let readers interpret the electrostatic surface potential map here.

Reviewer #2

(Remarks to the Author)
Mohammed and colleagues present high-resolution cryo-EM structures of human CBS and propose a SAM-mediated, morpheein-type activation mechanism that includes a novel "allo-activated stacked filament" conformation. The manuscript is original, technically sound, and makes an important contribution to sulfur metabolism and enzyme regulation. I have nonetheless several concerns that should be addressed:
1. Strengthen experimental support for the proposed mechanistic model so it moves beyond a well-argued hypothesis to a demonstrably tested mechanism by, for instance, targeted mutagenesis and in vitro functional assays, i.e. design mutations that disrupt the key interfaces stabilizing the allo-activated stacked filament and test their functional effects.
2. Indicate whether any described pathogenic variants map to the newly identified filament interfaces and, if so, test and discuss functional implications.
3. Improve connection between the in vitro structural observations and physiological relevance by adding cellular evidence of the effects, i.e. express selected interface mutants in a cellular model and measure CBS activity and cellular redox markers (for example H2S production, glutathione/redox reporter assays, or other appropriate readouts). Where direct CBS readouts are difficult, use complementary assays that reflect downstream metabolic consequences to link structural state to cell physiology.
4. Make clearer and more explicit comparisons to prior structural and functional work (for example McCorvie et al., Nat Commun 2024) and highlight what is truly novel about the filament conformation reported here. For instance, i) the filaments described in the first section of Results are identical to the previously published ones or differ in some aspect? ii) There is no mention to previously published results showing stabilization of CBS filaments in vitro in the second section of Results.
5. Include structural comparisons/superimpositions of the different structures to facilitate visualization of the conformational changes in the dimers/tetramers to help to interpret details of the proposed morpheein-type model in Figure 7.
6. Include more recent and relevant references on enzyme filamentation and its functional/regulatory roles in both Results and Discussion to frame the findings.

Reviewer #3

(Remarks to the Author)
The manuscript entitled “Structural basis for a filamentous morpheein model of human cystathionine beta-synthase”, by

Inayathulla Mohammed and co-workers, reports on compelling data that provide a structural basis for human CBS dynamic oligomerization and allosteric activation mechanisms. The results herein described, from a solid combination of biochemical, cell-based assays and cryoEM data are highly relevant for the understanding of how this human enzyme is regulated, with implications in various aspects of physiology and pathophysiology.
Although I feel unqualified to adequately judge the cryoEM data collection, processing and refinement, I carefully revised all other experimental methods and data, as well as the discussion on possible mechanistic and (patho)physiological implications of these results.

I find the manuscript extremely clear and well written and the data well presented, but I request the following points to be addressed:

1. The first ~40 amino acids of full-length CBS have never been observed in previously reported structural data, both crystallographic and cryoEM data. This intrinsically disordered region has been shown, by NMR spectroscopy, to be able to accommodate a b-type heme (Kd ~2µM), with cysteine and histidine ligands, in a cysteine-proline motif [https://doi.org/10.1038/s41598-018-20841-z]. However, the function of this region and the relevance of its heme-binding ability remains unknown. Since the deposited PDB files are still pending publication, I could not figure out the sequence coverage of each structure herein reported and thereby understand whether these structures contain any information about this intrinsically disordered region. The only mention I could find of it in the manuscript is the term IDR in Figure 1A. So, my point is that, since it is indeed a structural element of CBS, it should be mentioned in the manuscript whether there is any density indicative of its position, and if in the presented models of the different oligomeric arrangements it is likely this domain could participate in any interaction between protomers (from the presented structures, it seems unlikely, but I think the reader should be given that information).

2. A minor technical point: on page 5, line 157, it is stated that to obtain the trans-basal CBS dataset, the sample was diluted in a buffer containing 1 mM β-mercaptoethanol. What was the reason for that? And was β-mercaptoethanol also added to the samples to obtain the other datasets? (If so, it should be mentioned). This point is further expanded in comment 7.4.

3. On page 10, line 349, it is mentioned that the ligands SAM, PLP and serine were manually docked in Coot into corresponding densities. Then, Supplementary table 1 mentions the presence of heme ligands. Was there density for the heme ligands (to what extent, i.e., always 1 heme per monomer?), or were the hemes added on between the Cys52 and His65 axial ligands? Please clarify in the manuscript.

4. On page 14, lines 525-527, and supported by Figure 1D, Y518 is singled out as being crucial for the described assemblies, by inserting into a hydrophobic pocket. I think it should be mentioned in the Results and/or Discussion the degree of sequence conservation of this residue across species. Lines 720-722 clarify that this filamentation ability may be species-specific, but assigns the lack of filamentation in mouse CBS to a longer oligomerization loop (similarly to human ISO2 CBS). It could be interesting to assess the conservation of this Y518 residue (and the shorter oligomerization loop), which should in principle allow predicting which CBS from which species can adopt this filamentous assembly.

5. From Figure 7, I am unable to perceive whether the Authors were able to find density for SAM in the core allo-dimers. On Supplementary Table 1, SAM was only modelled in 9SR6, but it's unclear whether the data provide evidence for SAM binding to both cis- and allo-dimers. I understand that the resolution for this arrangement may preclude obtaining unequivocal densities for SAM, but this should anyway be clarified in the manuscript.

6. Still related with the previous comment, I think the Authors can slightly improve the clarity of Figure 7 in what concerns the bottom-right part of the figure, where allo-activated stacked CBS filaments are depicted. As it is, it is not immediately clear to which protomer each CD and RD from the core allo-dimers belong. So, as in Figure 5D, the Authors could simply keep one dark red + dark green protomer and make the other protomer pink (light red) and light green. This should make it clearer both in the octameric stack and in the filament.

7. CBS is a fascinating enzyme, from different viewpoints, regarding its structure, function and regulation. I think the Authors could enrich the Discussion by explaining how these filamentous assemblies reconcile with previous data on CBS regulation:
7.1. In comment 1, I already alluded to the N-terminal IDR which should be mentioned.
7.2. Concerning the heme moiety, it has been shown, mostly by Ruma Banerjee's group, to regulate CBS activity through its redox and ligand state [PMID: 18278872, 19232736, 19733148, 22977242, 23790103, 24416422, 26867575, 27365395, 16505479, 15581573]. We have further demonstrated that SAM binding renders CBS more prone to become inactivated by CO and NO [https://doi.org/10.1074/jbc.m115.681221]. This allosteric regulatory mechanism has been proposed to be mediated by a helix spanning from the heme 'surroundings' to the PLP active site, with emphasis on Arg266 that is H-bonded to the heme ligand Cys52 [https://doi.org/10.1074/jbc.m112.414706].
It would be useful to perceive how the heme-binding region and the Thr257-Arg266 helical segment in these filamentous CBS assemblies (basal SAM-free and SAM-bound) are compatible with the heme-based regulation.
7.4. Along the same lines, Niu and co-workers have demonstrated allosteric control of CBS activity by a surface exposed disulfide bond between Cys272 and Cys275 [https://doi.org/10.1074/jbc.ra117.000103]. Since the Authors used 1 mM β-mercaptoethanol in their sample preparation, this has likely resulted in reduction of the 'resting' disulfide bond and therefore prompted an already 2-3-fold pre-activated CBS. In light of this, it would be interesting to disclose the position of these cysteine residues in the obtained structures, particularly those with higher resolution, and comment on how the filamentous CBS assemblies still allow for this additional regulatory switch.
7.5. CBS has been shown to accumulate in mitochondria, in a process mediated by Lon protease [

https://doi.org/10.1073/pnas.1308487110]. Herein, the Authors show accumulation of CBS filaments close to the nucleus. I know it may be a stretch to speculate on that, but it would be interesting to discuss how the filamentous CBS data can accommodate this re-localization phenomenon.

Version 1:

Reviewer comments:

Reviewer #1

(Remarks to the Author)
I would like to give the authors a good credit since they did a good job in this revised manuscript. Not only did they address my questions and concerns in the previous manuscript carefully and clearly, but they also refined the figures to make them look much better and easily interpretable.

In this revised manuscript, which got my attention and is more interesting is that they tested two pathogenic human cystathionine beta-synthase (CBS) P422L and Q526K variants. Through performing different assays (native gel assays, thermal stability assays), they would be able to apply their morpheein-based model of CBS regulation to understand how these variants could lead to diseases. Build on previous findings, their study revealed a more complex regulation mechanism of CBS and shed light on new ways therapeutically modulating CBS function to cure diseases.

In my opinion, the results in this manuscript have been interpreted properly and the manuscript is well written after revision. I recommend accepting this manuscript for publication on Nature Communications. But one more suggestion is provided below for consideration.

Figure issue:
For figure 1. It’s great that the authors made figure 1 to illustrate the methionine metabolism pathway. I like it. But I recommend the authors could move the figure 1 to the supplemental figures since figure 1 doesn’t provide new data. And this could reduce the main figure number too.

Reviewer #2

(Remarks to the Author)
I appreciate that the authors have addressed the concerns raised in my previous review. I now only have a few minor suggestions aimed at improving the clarity and readability of the manuscript.

First, while the placement of the section "Substrate binding reveals catalytic rearrangements of the active site" is ultimately at the authors’ discretion, I find that it interrupts the flow of the Results section and detracts from the main message. I would recommend moving it to either the beginning or the end of the Results section to improve clarity and coherence.

Second, it would be helpful to clarify in the main text how SAM-induced structural rearrangements result in the His-tag becoming buried or otherwise inaccessible for interaction with the Ni-NTA matrix.

Finally, the statement regarding the anticancer drug taxol could be refined for accuracy and clarity. Taxol is known to bind polymerized microtubules rather than dimeric tubulin, and it stabilizes microtubules rather than destabilizing them.

Reviewer #3

(Remarks to the Author)
The Authors appropriately addressed the comments I had raised, and performed elegant complementary experiments to address comments by the other reviewers which nicely supported the proposed models for oligomerization. In my opinion, the manuscript can be accepted for publication.

I have one minor suggestion:
On lines 67, 1307 and 1321 of the revised manuscript (and the new Figure 1), I suggest adding ‘reverse’ before ‘transsulfuration pathway’, in line with what should be the formal designation of the mammalian transsulfuration pathway converting methionine to cysteine.

Open Access This Peer Review File is licensed under a Creative Commons Attribution 4.0 International License, which permits use, sharing, adaptation, distribution and reproduction in any medium or format, as long as you give appropriate credit to the original author(s) and the source, provide a link to the Creative Commons license, and indicate if changes were made.
In cases where reviewers are anonymous, credit should be given to 'Anonymous Referee' and the source.
The images or other third party material in this Peer Review File are included in the article's Creative Commons license, unless indicated otherwise in a credit line to the material. If material is not included in the article's Creative Commons license and your intended use is not permitted by statutory regulation or exceeds the permitted use, you will need to obtain permission directly from the copyright holder.
To view a copy of this license, visit https://creativecommons.org/licenses/by/4.0/

Response to the reviews of the manuscript ID# NCOMMS-25-87465-T:

Dear Colleagues,

Thank you for your thorough evaluation of our manuscript and for the constructive comments and suggestions. Below please find a point-by-point response to each of your specific comments. We have revised the manuscript as outlined in our response. All the revisions and changes are highlighted in a tracked copy of our revised manuscript alongside a clean revised manuscript.

We hope that you will find our revision in response to your reviews acceptable and that you will be able to recommend the revised manuscript for acceptance to the Editor.

Response to the **Reviewer #1** comments:

Major comments:

1. ***In the introduction, the authors introduced the methionine metabolism pathway. I recommend the authors draw a visualized schematic figure to show this pathway and highlight the key steps that are regulated by cystathionine beta-synthase. It will make the readers easily understand this pathway.***

We added Figure 1 showing schematics of sulfur amino acid metabolism pathways and highlighting the key role of CBS in diverting sulfur from the methionine cycle toward cysteine synthesis as well as how SAM regulates organic sulfur flux through the competing methionine cycle and transsulfuration pathway.

2. ***How do the authors think about the balance between the increased stability by filamentation and conformational plasticity of CBS? Does filamentation of CBS increase the rigidity and reduce the conformational plasticity of CBS? Could author answer this question?***

This is an important and nuanced question. Based on our structural and biochemical data, we do not view CBS filamentation as a simple rigid "lock" that suppresses conformational dynamics. Rather, we propose that CBS filaments provide a stabilizing scaffold while still permitting substantial local flexibility required for catalysis and regulation. In particular, CBS must retain conformational responsiveness to physiological cues, such as availability of substrates and changes in intracellular SAM concentrations, and our data support the view that this responsiveness is preserved within filamentous assemblies.

Our observations suggest that stabilization is not uniformly distributed across the protein. The regulatory domain (RD) is primarily stabilized by ligand binding, especially SAM, through formation of the CBS module, whereas filamentation mainly contributes to the stability at the level of quaternary organization (i.e. by promoting higher-order assembly and consequently reducing cellular turnover). In contrast, conformational plasticity appears most relevant to the catalytic core, including the catalytic domain and the N-terminal heme-binding region. Although the resolution of the stacked *allo*-activated assemblies limits detailed interpretation of catalytic site rearrangements, our model predicts that the catalytic cores within the central *allo*-dimers retain (and may even require) enhanced flexibility, consistent with their proposed role in supporting increased catalytic turnover upon SAM stimulation.

Functionally, filamentation modestly enhances stability in the presence of non-activating ligands SAO or SAH (i.e. by forming *cis*-basal filament from the *trans*-basal assembly), whereas SAM-induced stacking produces a markedly more stable *allo*-activated stacked filamentous state. Thus, we suggest that CBS achieves regulation through a balance in which higher-order assembly increases protein cellular stability and robustness, while local catalytic dynamics remain sufficiently flexible to support chemistry and allosteric transitions.

Finally, although the intrinsically disordered N-terminus (first ~40 residues) is not resolved in our structures or in any prior CBS structural work, we note that it remains plausible that this segment could contribute to the stabilization of *allo*-activated stacked assemblies, e.g. by transient crosslinking or inter-stack contacts. We present this only as a hypothesis and have framed it accordingly in the revised manuscript.

3. ***In this manuscript, the authors think that SAM triggers CBS activation through stacked filamentation, which will enhance its enzymatic activity by stabilizing the protein within the cellular environment. Have the authors***

checked the active site of SAM-bound allo-activated stacked CBS filament? Could the authors observe the induced conformational changes in the active site by SAM binding in SAM-bound allo-activated stacked CBS filament? Although I noticed that the cryo-EM map of SAM-bound allo-activated stacked CBS filament is low resolution.

Yes, we examined the active site of the SAM-bound *allo*-activated stacked CBS filament, as this is central to our proposed activation model. Efforts to improve the structural interpretability of the stacked core; particularly, the central *allo*-CBS dimers involved extensive optimization and analysis. During this substantial delay another group (Dr. McCorvie and Prof. Yue) reported CBS filamentation. After nearly two years focused on resolving this region, we present here the best reconstructions we were able to obtain. However, the resolution in the central stacked catalytic core remains insufficient to reliably model active site features or to unambiguously identify SAM-induced conformational changes within the catalytic pocket. We have clarified this limitation in the revised manuscript and therefore base our mechanistic conclusions primarily on the distinct quaternary architecture of the stacked assembly and its correlation with maximal enzymatic activation.

<u>Minor comments:</u>

1. ***In text lines 490-491, the authors claimed that basal state of CBS filament in variable length. This conclusion is not so convincible in figure 1B. They should provide some 2D classes of CBS with distinct differences in length.***

   Because long CBS filaments require large extraction boxes (up to 2048 pixels), 2D classification often included neighboring filaments, which limits clean separation of all length variants into distinct classes. Nonetheless, we identified representative 2D class averages showing clear differences in filament length and now highlight them in the manuscript. These additions are now provided in Figure 2B and support our conclusion that basal CBS filaments exhibit variable lengths.

2. ***In text lines 502-509, the authors mentioned "Helix α15", but they don't provide details about how they name this helix. A sophisticated figure is required to show these details, as well as details of the interaction interface.***

   The numbering of secondary structure elements has been previously established when we resolved crystal structure of the engineered human CBS construct lacking the oligomerization loop 516-525 (<u>https://doi.org/10.1073/pnas.1313683110</u>) and followed in the field since then (e.g. <u>https://doi.org/10.1073/pnas.1414545111</u>, <u>https://doi.org/10.1074/jbc.m114.610782</u> or <u>https://doi.org/10.1038/s41467-024-46864-x</u>). We now show the requested sophisticated figure with details about the CBS secondary structures and their designation in Supplementary figure 1.

3. ***Figure citation in text line 569 is not correct. Please cite figure 4E in text line 569.***

   Both Figure 1E as well as Figure 4E show the CD-RD connecting linker: 1E in the *trans*-basal CBS filament, while 4E in the *cis*-basal CBS filament. Since both are correct, we added a reference to Figure 4E.

4. ***Please correct the text line 625 as "Each stacked unit is composed of two cis-dimers".***

   Corrected.

5. ***There is a grammatical mistake in the text line 941 in the legend of figure 1D. please correct it as "Atomic model details (or shows)…".***

   Corrected.

<u>Figure issues:</u>

In Figure 1:

1. ***In Figure 1A. The N-terminal domain should include residues 1-70 as mentioned in text, but the bi-directional arrow points residues 1-40 indicating N-terminal domain in figure 1A. Please correct it.***

   Corrected.

2. ***In Figure 1A. The evolutionary conserved residue in catalytic domain is K199 in text, but K119 is indicated in figure 1A. It's confusing, please correct it.***

   Corrected. It was a typo in the manuscript text.

3. ***In Figure 1C. Please indicate and show the rise and twist in the figure 1C. I also recommend to remove the inset in figure 1C, it's redundant.***

Both the rise and the twist have been mentioned within the figure, but now we re-drew it to indicate it better, while removing the inset at the same time.

4. ***In Figure 1D. The label "Helix α15" in right bottom panel is not mentioned before. If it's first time to mention this helix in their figures, the author should show reader how they name this helix.***

We have already addressed the secondary structure elements' numbering issue in the minor comment #2 above.

5. ***In Figure 1D. The description for interaction mediated by oligomerization loops in this figure legend is not detailed and precise, but they described these interactions pretty well in their text lines 502-510. I recommend that they re-make figure 1D to show the interaction details between oligomerization loops that they mentioned in main text and modify this figure legend.***

The secondary structure nomenclature, including helix α15, follows the convention established in our previously reported crystal structure of human CBS lacking the oligomerization loop (CBSΔ516–525). To improve clarity, we have revised Figure 2D to explicitly depict the interaction interface mediated by the oligomerization loop, highlighting contacts between the CBS2 motif of one subunit and the CBS1 motif (including helix α15) of the opposing subunit. The corresponding figure legend has been expanded to precisely describe these interactions, in line with the main text.

In Figure 3:

1. ***Please indicate and label the serine in the CBS PLP-Ser intermediate in right panel of figure 3B. It will clearly show readers where the serine is covalently bound to PLP.***

Updated. We did the similar for panel C and labeled aminoacrylate (AA) intermediate.

2. ***Could the authors label the residues which involve the chemical reaction events in the active sites? It's meaningful to discuss how the conformational changes of these residues would alter the compaction of active site in the main text.***

We have now explicitly labeled the residues directly involved in PLP chemistry and substrate/intermediate stabilization in the active site across the three trapped states (internal aldimine, external aldimine, and aminoacrylate intermediate), and provided the corresponding description in the Results section.

3. ***The authors mentioned the compaction of active site when transition from CBS-Lys-PLP to CBS PLP-AA. Could they figure out a way to show this compaction in figure 3?***

This is difficult, perhaps impossible task to show this dynamic process in a figure. The compaction is relatively small and occurs at the level of the entire catalytic core including the active site. For that reason, we provided new Supplementary movies illustrating these subtle changes within the filamentous structures, now using atomic models rather than the electron densities. In addition, we generated LigPlot+ interaction diagrams for each of the intermediates showing the interacting residues, their movement and compaction of the active site in Supplementary figure 9. They should assist in the explanation of how coordinated movements of these residues contribute to 'active-site compaction' during catalysis.

In Figure 4:

1. ***In Figure 4A. Please provide high-resolution molecular formula of SAM, SAH and SAO.***

Updated resolution of the molecular formulas/structures of SAM analogs.

2. ***In figure 4D. I recommend to remove inset figure from figure 4D. It's redundant. The 2D classes in figure 4C already show the right-handed helical geometry for SAO-bound CBS.***

The inset removed.

3. ***Please correct the text line 995 as "their CDs forming disc-shaped CBS module reside atop of their respective RDs."***

The original wording was correct, because it is two regulatory domains (RDs) from two CBS subunits forming a dimer, which in the presence of a ligand (e.g. SAO) form a disk-like structure called CBS module. This CBS module sits on top of the catalytic domain (CD) dimer in the *cis*-dimer. Furthermore, the presence of the oligomerization loop mediates multimerization of RDs' CBS modules forming a *cis*-basal filament with a central spindle decorated with CDs alternating from both of its sides.

4. ***I recommend to move figure 4F up and figure 4E down. So it will make a smooth transition from figure 4F to 4G.***

Reorganized as requested.

5. ***Please specify what represents by different color codes in the legend of figure 4H, in order to let readers interpret the electrostatic surface potential map here.***

Specified in the legend.

Response to the **Reviewer #2** comments:

1. ***Strengthen experimental support for the proposed mechanistic model so it moves beyond a well-argued hypothesis to a demonstrably tested mechanism by, for instance, targeted mutagenesis and in vitro functional assays, i.e. design mutations that disrupt the key interfaces stabilizing the allo-activated stacked filament and test their functional effects.***

We agree with the reviewer that such a characterization of specifically-designed CBS mutants targeting the interfaces involved in CBS filamentation could be helpful to further support our proposed model. At the same time, we feel that our data on different CBS isoforms already show such a support. Specifically, the CBS ISO2 with the extended oligomerization loop clearly does not form filaments similarly to CBSΔ516-525 with the oligomerization loop entirely missing, while both also displayed similar cellular half-life, which was significantly lower compared to CBS WT (ISO1) with the oligomerization loop intact. As CBS ISO2 is physiologically relevant and engineered CBSΔ516-525 has been previously biochemically and structurally characterized, we are convinced that these variants already offer relevant and sufficient support for our proposed model.

However, we decided to address the reviewer's suggestion by generating and characterizing an additional set of rationally designed variants with results shown now in Supplementary figures 4 and 5. Using two amino acid residue-longer and shorter oligomerization loop variants compared to the original CBSΔ516-525 (i.e. CBSΔ515-522 and CBSΔ515-526) as well as point mutants of key conserved residues (i.e. CBS I516G, CBS Q517G, CBS Y518G single mutants along with a CBS I516G+Q517G+Y518G triple mutant), we now clearly show that all these perturbation of the oligomerization loop interface yield dimeric, SAM-responsive CBS enzymes compared to tetrameric/filamentous CBS WT. Furthermore, thermal activation assays show lower stability of these dimeric forms compared to the filamentous CBS WT. Lastly, the cellular turnover of these dimeric forms is significantly reduced compared to CBS WT. Thus, we believe that the proposed model is now sufficiently supported by both the previously presented data and the additional experimental data.

2. ***Indicate whether any described pathogenic variants map to the newly identified filament interfaces and, if so, test and discuss functional implications.***

As our manuscript reports "only" structures of full-length CBS WT in the absence and presence of allosteric ligands, our idea was to focus on oligomerization and regulation of CBS under physiological conditions. Therefore, we tried to stay away from HCU-causing pathogenic mutants and discussing in detail structural and functional consequences due to the presence of pathogenic missense mutations. However, per reviewer's request, we now show in Supplementary figure 20 additional data related to HCU. Specifically, we generated and characterized two HCU-causing pathogenic missense mutants (CBS P422L and CBS Q526K), which map within the RD/oligomerization interface. Together, these new results show that the pathogenic variants affecting RD interfaces and the oligomerization loop can bias CBS towards lower-order oligomers, promote aggregation, and/or selectively impair the SAM-dependent transition to the *allo*-activated stacked assembly. We have incorporated these results into the revised manuscript and discuss their implications for CBS regulation.

3. ***Improve connection between the in vitro structural observations and physiological relevance by adding cellular evidence of the effects, i.e. express selected interface mutants in a cellular model and measure CBS activity and cellular redox markers (for example H2S production, glutathione/redox reporter assays, or other appropriate readouts). Where direct CBS readouts are difficult, use complementary assays that reflect downstream metabolic consequences to link structural state to cell physiology.***

Another great suggestion, although we believe we already provided sufficient cellular/functional evidence of the impact of CBS filamentation. First, we showed that non-filamentous CBS variants (CBS ISO2, CBSΔ516-525, CBS45) have faster cellular turnover compared to the filamentous CBS ISO1 (WT) (Figure 3F). Second, we showed that filamentous CBS ISO1 localizes into a ring-shaped structure surrounding the nucleus, while non-filamentous CBS forms were more or less homogenously distributed throughout the entire cytoplasm (Figure 3G). Third, we showed that methionine supplementation leading to increased cellular SAM levels resulted in a significant stabilization of endogenous CBS in HEP3B cells characterized by substantially slower turnover, while the decrease of cellular SAM levels by MAT inhibitor treatment resulted in a significant CBS destabilization and faster turnover compared to control conditions (Figure 7C). In our view, these studies already convincingly show a connection between the CBS filamentation state and cellular physiology.

However, we followed the reviewer's suggestion about the expression and characterization of selected interface mutants. Indeed, we now provide further cellular evidence that disruption of CBS filamentation by targeted mutagenesis of key interface residues (e.g. I516, Q517 and Y518) results in faster cellular turnover of CBS (Supplementary figure 5D). We also tried to measure cellular redox markers or metabolic consequences; however, we were unsuccessful to observe any differences. This could be due to the constitutive high-level expression from the CMV promoter, retained catalytic competency and regulation by the studied mutants, cellular redundancy of H_2S biogenesis pathways and high intracellular levels of the key downstream metabolite (glutathione) effectively interfering with the detection of subtle significant changes in its levels.

4. ***Make clearer and more explicit comparisons to prior structural and functional work (for example McCorvie et al., Nat Commun 2024) and highlight what is truly novel about the filament conformation reported here. For instance, i) the filaments described in the first section of Results are identical to the previously published ones or differ in some aspect? ii) There is no mention to previously published results showing stabilization of CBS filaments in vitro in the second section of Results.***

We did this in the Discussion and not in the Results as that is the more appropriate way to contrast and discuss our results with the previously published studies. We added a bit more explicit comparison to clearly differentiate our data and the proposed CBS oligomerization paradigm-shifting model as well as the cellular impact of CBS filamentation and stabilization from the previously published McCorvie et al. report.

5. ***Include structural comparisons/superimpositions of the different structures to facilitate visualization of the conformational changes in the dimers/tetramers to help to interpret details of the proposed morpheein-type model in Figure 7.***

To facilitate the visualization of the conformational transitions underlying our proposed morpheein-type model (now Figure 8), we have added two new Supplementary figures that provide explicit structural comparisons and an assembly schematic. Supplementary figure 19 presents a series of superpositions (*trans*-basal, SAO-bound *cis*-basal, SAM-bound *cis*-basal and central *allo*-state protomers/dimers) using either the CD or the RD-loop region as alignment references, thereby quantifying how the transitions are dominated by rigid-body repositioning of the RDs relative to an essentially conserved CDs, and highlighting the large *cis*-to-*allo* rearrangement. This figure also emphasizes the CD-RD connecting linker as a plausible hinge coordinating CD–RD reorientation during assembly switching. In parallel, Supplementary figure 21 provides a stepwise schematic that connects these structural differences to the morpheein mechanism, illustrating the proposed dissociation of the *trans*-basal filament, formation of *cis*-dimers, emergence of *cis*/*allo* tetrameric intermediates, nucleation of an activated octameric stack, and subsequent elongation into the *allo*-activated stacked filament. Together, these additions provide a direct visual framework for interpreting the quaternary transitions and support the assembly–disassembly–reassembly logic, which is central to our model shown in Figure 8.

6. ***Include more recent and relevant references on enzyme filamentation and its functional/regulatory roles in both Results and Discussion to frame the findings.***

We replaced the outdated references in the Discussion for more recent and relevant ones on functional/regulatory roles of enzyme filamentation.

Response to the **Reviewer #3** comments:

1. ***The first ~40 amino acids of full-length CBS have never been observed in previously reported structural data, both crystallographic and cryoEM data. This intrinsically disordered region has been shown, by NMR spectroscopy, to be able to accommodate a b-type heme (Kd ~2μM), with cysteine and histidine ligands, in a cysteine-proline motif [https://doi.org/10.1038/s41598-018-20841-z]. However, the function of this region and the relevance of its heme-binding ability remains unknown. Since the deposited PDB files are still pending publication, I could not figure out the sequence coverage of each structure herein reported and thereby understand whether these structures contain any information about this intrinsically disordered region. The only mention I could find of it in the manuscript is the term IDR in Figure 1A. So, my point is that, since it is indeed a structural element of CBS, it should be mentioned in the manuscript whether there is any density indicative of its position, and if in the presented models of the different oligomeric arrangements it is likely this domain could participate in any interaction between protomers (from the presented structures, it seems unlikely, but I think the reader should be given that information).***

Unfortunately, none of our cryoEM structures have the intrinsically disordered N-terminus resolved as well. Based on the published cryoEM structures (https://doi.org/10.1038/s41467-024-46864-x), we did not expect to see the first ~40 residues in *trans*-basal or *cis*-basal CBS filaments. However, we were hopeful to resolve it in the *allo*-activated stacked CBS filament as we were hypothesizing that this fragment may be involved in crosslinking the octameric stack and filamentation. However, low resolution did not allow us to draw any conclusions about the conformation of the catalytic domains as well as the intrinsically disordered N-terminus. Since our structures did not bring any new structural insights into the CBS N-terminal heme-binding domain compared to previously published crystal structures (e.g. https://doi.org/10.1093/emboj/20.15.3910, https://doi.org/10.1021/bi026052d, https://doi.org/10.1073/pnas.1313683110, https://doi.org/10.1155/2017/8940321), we did not elaborate further about this domain in the manuscript. We mention this now in the manuscript.

2. ***A minor technical point: on page 5, line 157, it is stated that to obtain the trans-basal CBS dataset, the sample was diluted in a buffer containing 1 mM β-mercaptoethanol. What was the reason for that? And was β-mercaptoethanol also added to the samples to obtain the other datasets? (If so, it should be mentioned). This point is further expanded in comment 7.4.***

Human CBS has mutliple surface-exposed Cys residues among which the most important are C15 (located in the unresolved section of the N-terminus and participating in CBS cross-linking (https://doi.org/10.1172/jci85396) and possible serving as an axial ligand to the alternative heme binding site(https://doi.org/10.1038/s41598-018-20841-z)) and C272+C275 (being part of the CXXC oxidoreductase motif). While the very first structure of the human CBS catalytic core lacking the regulatory domain found this motif oxidized (https://doi.org/10.1093/emboj/20.15.3910), the competing group resolved CBS catalytic core with the reduced CXXC motif likely due to the presence of a reductant in the crystalization medium. Most importantly, the authors did not find any significant structural changes that could have been attributted to the oxidation status of these vicinal thiols (https://doi.org/10.1021/bi026052d). During CBS purifications, we keep the reducing conditions to prevent its cross-linking via C15 residue by either using b-ME, DTT or TCEP. Therefore, the presence of, in this case, 1 mM b-ME was in all buffers used to dilute concentrated protein stocks kept in 1 mM TCEP-containing buffer for cryoEM studies. The protein dilutions were the same for all obtained cryoEM structures. Therefore, we revised the text in the Material and methods section accordingly.

3. ***On page 10, line 349, it is mentioned that the ligands SAM, PLP and serine were manually docked in Coot into corresponding densities. Then, Supplementary table 1 mentions the presence of heme ligands. Was there density***

for the heme ligands (to what extent, i.e., always 1 heme per monomer?), or were the hemes added on between the Cys52 and His65 axial ligands? Please clarify in the manuscript.

Clear density for the heme cofactor was observed in all CBS conformations for which the catalytic core was resolved at sufficient resolution (i.e. all states except the SAM-induced *allo*-activated stacked assembly, where the core region remains low resolution). For the *trans*-basal and *cis*-basal maps, previously determined CBS crystal structures were used as starting models (e.g., PDB IDs 4COO and 4UUU) and were first rigid-body docked into the cryo-EM densities. In these fits, the heme group and its axial coordination were fully consistent with the map, supporting one heme per CBS monomer, coordinated by C52 and H65. During subsequent refinement, the heme was retained with appropriate geometric restraints in Phenix real-space refinement, and the coordination geometry was manually inspected in Coot.

In the same initial docking step, the PLP cofactor was also positioned according to the template structures, and the density supported the expected covalent linkage to the catalytic lysine (K119) in the substrate-free state. In our models, the internal aldimine PLP adduct is represented as the modified residue LLP, reflecting the covalent PLP–lysine Schiff base. Thus, for each resolved monomer, the models contain one heme and one PLP cofactor, with the PLP covalently linked to K119 in the internal aldimine state.

Other ligands required additional, ligand-specific modeling. SAO and SAM were generated from the standard PDB chemical library (SMILES-based coordinates), individually docked into the corresponding densities at the S2 site, and then refined iteratively in Coot followed by Phenix using appropriate stereochemical constraints. For the PLP-derived reaction intermediates, we relied on high-resolution reference structures from the Drosophila CBS to guide assignment of the chemical species and covalent geometry (e.g., aminoacrylate:pdb-3PC3 and external-aldimine:pdb-3PC4). The cryo-EM densities in our substrate-bound datasets were of sufficiently high resolution to distinguish the different PLP adduct states, enabling identification of the external aldimine and aminoacrylate intermediates. These modified PLP ligands were then manually adjusted in Coot to match the density and refined in Phenix with covalent and geometric restraints, with particular attention to bond geometry, planarity, and local coordination around the PLP adduct.

We have now clarified these modeling procedures in the Materials and Methods section, including the approporiate mentions of the density supported direct placement (heme/PLP in resolved catalytic cores) versus the iterative docking and restraint-guided refinement (SAO, SAM, and PLP-derived intermediates).

4. ***On page 14, lines 525-527, and supported by Figure 1D, Y518 is singled out as being crucial for the described assemblies, by inserting into a hydrophobic pocket. I think it should be mentioned in the Results and/or Discussion the degree of sequence conservation of this residue across species. Lines 720-722 clarify that this filamentation ability may be species-specific, but assigns the lack of filamentation in mouse CBS to a longer oligomerization loop (similarly to human ISO2 CBS). It could be interesting to assess the conservation of this Y518 residue (and the shorter oligomerization loop), which should in principle allow predicting which CBS from which species can adopt this filamentous assembly.***

In our view, both the length and the residues of the oligomerization loop are important for the CBS filamentation to occur (as it is now further supported by additional data shown in Supplementary figures 4 and 5). To provide further support, we generated a sequence alignment for a limited number of representative CBS proteins focusing on this region within the RD. As new Supplementary figure 22 shows the Y518 residue as well as the length of the loop seems to be quite conserved among mammals. However, in some species, such as rat or mouse, the major CBS isoform is the one similar to human CBS ISO2, i.e. with an elongated oligomerization loop, which instead of a bulky aromatic tyrosine introduces a small hydrophilic serine and thus disrupts CBS filamentation (in addition to the elongated loop). We incorporated this new insight into the Discussion accordingly.

5. ***From Figure 7, I am unable to perceive whether the Authors were able to find density for SAM in the core allo-dimers. On Supplementary Table 1, SAM was only modelled in 9SR6, but it's unclear whether the data provide evidence for SAM binding to both cis- and allo-dimers. I understand that the resolution for this arrangement may preclude obtaining unequivocal densities for SAM, but this should anyway be clarified in the manuscript.***

We apologize for the confusion caused by the original presentation of Supplementary Table 1. The PDB ID 9SR6 model corresponds to a focused, single-particle refined structure of the SAM-bound *cis*-basal like conformer (associated with EMD-55133 and EMD-55132), and its placement in the table has now been corrected.

For the *allo*-activated stacked CBS assembly, our best reconstructions are EMD-55128 and EMD-55130. In these maps, the resolution in the stacked core and along the RD filaments is not sufficient to model SAM with the same confidence as in the *cis*-basal structures; therefore, we did not include SAM in the deposited atomic model for the *allo*-stacked state to avoid overinterpretation. Nevertheless, we performed an additional focused refinement on a single RD filament segment using a spanning mask, yielding a map at ~6 Å resolution, in which density consistent with SAM at the S2 site is apparent. We present this rigid-body fit and the corresponding density for review here, while explicitly noting that SAM placement in the *allo*-state remains suggestive but not modeled in the deposited coordinates due to limited resolution.

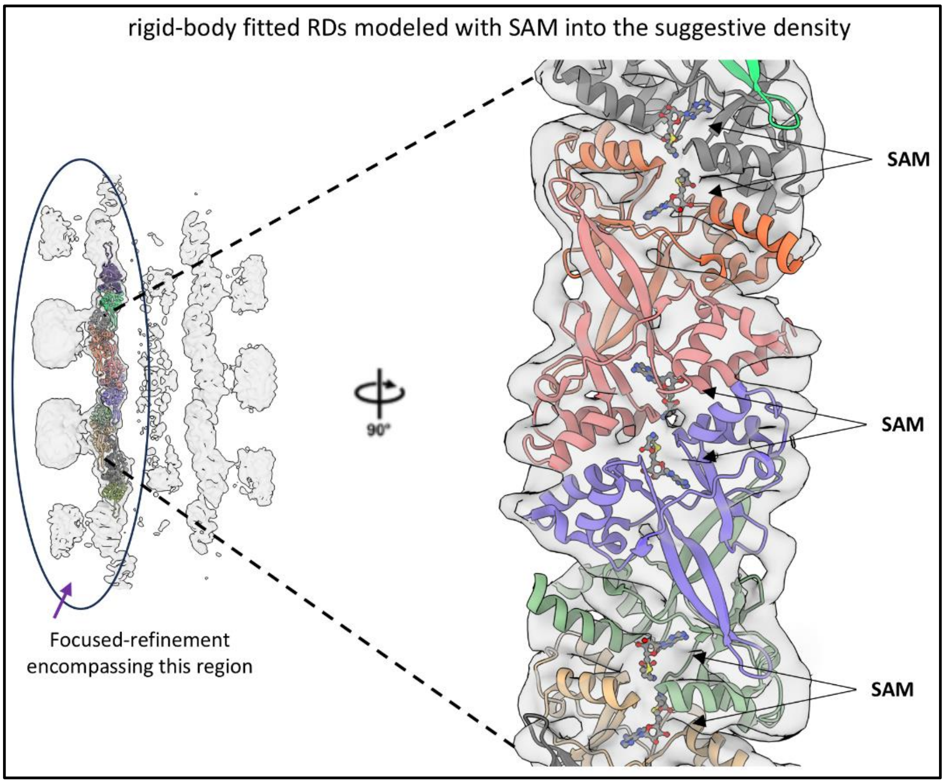

By contrast, the peripheral catalytic domains flanking the RD filaments are *cis*-like and better defined; therefore we retained heme and PLP in these regions because they are intrinsic cofactors of each CBS monomer and their positions are well supported by higher-resolution CBS structures. We have clarified this in the Materials and Methods section now that SAM is confidently modeled only in the *cis*-basal SAM-bound maps, whereas in the *allo*-stacked assembly, SAM binding is supported by module compatibility and focused-map density but is not included in the deposited model.

6. ***Still related with the previous comment, I think the Authors can slightly improve the clarity of Figure 7 in what concerns the bottom-right part of the figure, where allo-activated stacked CBS filaments are depicted. As it is, it is not immediately clear to which protomer each CD and RD from the core allo-dimers belong. So, as in Figure 5D, the Authors could simply keep one dark red + dark green protomer and make the other protomer pink (light red) and light green. This should make it clearer both in the octameric stack and in the filament.***

The color coding of the CBS protomers in the schematic/model now shown in Figure 8 view has been updated according to the reviewer's suggestion and to match color coding shown in Figure 6.

7. ***CBS is a fascinating enzyme, from different viewpoints, regarding its structure, function and regulation. I think the Authors could enrich the Discussion by explaining how these filamentous assemblies reconcile with previous data on CBS regulation:***

7.1. ***In comment 1, I already alluded to the N-terminal IDR which should be mentioned.***

We understand and share the Reviewer's fascination with the CBS structure, function and regulation. However, as we already explained in our response to the reviewer's comment #1, our structures did not resolve the first ~40 residues of the N-terminus, thus no new information and insight have been generated related to this region. Therefore, we have respectully refrained from the unsupported speculations.

7.2. ***Concerning the heme moiety, it has been shown, mostly by Ruma Banerjee's group, to regulate CBS activity through its redox and ligand state [PMID: 18278872, 19232736, 19733148, 22977242, 23790103, 24416422, 26867575, 27365395, 16505479, 15581573]. We have further demonstrated that SAM binding renders CBS more prone to become inactivated by CO and NO [https://doi.org/10.1074/jbc.m115.681221]. This allosteric regulatory mechanism has been proposed to be mediated by a helix spanning from the heme 'surroundings' to the PLP active site, with emphasis on Arg266 that is H-bonded to the heme ligand Cys52 [https://doi.org/10.1074/jbc.m112.414706]. It would be useful to perceive how the heme-binding region and the Thr257-Arg266 helical segment in these filamentous CBS assemblies (basal SAM-free and SAM-bound) are compatible with the heme-based regulation.***

Across all high-resolution reconstructions where the catalytic core is well resolved (i.e. *trans*-basal, *cis*-basal and the serine-trapped internal/external aldimine and aminoacrylate states), superposition shows that the heme-binding region and the T257–R266 segment are essentially unchanged, with the catalytic cores adopting the canonical CBS fold and the heme consistently coordinated by C52 and H65. In particular, the positioning of R266 relative to C52 is conserved in all these states, indicating that filament formation and substrate turnover do not disrupt the established heme-to-PLP communication framework. The only feature we can tentatively note from the substrate-bound high-resolution maps is a possible rotameric adjustment of T257 associated with PLP/serine interactions, but this appears to reflect local active-site chemistry rather than a change linked to heme redox or ligand sensing. In the SAM-induced *allo*-activated stacked assembly, the central core is not resolved at sufficient resolution to allow assessment of subtle heme-environment rearrangements, so heme-based modulation of the stacked state remains an important question for future work combining controlled heme ligation/redox conditions with higher-resolution structural and spectroscopic analyses.

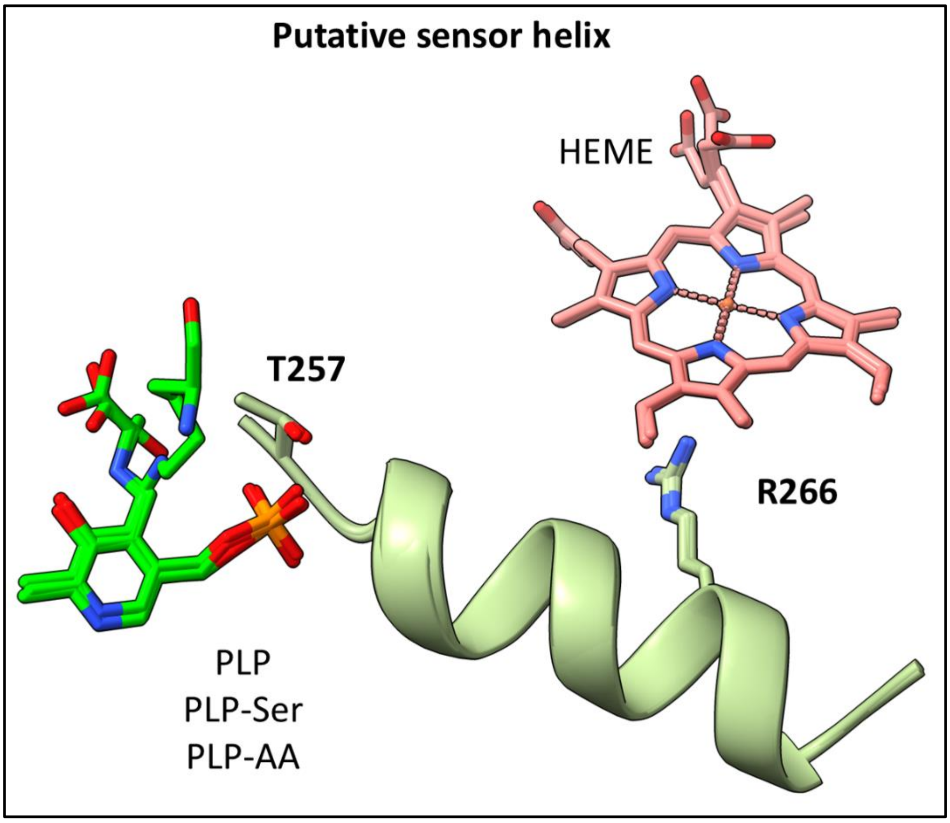

7.3. {missing/skipped}

7.4. Along the same lines, Niu and co-workers have demonstrated allosteric control of CBS activity by a surface exposed disulfide bond between Cys272 and Cys275 [https://doi.org/10.1074/jbc.ra117.000103]. Since the Authors used 1 mM β-mercaptoethanol in their sample preparation, this has likely resulted in reduction of the 'resting' disulfide bond and therefore prompted an already 2-3-fold pre-activated CBS. In light of this, it would be interesting to disclose the position of these cysteine residues in the obtained structures, particularly those with higher resolution, and comment on how the filamentous CBS assemblies still allow for this additional regulatory switch.

In our almost two-decades-long experience with CBS, we haven't observed and haven't been able to reproduce published data of other (such as the study mentioned by the reviewer) on the regulation of human CBS activity by a simple presence of a reductant. Regarding C272 and C275 being parts of the human CBS CXXC oxidoreductase motif, there is no significant structural change in CBS related to the oxidation status of these vicinal thiols (as we also mentioned in our response to the reviewer's comment #2). In the paper of Niu et al. (https://doi.org/10.1074/jbc.ra117.000103), the authors claim that the treatment of CBS with 10 mM DTT increased activity by 2-3-fold due to the reduction of the CXXC motif. However, the previous study from Ruma Banerjee lab (https://doi.org/10.1074/jbc.273.39.25179) showed that addition of 5 mM DTT did not alter CBS activity under anaerobic conditions. Furthermore, anaerobic conditions themselves were shown to routinely enhance CBS activity by 1.4-fold. Although the underlying reasons remain unclear, the authors hypothesized that it could have been due to the protection of the substrate homocysteine from oxidation or due to the oxidative instability of CBS itself. Our experience supports the former as only the reduced homocysteine acts as the CBS substrate (https://doi.org/10.1016/j.redox.2024.103383). Thus anaerobic conditions (particularly in the absence of an additional reductant or the presence of transition metals) would prevent a decrease of effective substrate concentrations, which could consequently enhance activity. Therefore, this process is more technical in nature than biological or physiologically relevant.

A similar case could be argued for another accessible cysteine residue C15 supposedly involved in additional heme cofactor binding (serving as an axial ligand together with residue H22) and whose removal (as the N-terminal ~40 residues truncation) or mutagenesis into serine (C15S) would decrease CBS activity by cca 32% (https://doi.org/10.1038/s41598-018-20841-z). Data from Jan Kraus lab show that (i) C15 residue is not involved in heme binding as removal of the first ~40 residue from CBS polypeptice did not affect the heme spectrum associated with the protein (https://doi.org/10.1006/abbi.1998.0723), (ii) C15S mutation does not affect CBS activity at all (https://doi.org/10.1021/bi060737m), but rather (iii) effectively prevents oxidation-based aggregation and cross-linking of CBS (https://doi.org/10.1172/jci85396). As also explained in our response to the reviewer's comment #2, we used reductant to avoid this cysteine-based cross-linking in our cryoEM structural studies.

7.5. CBS has been shown to accumulate in mitochondria, in a process mediated by Lon protease [https://doi.org/10.1073/pnas.1308487110]. Herein, the Authors show accumulation of CBS filaments close to the nucleus. I know it may be a stretch to speculate on that, but it would be interesting to discuss how the filamentous CBS data can accommodate this re-localization phenomenon.

The study by Teng et al. (https://doi.org/10.1073/pnas.1308487110) indeed reported on CBS accumulation in mitochondria under hypoxic conditions regulated upon oxygenation by Lon protease. In addition to the fact that we were unable to reproduce any results from this study, it has several flaws. Banerjee, Kruger and Kraus labs all showed that the expression of heme-impaired CBS (e.g. CBSΔ1-70, CBS C52A, CBS C52S or CBS H65R or CBS WT in heme-free system) results in seriously unstable, likely misfolded, catalytically-impaired CBS enzymes, which could be partially alleviated by the presence of a fusion partner, such as GST, alternative metalloporphyrin or chemical chaperone (https://doi.org/10.1021/bi011827o, https://doi.org/10.1074/jbc.m805928200, https://doi.org/10.1016/j.abb.2004.04.027). Teng et al. showed that these heme-impaired CBS variants (artificial CBSΔ1-70 and CBS C52A and patient-derived pathogenic CBS H65R) accumulate in mitochondria. Therefore, enrichment of such misfolded/aggregated/degraded CBS polypeptide in various subcellular fractions would have to be interpreted very cautiously. Furthermore, the lack of the first 70 residues (~13% of CBS length) corresponds to a

theoretical loss of 7.5 kDa (i.e. 60.6 kDa CBS WT compared to 53.1 kDa CBSΔ1-70). However, on the Western blots shown in the paper the difference in migration of CBS polypeptides is much smaller (especially when considering that the type of PAGE gels was likely selected to emphasize or at least to show this difference) and size-wise correlates with either a CBS degradation band or some unknown anti-myc antibody non-specific band. Since the myc tag was positioned on the CBS C-terminus, the lower band may correspond to the N-terminally-degraded CBS as shown previously (https://doi.org/10.1006/abbi.1998.0723). Lastly, the model proposed by the authors stipulates that de-oxygenated CBS accumulates in mitochondria during hypoxia and when it is oxygenated (by normoxia or oxidative stress), it is recognized by Lon protease and degraded. First, oxidized ferric heme of CBS is essentially biologically inert and does not bind any additional ligands other than C52 and H65 axial ligands (e.g. https://doi.org/10.1074/jbc.273.39.25179, https://doi.org/10.1021/bi701159y, https://doi.org/10.1006/bbrc.2001.4807) unless the ferric heme is reduced to ferrous. Second, reduction to ferrous heme can experimentally be achieved only by the excess of very strong reductants, such as sodium dithionite or titanium citrate, due to very low (negative) reduction potential of around -300 mV (https://doi.org/10.1021/bi700912k). Third, ferrous heme CBS has the same activity as ferric heme CBS, but its lower activity observed multiple times and considered as a regulatory mechanism actually stems from the gradual irreversible loss of ferrous CBS activity due to heme ligand exchange/switch yielding catalytically inactive CBS424 species (named after a typical spectral shift of CBS Soret peak, https://doi.org/10.1021/bi701159y). Fourth, CBSΔ1-70 variant would not be able to undergo heme deoxygenation and oxygenation with subsequent degradation by Lon protease as the authors of the study suggested simply because it does not contain heme at all (both C52 and H65 axial ligands are missing and heme cannot be retained by CBS polypeptide and buried deeper into its structure as the authors mistakenly hypothesized). Taken together, we don't feel confident about the results of this study showing mitochondrial localization of CBS and, therefore, would refrain from giving it attention by discussing our results.

Having said that and going back to the reviewer's question, CBS filamentation can likely occur in any cell compartment where the full-length CBS is present. Our imaging studies were not focused on CBS localization, but rather on the overall distribution pattern as non-filamentous forms would likely yield more homogeneous distribution than the filamentous ones. Our data are in line with those reported previously by McCorvie et al. showing punctual localization for filamentous CBS WT and homogenous distribution for filamentation-deficient CBS variants (https://doi.org/10.1038/s41467-024-46864-x). Whether CBS WT co-localizes with mitochondria or other cellular organelles or just forms a complex in the cytoplasm would have to be determined by future studies.

Response to the reviews of the manuscript ID# NCOMMS-25-87465A:

Dear Colleagues,

Thank you for your thorough evaluation of our manuscript and for the constructive comments and suggestions. Below please find a point-by-point response to each of your specific comments. We have revised the manuscript as outlined in our response. All the revisions and changes are highlighted in a tracked copy of our revised manuscript alongside a clean revised manuscript.

We hope that you will find our revision in response to your reviews acceptable and that you will be able to recommend the revised manuscript for acceptance to the Editor.

Response to the **Reviewer #1** comments:

1. ***For figure 1. It's great that the authors made figure 1 to illustrate the methionine metabolism pathway. I like it. But I recommend the authors could move the figure 1 to the supplemental figures since figure 1 doesn't provide new data. And this could reduce the main figure number too.***

Although Figure 1 does not provide new data, it is quite illustrative and puts the key role of CBS in perspective within sulfur amino acid metabolism. The editorial office also indicated that they don't deem it essential to move the figure into the supplement, as they don't have any issue with the current number of main figures of the manuscript. Therefore, we hope it is fine with you if we keep Figure 1 as it is after we prepared it and added it to the revised manuscript specifically in response to your previous comment/suggestion. It will also save us from renumbering essentially every main and supplementary figure.

Response to the **Reviewer #2** comments:

1. ***First, while the placement of the section "Substrate binding reveals catalytic rearrangements of the active site" is ultimately at the authors' discretion, I find that it interrupts the flow of the Results section and detracts from the main message. I would recommend moving it to either the beginning or the end of the Results section to improve clarity and coherence.***

We appreciate the recommendation to improve clarity and coherence; however, we see it from a different perspective. We intentionally put it before any sections describing allosteric ligand-bound conformations as substrate-bound states were evaluated and resolved only in the basal allosteric-ligand free conditions. Therefore, putting it at the end of the Results would, in our view, disrupt this intended flow. Thus, we respectfully keep its position as it is.

2. ***Second, it would be helpful to clarify in the main text how SAM-induced structural rearrangements result in the His-tag becoming buried or otherwise inaccessible for interaction with the Ni-NTA matrix.***

We believe this comment refers to the experiment shown in Figure 7B, whose purpose was to demonstrate a solution-based support for the morpheein-style dynamics of CBS. Specifically, the experiment was designed to show that in the absence of SAM, CBS predominantly exists as the *trans*-basal filaments, whereas upon addition of SAM, these assemblies must first disassemble into subunits or smaller intermediates (dimers), undergo conformational rearrangement into *cis*- and *allo*-dimers and then reassemble back yielding *cis*-basal filament (minor fraction in case of SAM, but the only fraction in case of SAO) and *allo*-activated stacked filament (major fraction in case of SAM). Importantly, this was intended as a simple and indirect solution-based experiment to support the morpheein model, because cryo-EM provides structural snapshots of the already formed *cis*-basal and *allo*-activated stacked states, but cannot directly resolve the rapid disassembly/reassembly process itself. As shown in Figure 7B, in the absence of SAM, only a negligible amount of CBS gets released into the supernatant after the saturation of the Ni-NTA beads. In contrast, in the presence of SAM, a transient fraction of CBS appears in the supernatant consistent with disassembly of the resin-bound *trans*-basal filaments, followed by rearrangement and reassembly into SAM-induced conformations that subsequently re-bind to the Ni-NTA beads.

Thus, rather than indicating burial or inaccessibility of the His tag, this experiment suggests the opposite: the C-terminal 6xHis tag remains solvent-accessible in the SAM-induced conformations, because otherwise we would not observe efficient re-binding to the Ni-NTA beads and subsequent elution in amounts comparable to CBS in the absence of SAM. Based on our current solved structures (*trans*-, *cis*- and *allo*-CBS models using CBS construct without any tag) and their interpretation, the C-terminal His-tag, located on the regulatory domain, remains sufficiently exposed on the outer surface of the assemblies and therefore accessible for interaction with the Ni-NTA resin.

The figure below highlights the last few residues resolved at the C-terminus in selected CBS subunits forming SAM-bound *allo*-activated stacked CBS filament. This structure resolved the C-terminus except the last one or two residues (i.e. …VAAQERD resolved vs …VAAQERD**QK**/VAAQERDQ**K** annotated sequence). Our His-tagged CBS construct contains two additional amino acid residues serving as a linker followed by the 6xHis tag (i.e. …VAQERDQK**LEHHHHHH**). Therefore, adding 1+2=3 or 2+2=4 residues as a linker connecting the resolved sequence/structure with the 6xHis tag would certainly lead to the tag being solvent accessible and available for binding to the Ni-NTA resin:

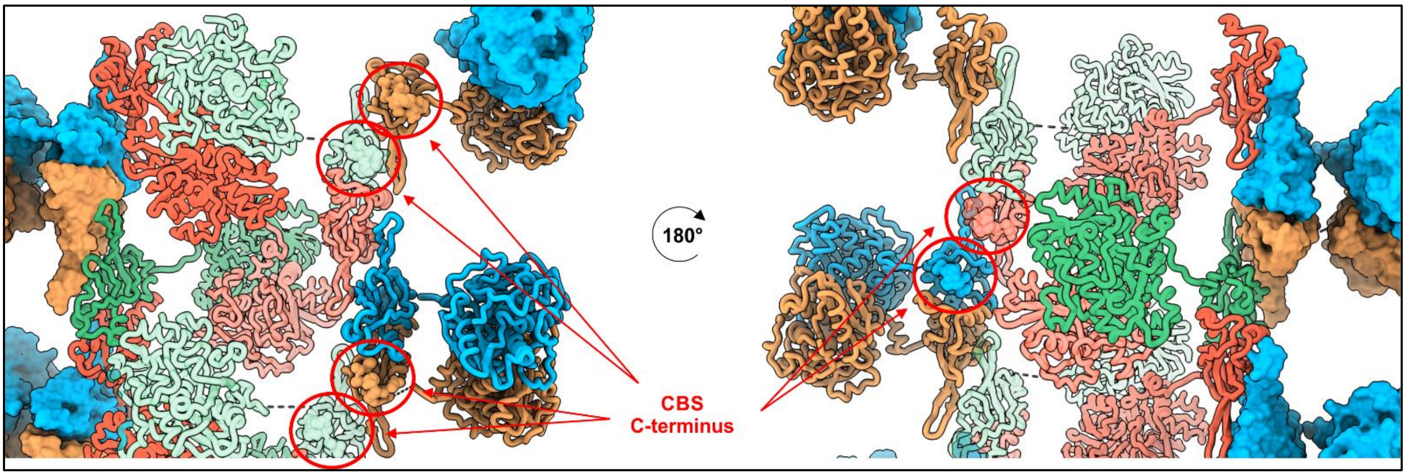

Just to support our conclusion even further, we performed a similar experiment as shown in Figure 7B with the dimeric CBSΔ516-525 carrying the C-terminal 6xHis tag (the region is sequence-wise identical to the full-length CBS described above). The experiment showed that dimeric CBSΔ516-525 does not release CBS protein into the supernatant in appreciable amounts upon addition of SAM, indicating that, unlike larger oligomers or filaments, dimeric CBSΔ516-525 can conformationally rearrange (presumably from *trans*- to *cis*-dimers) without detaching from the Ni-NTA beads. These results further support morpheein-style dynamics of filamentous CBS:

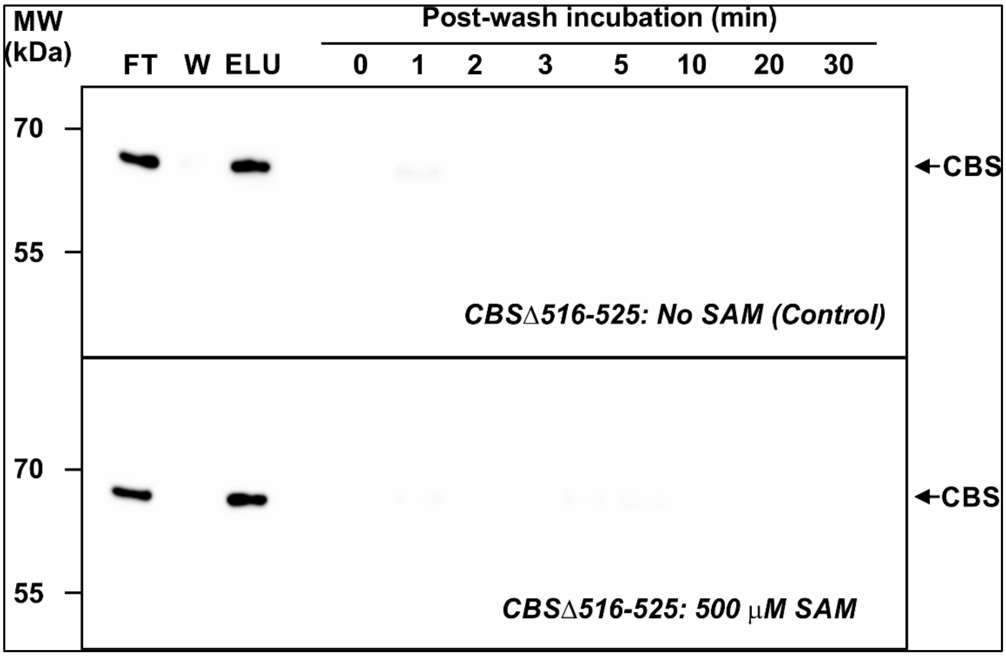

While avoiding overinterpretation, the explanation provided here has been briefly incorporated into the revised manuscript.

Note:

Our structures cannot structurally rationalize the accessibility of the C-terminal His tag as the protein used for structural studies did not contain the tag. McCorvie et al. (PMID 38575566) used both non-tagged (actually, N-terminal His tagged removed by TEV protease treatment and purification) and C-terminal His-tagged CBS for cryoEM, but did not observe any interpretable densities in the maps for the tag. However, they showed the His-tagged CBS presented longer filaments, higher stability and less degradation with His tag affecting the entropy and enthalpy of the first SAM binding event in ITC (i.e. the actual binding to the CBS domains based on our proposed explanation of ITC profile rationalizing binding stoichiometry and conformational/oligomerization changes). Their findings can alternatively be explained by the purification enrichment for the full-length CBS and the overall purity of the preparations. The C-terminal His tag compared to the N-terminal one should theoretically promote enrichment of a full-length protein using immobilized metal affinity chromatography (IMAC). In the case of CBS, the main "impurity" during its preparation is the incomplete/truncated/degraded CBS with a shortened or completely missing regulatory domain (thus yielding SAM-insensitive, constitutively activated, dimeric truncated CBS). The amount of this "impurity" in non-tagged CBS is at least doubled compared to the His-tagged CBS in preparations used by McCorvie et al. (based on visual inspection of their Supplementary figure 2A). In the figure below, we want to illustrate the variable content of this "impurity" in two CBS WT preparations (labeled as #1 and #2) and the fact that this impurity is indeed CBS-derived (i.e. cross-reacting with anti-CBS antibody) showing that CBS WT preparation #1 is clearly inferior in terms of quality to #2:

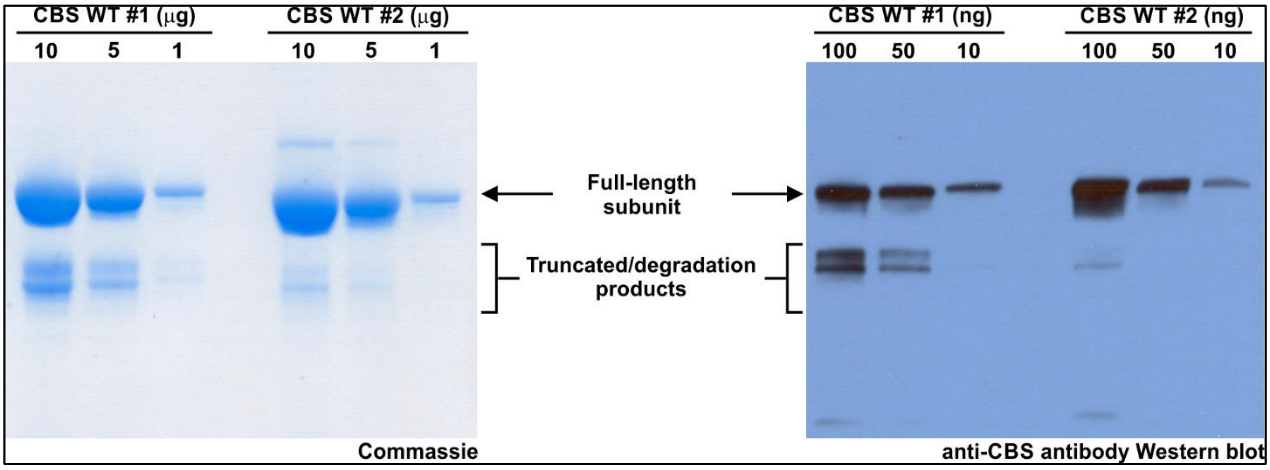

Structural work presented in this manuscript was carried out using CBS WT #2 preparation, which purity is superior to that of the CBS WT #1 as well as those used by McCorvie et al. (PMID 38575566) despite having no C-terminal His tag. Note that the higher amount of this "impurity" typically manifests in activity assay as higher basal activity (in the absence of SAM) and lower activation fold increase after the addition of SAM. In structural work, it would manifest with shorter filaments (as their elongation is disrupted by the missing regulatory domain in the partially degraded dimers with one subunit as a full-length and other incomplete/truncated/degraded subunit), lower stability (McCorvie's paper Figure 4B correlating well with our data presented for dimeric forms CBS ISO2, CBSΔ516-525 and CBS45) and more degradation (higher content of incomplete/truncated/degraded subunit). It can be speculated that the purity issue could also be responsible for the fact that McCorvie et al. did not observe *allo*-activated stacked filaments in the presence of SAM.

3. ***Finally, the statement regarding the anticancer drug taxol could be refined for accuracy and clarity. Taxol is known to bind polymerized microtubules rather than dimeric tubulin, and it stabilizes microtubules rather than destabilizing them.***

We rephrased the sentence mentioning taxol as an example of a specific conformation-locking compound for accuracy and clarity.

Response to the Reviewer #3 comments:

1. ***On lines 67, 1307 and 1321 of the revised manuscript (and the new Figure 1), I suggest adding 'reverse' before 'transsulfuration pathway', in line with what should be the formal designation of the mammalian transsulfuration pathway converting methionine to cysteine.***

The reviewer is technically correct about the nomenclature and we revised the text and the figure accordingly. However, since mammals have only the reverse pathway, it is very well accepted and established in the field to not use this distinction unless necessary (e.g. when working on bacteria, fungi, etc.).
